# Supplementary material for: Molecular profiles, sources and lineage restrictions of stem cells in an annelid regeneration model
Source: Nat Commun. 2024 Nov 18;15:9882. doi: 10.1038/s41467-024-54041-3 (PMC11574210; doi:10.1038/s41467-024-54041-3)
Supplement: Supplementary file 1 — Supplementary Information [file 41467_2024_54041_MOESM1_ESM.pdf]

Supplementary Information

Molecular profiles, sources and lineage restrictions of stem cells in an annelid regeneration model

Alexander W. Stockinger, Leonie Adelmann, Martin Fahrenberger, Christine Ruta, B. Duygu Özpolat, Nadja Milivojev, Guillaume Balavoine, Florian Raible

Supplementary Information includes:
Supplementary Figures 1-6

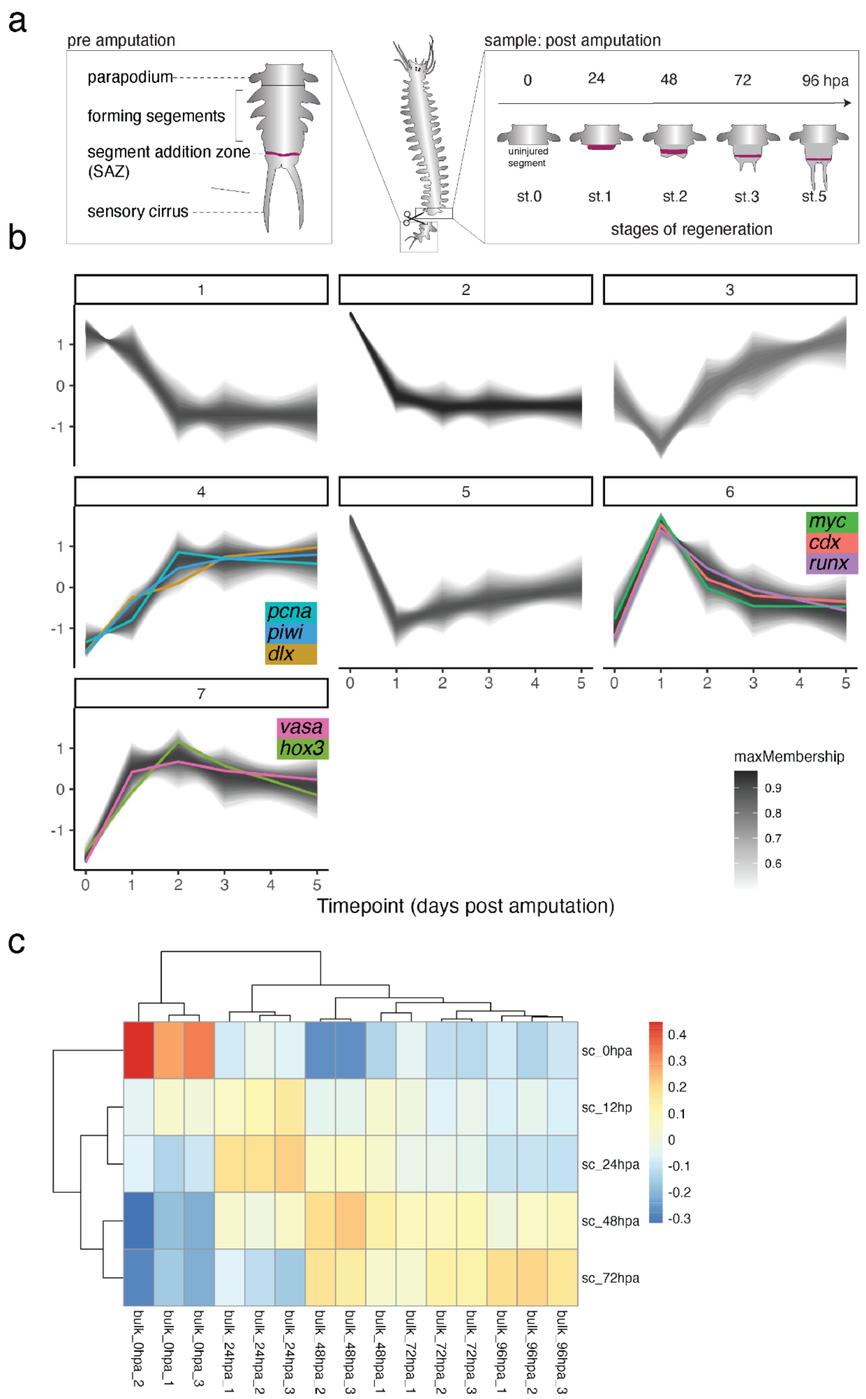

Supplementary Figure 1. Bulk RNA sequencing captures dynamics of gene expression during regeneration.
(a) Sampling scheme illustrating posterior amputation and sampling timepoints 0 to 5, ranging from 0 hours post amputation (0 hpa, equivalent to a regular trunk segment) to 96 hpa; **(b)** distinct mfuzz clusters grouping major variants of gene expression dynamics over the sampled time points. Colored lines indicate the assignment of individual candidate genes (*pcna*, *piwi*, *cdx*, *myc*, *runx*, *hox3*, *vasa*) to these clusters. All transcripts with an mfuzz cluster membership > 0.5 were included (gray colors). (c) Correlation matrix showing Pearson correlation scores of bulk sequencing (x-axis, labeled by timepoint post amputation and biological replicate) and single-cell sequencing data (y-axis, pseudobulked expression data labeled by timepoint post amputation) at all sampled timepoints.

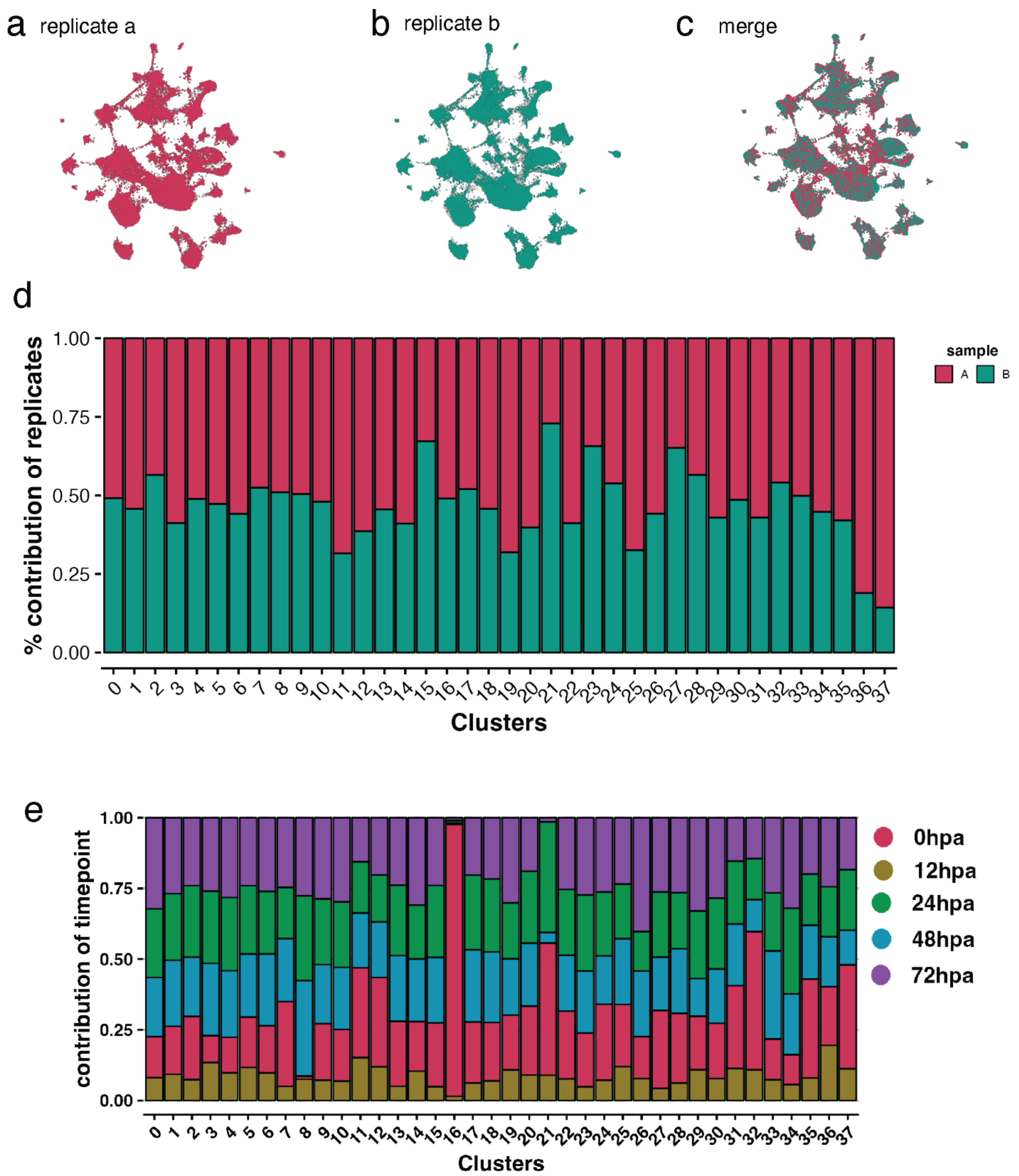

Supplementary Figure 2: Sampling timepoint and replicate contribution to single cell UMAP and clusters.
(**a-c**) UMAP visualization of independently replicated scRNAseq time series (merged timepoints 0 hpa - 72 hpa) both individually (**a-b**) and replicate a and b merged (**c**); (**d, e**) relative contribution of biological replicates (d) and sampling timepoints (e) to the clusters of this dataset.

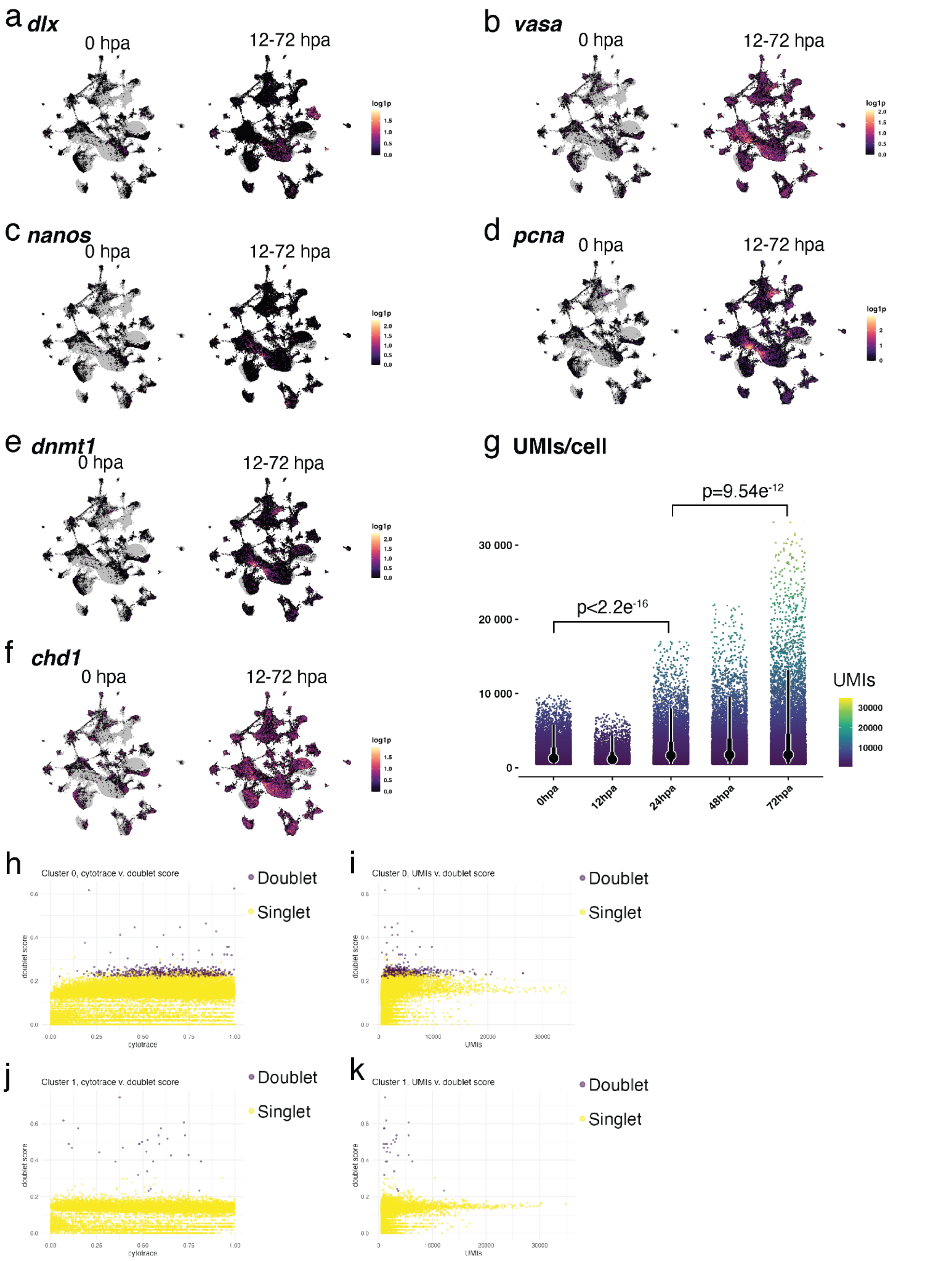

Supplementary Figure 3: UMAP visualizations and UMI analysis of gene expression dynamics during regenerative timepoints.

(**a-f**) gene expression UMAP visualizations of select PSC-related genes, splitting the dataset into 0 hpa (right after posterior amputation) and 12-72 hpa (post-injury timepoints). (**g**) geyser plot of UMIs per cell across sampled timepoints showing the progressive emergence of cells with significantly higher UMIs as regeneration proceeds (Wilcoxon rank sum test with continuity correction). (**h-k**) Scatterplots comparing CytoTRACE scores (h,j) and UMIs/cell (i,k), respectively, to predicted doublet likelihood scores in clusters 0 (h,i) and 1 (j,k), respectively.

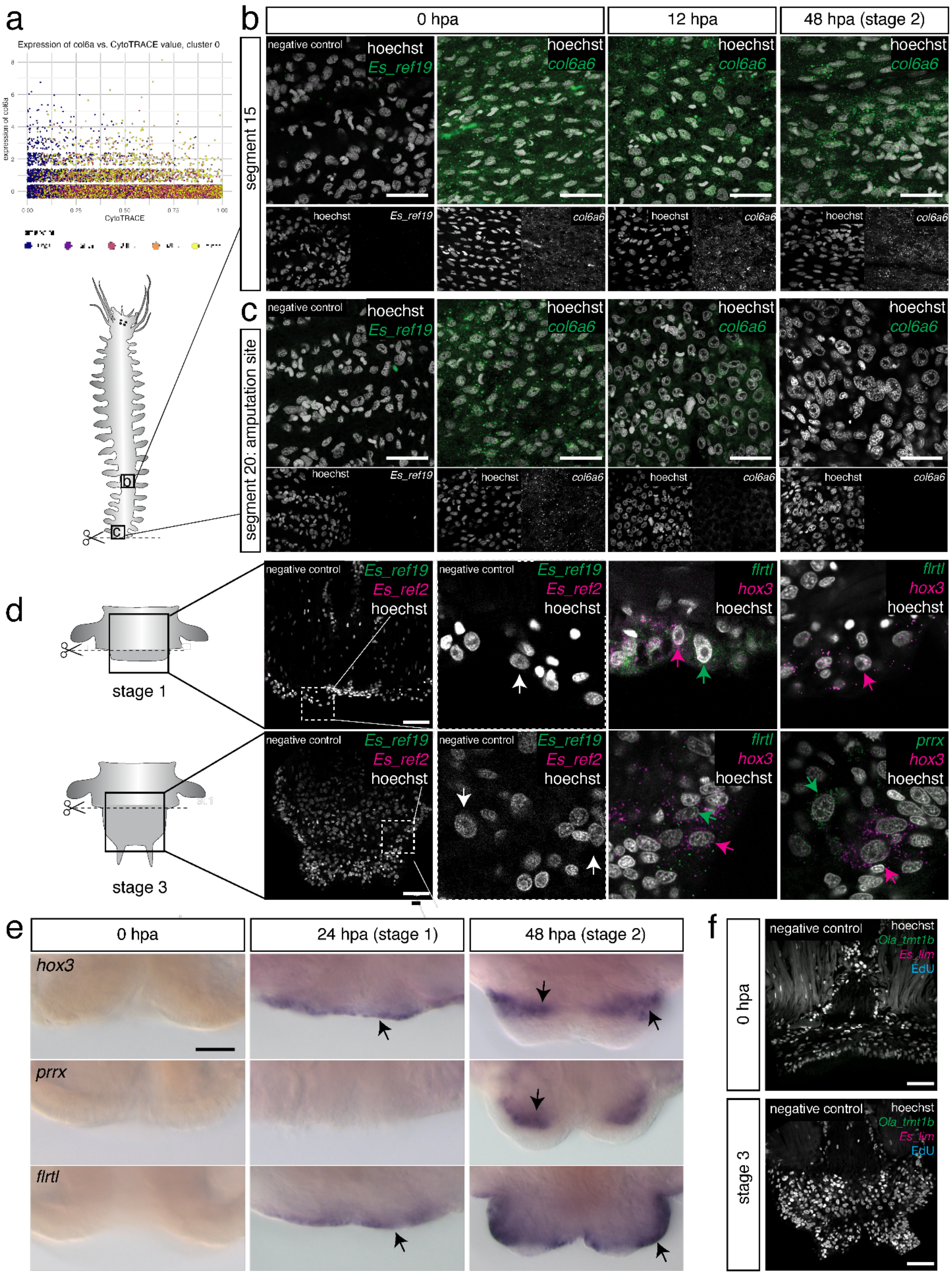

Supplementary Figure 4:

(**a**) Scatterplot showing the expression of *col6a6* compared to CytoTRACE scores, colored by sampling timepoint. (**b, c**) sampling scheme and confocal images of *in situ HCR* labelings of *col6a6* expression at wound-adjacent segment (c) and distant segment (b), sampled at different stages of regeneration, alongside negative controls (*Es_ref19*) and nuclear Hoechst label. Scale bar = 25µm. (**d**) sampling scheme and confocal images of *in situ HCR* labelings of *hox3*, *flrtl* and *prrx*, alongside negative controls (Es_ref19 and Es_ref2) and nuclear Hoechst label. Scale bar = 50µm. (e) Visualisation of *hox3*, *flrtl* and *prrx* expression by classic wholemount *in situ* hybridisation at 0hpa, 24hpa and 48hpa. Scale bar = 125µm. (f) HCR (Ola_tmt1b and Es_lim)and EdU negative controls for 0pa and stage 3 regenerate. Scale bar = 50µm.

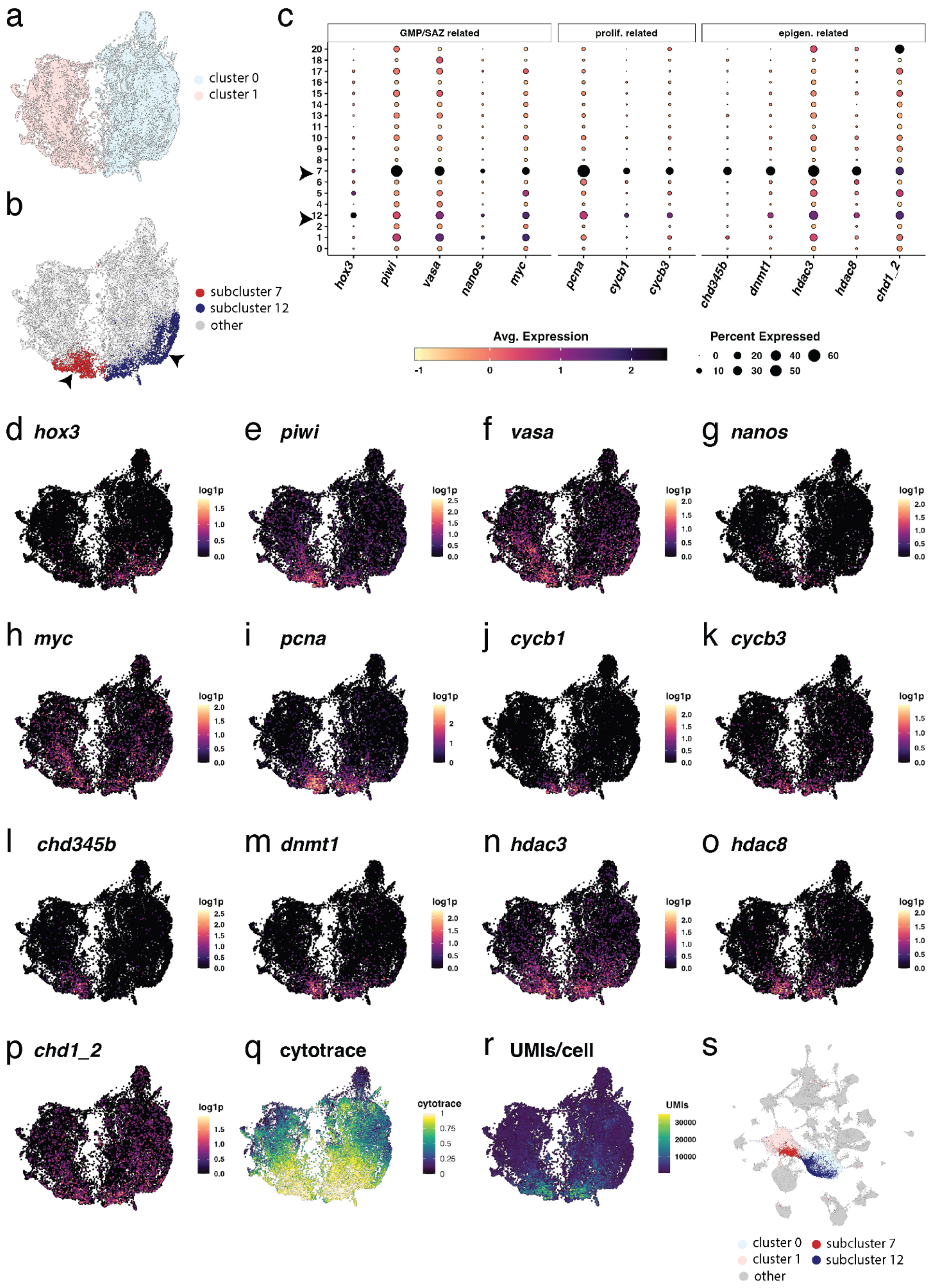

Supplementary figure 5. Subsetting clusters 0 and 1 to identify strongly PSC-related subclusters

(**a-b**) UMAP visualizations of subset clusters 0 and 1, with the newly identified PSC-related subclusters highlighted (**b**).
(**c**) dotplot representation of gene expression in subclusters of clusters 0 and 1, showing genes related to the GMP/SAZ, proliferation and epigenetic remodeling. (**d-p**) Individual UMAP visualizations of all genes shown in dotplot (c). (**q,r**) UMAP visualizations of CytoTRACE scores (q) and UMIs per cell (r). (**s**) UMAP visualization of the entire dataset, highlighting clusters 0 and 1, and their newly identified respective PSC-like subclusters 12 and 7.

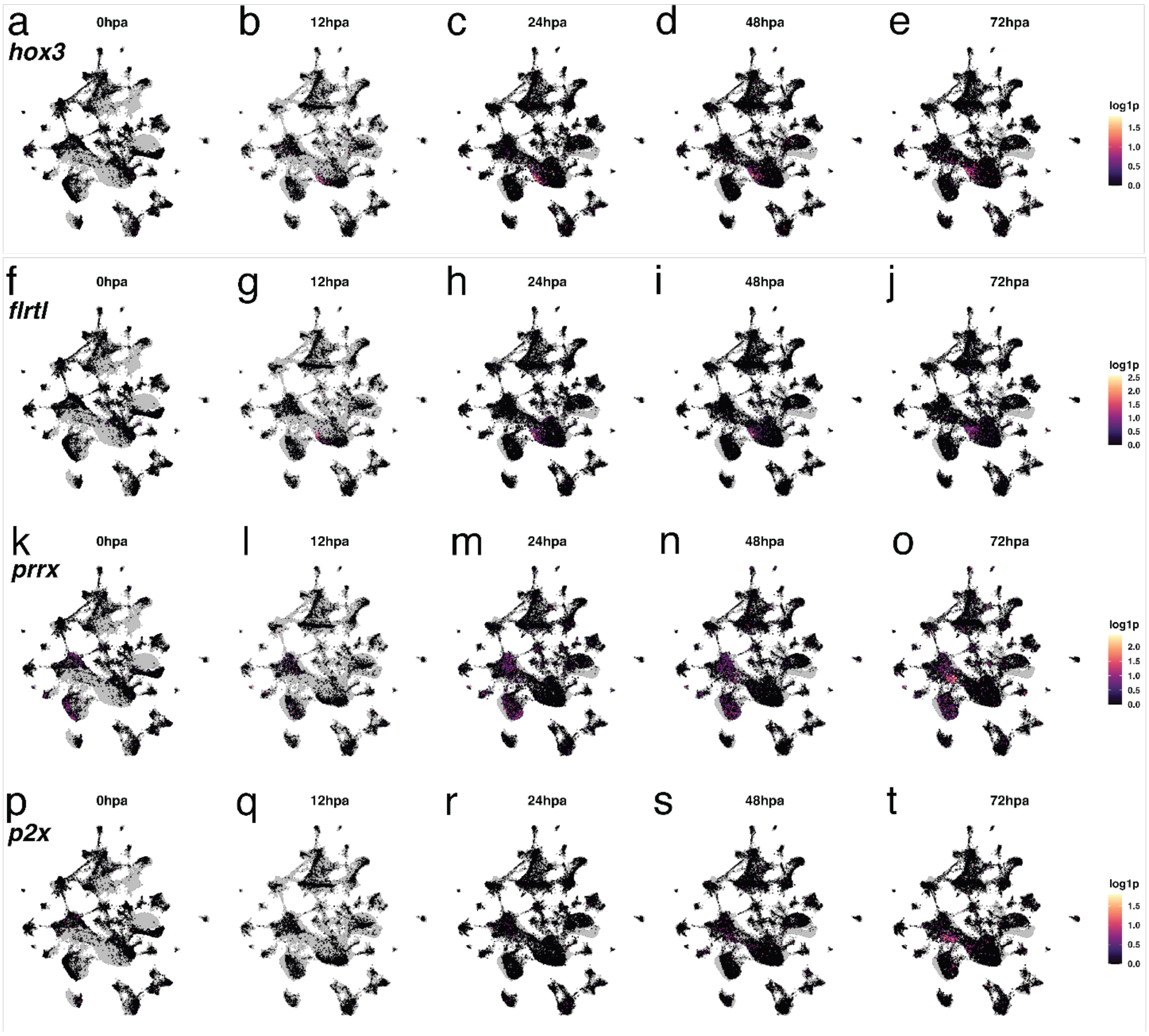

Supplementary figure 6: Synopsis of subcluster-enriched stem cell signatures.

(**a-t**) Time-resolved UMAP visualizations showing the expression of *hox3* (a-e), *flrtl* (f-j), *prrx* (k-o) and *p2x* (p-t) over the course of regeneration.
