# Supplementary material for: Molecular profiles, sources and lineage restrictions of stem cells in an annelid regeneration model
Source: Nat Commun. 2024 Nov 18;15:9882. doi: 10.1038/s41467-024-54041-3 (PMC11574210; doi:10.1038/s41467-024-54041-3)
Supplement: Supplementary file 3 — Description of Additional Supplementary Files [file 41467_2024_54041_MOESM3_ESM.pdf]

Description of Additional Supplementary Files

File Name: Supplementary Data 1
Description: Metrics for scRNAseq analysis.

File Name: Supplementary Data 2
Description: Information on candidate genes used for cluster annotation and analysis.

File Name: Supplementary Data 3
Description: Top marker genes for main clusters and for subclusters of clusters 0 and 1.

File Name: Supplementary Data 4
Description: Information on gene ontology terms used for cluster annotation and analysis.

File Name: Supplementary Data 5
Description: Target sequences for HCR probe design, and respective HCR probe sequences used in the study.

File Name: Supplementary Data 6
Description: Molecular phylogenetic analyses of selected genes.

File Name: Supplementary Data 7
Description: Collective information on transgenic clones, respective image acquisitions (including schematics), and synopsis of observed patterns and their transmission during regeneration.
