# Supplementary material for: Molecular profiles, sources and lineage restrictions of stem cells in an annelid regeneration model
Source: Nat Commun. 2024 Nov 18;15:9882. doi: 10.1038/s41467-024-54041-3 (PMC11574210; doi:10.1038/s41467-024-54041-3)
Supplement: Supplementary file 5 — Supplementary Data 2 [file 41467_2024_54041_MOESM5_ESM.docx]

# Supplementary data 2:

**Information on candidate genes used for cluster annotation and analysis**

| **gene** | **xloc** | **genbank** | **cell type** | **reference** | **clusters** |
| --- | --- | --- | --- | --- | --- |
| *a-amylase* | XLOC_002486 | KM577675 | midgut | [1](https://app.readcube.com/library/7fac8260-73e5-4965-a22c-0410b504e320/all?uuid=8690286768973577&item_ids=7fac8260-73e5-4965-a22c-0410b504e320:21a4dd2e-16ac-43c9-93fe-e15c0da9b994) | 21 |
| *calponin* | XLOC-009350 | - | smooth muscle | [2](https://app.readcube.com/library/7fac8260-73e5-4965-a22c-0410b504e320/all?uuid=535450687147629&item_ids=7fac8260-73e5-4965-a22c-0410b504e320:8b219fe4-4be7-46c5-be56-a59daa2ac607) | 0 8 14 |
| *coe* | XLOC-028320 | GU169416 | neuron | [3](https://app.readcube.com/library/7fac8260-73e5-4965-a22c-0410b504e320/all?uuid=7110607230003563&item_ids=7fac8260-73e5-4965-a22c-0410b504e320:941b2cfd-713c-46e0-a410-45d9ed3d6b5c) | 5 |
| *cs3* | XLOC-001874 | KJ405470 | chaetal sac | [4,5](https://app.readcube.com/library/7fac8260-73e5-4965-a22c-0410b504e320/all?uuid=626499346102694&item_ids=7fac8260-73e5-4965-a22c-0410b504e320:13a7cec8-9a94-4a1a-9e65-e6c01b144237,7fac8260-73e5-4965-a22c-0410b504e320:737c41aa-79e6-47ee-bba3-f0d64771ce2e) | 24 |
| *egb_a1a* | XLOC-020342 | MT701024 | globin secreting | [6](https://app.readcube.com/library/7fac8260-73e5-4965-a22c-0410b504e320/all?uuid=09303496734287608&item_ids=7fac8260-73e5-4965-a22c-0410b504e320:110bbcf8-09ee-4dee-b7cc-0f3b2a76bafd) | 15 |
| *egb_a1b* | XLOC-035593 | MT701025 | globin secreting | [6](https://app.readcube.com/library/7fac8260-73e5-4965-a22c-0410b504e320/all?uuid=9938787238683451&item_ids=7fac8260-73e5-4965-a22c-0410b504e320:110bbcf8-09ee-4dee-b7cc-0f3b2a76bafd) | 15 |
| *elav1* | XLOC-001549 | EF384209 | neuron | [7](https://app.readcube.com/library/7fac8260-73e5-4965-a22c-0410b504e320/all?uuid=2801726199758843&item_ids=7fac8260-73e5-4965-a22c-0410b504e320:6acd60cc-313c-41cd-9429-2fbda3a82222) | 5 11 18 20 |
| *foxA* | XLOC-060337 | AM114771 | hindgut; neuron | [8](https://app.readcube.com/library/7fac8260-73e5-4965-a22c-0410b504e320/all?uuid=23645780653479587&item_ids=7fac8260-73e5-4965-a22c-0410b504e320:a6d140a3-0c8f-46da-bf99-5968d1174347) | 4 11 20 |
| gata456 | XLOC-067983 | EF014969 | smooth muscle | [2](https://app.readcube.com/library/7fac8260-73e5-4965-a22c-0410b504e320/all?uuid=4560634682993535&item_ids=7fac8260-73e5-4965-a22c-0410b504e320:8b219fe4-4be7-46c5-be56-a59daa2ac607) | 8 10 12 17 |
| *gcm* | XLOC-004995 | HE971735 | gcm+ neuron | [9](https://app.readcube.com/library/7fac8260-73e5-4965-a22c-0410b504e320/all?uuid=6016762694931758&item_ids=7fac8260-73e5-4965-a22c-0410b504e320:0800d5e6-61c1-428b-91e5-3dc233b997c1) | 9 36 |
| *hb9* | XLOC-015560 | EF384221 | neuron | [7](https://app.readcube.com/library/7fac8260-73e5-4965-a22c-0410b504e320/all?uuid=6701158835279049&item_ids=7fac8260-73e5-4965-a22c-0410b504e320:6acd60cc-313c-41cd-9429-2fbda3a82222) | 4 5 11 16 |
| *legumain* | XLOC_037879 | KM577676 | gut | [1](https://app.readcube.com/library/7fac8260-73e5-4965-a22c-0410b504e320/all?uuid=8899138157773953&item_ids=7fac8260-73e5-4965-a22c-0410b504e320:21a4dd2e-16ac-43c9-93fe-e15c0da9b994) | 4 7 27 |
| *mef2* | XLOC-000048 | - | smooth muscle | [2](https://app.readcube.com/library/7fac8260-73e5-4965-a22c-0410b504e320/all?uuid=21727210495983285&item_ids=7fac8260-73e5-4965-a22c-0410b504e320:8b219fe4-4be7-46c5-be56-a59daa2ac607) | 2 6 12 |
| *myocardin* | XLOC-036073 | - | muscle | [2](https://app.readcube.com/library/7fac8260-73e5-4965-a22c-0410b504e320/all?uuid=2351808927340825&item_ids=7fac8260-73e5-4965-a22c-0410b504e320:8b219fe4-4be7-46c5-be56-a59daa2ac607) | 2 |
| *myoD* | XLOC-002761 | - | striated muscle | [2](https://app.readcube.com/library/7fac8260-73e5-4965-a22c-0410b504e320/all?uuid=37839080311212914&item_ids=7fac8260-73e5-4965-a22c-0410b504e320:8b219fe4-4be7-46c5-be56-a59daa2ac607) | 1 2 3 |
| *nk2.2* | XLOC-058223 | EF384210 | neuron | [7,10](https://app.readcube.com/library/7fac8260-73e5-4965-a22c-0410b504e320/all?uuid=6213143638686001&item_ids=7fac8260-73e5-4965-a22c-0410b504e320:6acd60cc-313c-41cd-9429-2fbda3a82222,7fac8260-73e5-4965-a22c-0410b504e320:e41d6f33-d347-4ae5-83ac-18ff195f32a1) | 4 11 20 |
| *nk6* | XLOC-053864 | EF384212 | neuron | [7,11](https://app.readcube.com/library/7fac8260-73e5-4965-a22c-0410b504e320/all?uuid=9283599424082105&item_ids=7fac8260-73e5-4965-a22c-0410b504e320:6acd60cc-313c-41cd-9429-2fbda3a82222,7fac8260-73e5-4965-a22c-0410b504e320:ecc341b9-f447-4a3f-ad05-462245081e75) | 5 11 |
| *sm-mrlc* | XLOC-001832 | - | smooth muscle | [2](https://app.readcube.com/library/7fac8260-73e5-4965-a22c-0410b504e320/all?uuid=2322560171571979&item_ids=7fac8260-73e5-4965-a22c-0410b504e320:8b219fe4-4be7-46c5-be56-a59daa2ac607) | 3 6 8 12 14 |
| *st-mrlc* | XLOC-052139 | - | striated muscle | [2](https://app.readcube.com/library/7fac8260-73e5-4965-a22c-0410b504e320/all?uuid=08701960189968916&item_ids=7fac8260-73e5-4965-a22c-0410b504e320:8b219fe4-4be7-46c5-be56-a59daa2ac607) | 3 6 8 12 14 |
| *synaptotagmin* | XLOC-000875 | EF544397 | neuron | [7](https://app.readcube.com/library/7fac8260-73e5-4965-a22c-0410b504e320/all?uuid=8540068756151856&item_ids=7fac8260-73e5-4965-a22c-0410b504e320:6acd60cc-313c-41cd-9429-2fbda3a82222) | 5 11 13 14 18 20 26 |
| *titin* | XLOC-008954 | - | striated muscle | [2](https://app.readcube.com/library/7fac8260-73e5-4965-a22c-0410b504e320/all?uuid=030189387130214573&item_ids=7fac8260-73e5-4965-a22c-0410b504e320:8b219fe4-4be7-46c5-be56-a59daa2ac607) | 2 10 17 |
| *troponin T* | XLOC-009874 | - | striated muscle | [2](https://app.readcube.com/library/7fac8260-73e5-4965-a22c-0410b504e320/all?uuid=5339902957295817&item_ids=7fac8260-73e5-4965-a22c-0410b504e320:8b219fe4-4be7-46c5-be56-a59daa2ac607) | 2 10 17 |
| *trph* | XLOC-037608 | EF544398 | neuron | [7](https://app.readcube.com/library/7fac8260-73e5-4965-a22c-0410b504e320/all?uuid=6615966969781777&item_ids=7fac8260-73e5-4965-a22c-0410b504e320:6acd60cc-313c-41cd-9429-2fbda3a82222) | 5 |
| *vtg* | XLOC-001283 | KU756287 | eleocyte | [12](https://app.readcube.com/library/7fac8260-73e5-4965-a22c-0410b504e320/all?uuid=4664883910162595&item_ids=7fac8260-73e5-4965-a22c-0410b504e320:658b18dd-6bdd-4a9b-b250-a8a930fa5060) | 7 |

**Table 1: Candidate marker genes used for cluster annotation, including their genome assembly ID (xloc), their genbank ID, their known expression pattern, relevant references, and the clusters most strongly expressing the respective gene.**
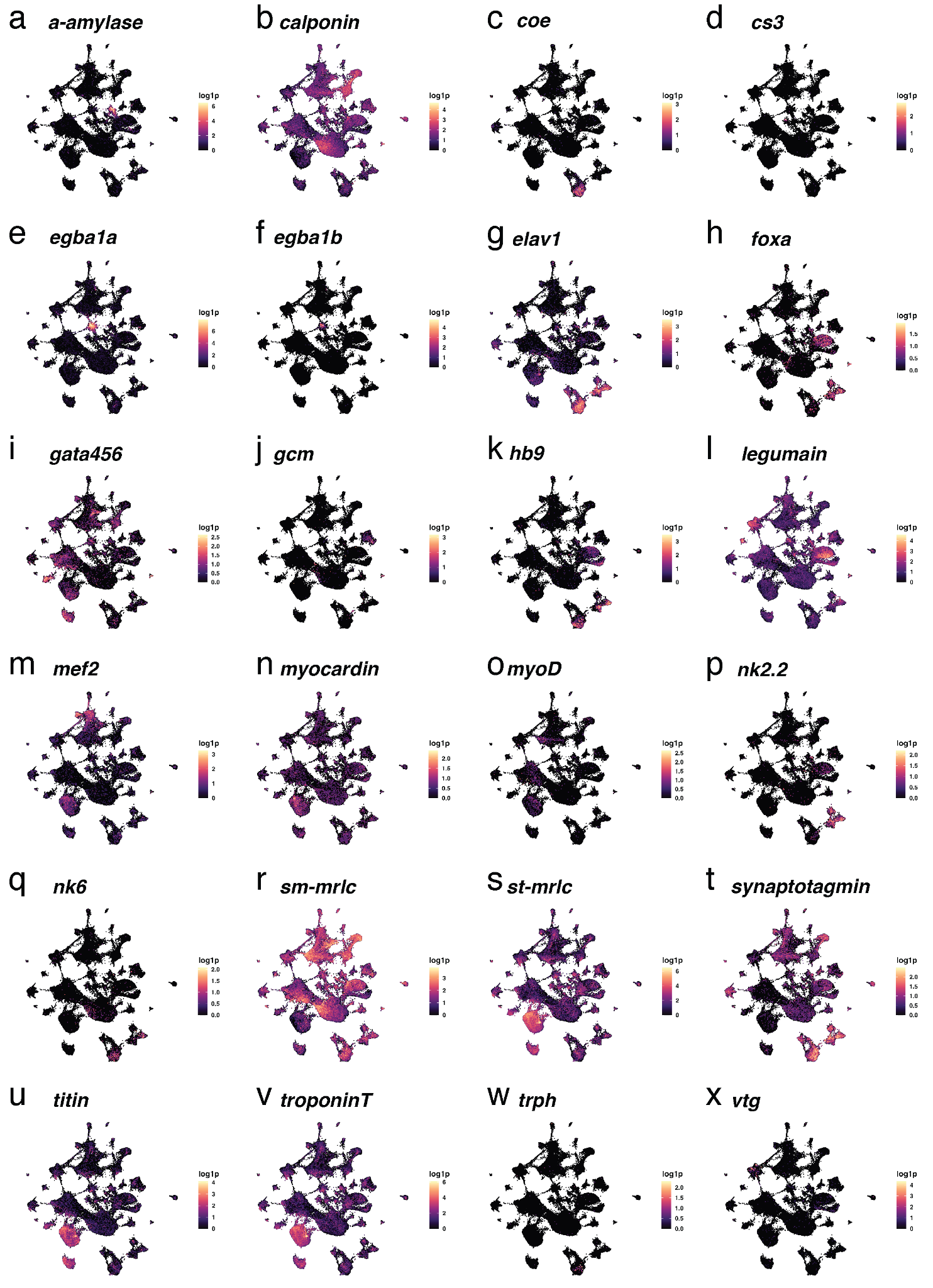


**Supplementary Data Figure 1: gene expression of candidate marker genes for cluster identification**(**a-x**) UMAP visualization of select marker genes used for cluster identification.

**Information on additional genes used in this manuscript**

| **gene** | **xloc** | **genbank** | **reference** |
| --- | --- | --- | --- |
| *cdx* | XLOC-038701 | DQ188196 | [13](https://app.readcube.com/library/7fac8260-73e5-4965-a22c-0410b504e320/all?uuid=1821163901485804&item_ids=7fac8260-73e5-4965-a22c-0410b504e320:f455afa9-22df-48b4-83b7-992d5cb41a4b) |
| *chd1* | XLOC-003547 | MW250936 | [14](https://app.readcube.com/library/7fac8260-73e5-4965-a22c-0410b504e320/all?uuid=8017339479708592&item_ids=7fac8260-73e5-4965-a22c-0410b504e320:743ea7fc-cc7b-43b4-a821-e9322689cc84) |
| *chd345b* | XLOC-050017 | MW250938 | [14](https://app.readcube.com/library/7fac8260-73e5-4965-a22c-0410b504e320/all?uuid=28933013936889074&item_ids=7fac8260-73e5-4965-a22c-0410b504e320:743ea7fc-cc7b-43b4-a821-e9322689cc84) |
| *cycb1* | XLOC-026926 | HE858370 | [9,15](https://app.readcube.com/library/7fac8260-73e5-4965-a22c-0410b504e320/all?uuid=20674902656152372&item_ids=7fac8260-73e5-4965-a22c-0410b504e320:0800d5e6-61c1-428b-91e5-3dc233b997c1,7fac8260-73e5-4965-a22c-0410b504e320:70e17732-1b8b-4d49-9d6a-f880f19a53b3) |
| *cycb3* | XLOC-024089 | HE858372 | [9,15](https://app.readcube.com/library/7fac8260-73e5-4965-a22c-0410b504e320/all?uuid=836883033056754&item_ids=7fac8260-73e5-4965-a22c-0410b504e320:0800d5e6-61c1-428b-91e5-3dc233b997c1,7fac8260-73e5-4965-a22c-0410b504e320:70e17732-1b8b-4d49-9d6a-f880f19a53b3) |
| *dlx* | XLOC-035503 | AM114774 | [16–18](https://app.readcube.com/library/7fac8260-73e5-4965-a22c-0410b504e320/all?uuid=525213196478726&item_ids=7fac8260-73e5-4965-a22c-0410b504e320:12bcb615-340c-45eb-9a56-d78dc7dcb1d7,7fac8260-73e5-4965-a22c-0410b504e320:74c47fff-76b9-42df-ae62-1e04e33739d4,7fac8260-73e5-4965-a22c-0410b504e320:136f4946-f58d-48ac-bf31-5f22c697896c) |
| *dnmt1* | XLOC-038208 | MW250929 | [14](https://app.readcube.com/library/7fac8260-73e5-4965-a22c-0410b504e320/all?uuid=9915303914940587&item_ids=7fac8260-73e5-4965-a22c-0410b504e320:743ea7fc-cc7b-43b4-a821-e9322689cc84) |
| *evx* | XLOC-043538 | DQ188195 | [9,13](https://app.readcube.com/library/7fac8260-73e5-4965-a22c-0410b504e320/all?uuid=6780673290955501&item_ids=7fac8260-73e5-4965-a22c-0410b504e320:f455afa9-22df-48b4-83b7-992d5cb41a4b,7fac8260-73e5-4965-a22c-0410b504e320:0800d5e6-61c1-428b-91e5-3dc233b997c1) |
| *four-jointed* | XLOC-007715 | HE858364 | [15](https://app.readcube.com/library/7fac8260-73e5-4965-a22c-0410b504e320/all?uuid=3800556673312401&item_ids=7fac8260-73e5-4965-a22c-0410b504e320:70e17732-1b8b-4d49-9d6a-f880f19a53b3) |
| *foxa* | XLOC-060337 | AM114771 | [8,16](https://app.readcube.com/library/7fac8260-73e5-4965-a22c-0410b504e320/all?uuid=3355417576062809&item_ids=7fac8260-73e5-4965-a22c-0410b504e320:12bcb615-340c-45eb-9a56-d78dc7dcb1d7,7fac8260-73e5-4965-a22c-0410b504e320:a6d140a3-0c8f-46da-bf99-5968d1174347) |
| *hdac3* | XLOC-004637 | MW250943 | [14](https://app.readcube.com/library/7fac8260-73e5-4965-a22c-0410b504e320/all?uuid=7885863791498119&item_ids=7fac8260-73e5-4965-a22c-0410b504e320:743ea7fc-cc7b-43b4-a821-e9322689cc84) |
| *hdac8* | XLOC-001297 | MW250944 | [14](https://app.readcube.com/library/7fac8260-73e5-4965-a22c-0410b504e320/all?uuid=46941218918059213&item_ids=7fac8260-73e5-4965-a22c-0410b504e320:743ea7fc-cc7b-43b4-a821-e9322689cc84) |
| *hox3* | XLOC-037912 | JQ424894 | [9,19](https://app.readcube.com/library/7fac8260-73e5-4965-a22c-0410b504e320/all?uuid=31300773758240863&item_ids=7fac8260-73e5-4965-a22c-0410b504e320:0800d5e6-61c1-428b-91e5-3dc233b997c1,7fac8260-73e5-4965-a22c-0410b504e320:17c1757f-b127-49bf-b98a-6e4915a9ff44) |
| *msx* | XLOC-050016 | CAJ38810 | [16,20](https://app.readcube.com/library/7fac8260-73e5-4965-a22c-0410b504e320/all?uuid=8300610753814844&item_ids=7fac8260-73e5-4965-a22c-0410b504e320:12bcb615-340c-45eb-9a56-d78dc7dcb1d7,7fac8260-73e5-4965-a22c-0410b504e320:517b95ec-84f0-4f69-924a-ae7f32138b36) |
| *myc* | XLOC-004670 | KC999055 | [9](https://app.readcube.com/library/7fac8260-73e5-4965-a22c-0410b504e320/all?uuid=7545678094549211&item_ids=7fac8260-73e5-4965-a22c-0410b504e320:0800d5e6-61c1-428b-91e5-3dc233b997c1) |
| *nanos* | XLOC-052426 | AM076486 | [9,21](https://app.readcube.com/library/7fac8260-73e5-4965-a22c-0410b504e320/all?uuid=867553158567238&item_ids=7fac8260-73e5-4965-a22c-0410b504e320:16c2d058-cf2f-40af-b802-42137e5adc43,7fac8260-73e5-4965-a22c-0410b504e320:0800d5e6-61c1-428b-91e5-3dc233b997c1) |
| *flrtl* | XLOC-008486 | PQ334001 | this study |
| *p2x* | XLOC-001439 | PQ334002 | this study |
| *pcna* | XLOC-025800 | HF935038 | [9](https://app.readcube.com/library/7fac8260-73e5-4965-a22c-0410b504e320/all?uuid=11909614362515542&item_ids=7fac8260-73e5-4965-a22c-0410b504e320:0800d5e6-61c1-428b-91e5-3dc233b997c1) |
| *piwi* | XLOC-026648 | AM076487 | [9,21](https://app.readcube.com/library/7fac8260-73e5-4965-a22c-0410b504e320/all?uuid=7076453281490624&item_ids=7fac8260-73e5-4965-a22c-0410b504e320:16c2d058-cf2f-40af-b802-42137e5adc43,7fac8260-73e5-4965-a22c-0410b504e320:0800d5e6-61c1-428b-91e5-3dc233b997c1) |
| *prrx* | XLOC-055252 | PQ334003 | this study |
| *runx* | XLOC-003787 | KC999057 | [9](https://app.readcube.com/library/7fac8260-73e5-4965-a22c-0410b504e320/all?uuid=19401809005324555&item_ids=7fac8260-73e5-4965-a22c-0410b504e320:0800d5e6-61c1-428b-91e5-3dc233b997c1) |
| *soxb1* | XLOC-064106 | ANS60443 | [11,22](https://app.readcube.com/library/7fac8260-73e5-4965-a22c-0410b504e320/all?uuid=3650209960356653&item_ids=7fac8260-73e5-4965-a22c-0410b504e320:1b42f00e-fd76-4de7-b4a3-e967460b3b06,7fac8260-73e5-4965-a22c-0410b504e320:ecc341b9-f447-4a3f-ad05-462245081e75) |
| *sp/btd* | XLOC-015251 | KU249168 | [17](https://app.readcube.com/library/7fac8260-73e5-4965-a22c-0410b504e320/all?uuid=9640869355110491&item_ids=7fac8260-73e5-4965-a22c-0410b504e320:74c47fff-76b9-42df-ae62-1e04e33739d4) |
| *mtor* | XLOC-043662 | PQ334004 | this study |
| *vasa* | XLOC-005003 | AM048812 | [9,21](https://app.readcube.com/library/7fac8260-73e5-4965-a22c-0410b504e320/all?uuid=049921908952613836&item_ids=7fac8260-73e5-4965-a22c-0410b504e320:16c2d058-cf2f-40af-b802-42137e5adc43,7fac8260-73e5-4965-a22c-0410b504e320:0800d5e6-61c1-428b-91e5-3dc233b997c1) |
| *epig1* | XLOC-049416 | PQ333993 | this study |
| *ccdc134-like* | XLOC-046912 | PQ333994 | this study |
| *col6a6* | XLOC-015056 | PQ333995 | this study |
| *lamtor1* | XLOC-020058 | PQ333996 | this study |
| *lamtor2* | XLOC-009167 | PQ333997 | this study |
| *lamtor3* | XLOC-025033 | PQ333998 | this study |
| *lamtor4* | XLOC-024406 | PQ333999 | this study |
| *lamtor5* | XLOC-013219 | PQ334000 | this study |
| *smg1* | XLOC-028373 | PQ334005 | this study |

**Table 2: additional marker genes used for this manuscript, including their genome assembly ID (xloc), their genbank ID, and relevant references.**

**Extended references for Supplementary Data 2:**

[1. Williams, E. A., Conzelmann, M. & Jékely, G. Myoinhibitory peptide regulates feeding in the marine annelid Platynereis. *Front. Zoöl.* **12**, 1 (2015).
2. Brunet, T. *et al.* The evolutionary origin of bilaterian smooth and striated myocytes. *eLife* **5**, e19607 (2016).
3. Demilly, A. et al. Coe Genes Are Expressed in Differentiating Neurons in the Central Nervous System of Protostomes. *PLoS ONE* **6**, e21213 (2011).
4. Zakrzewski, A. Molecular characterization of chaetae formation in annelida and other Lophotrochozoa. (Freie Universität Berlin, Germany, 2011).
5. Zakrzewski, A.-C. et al. Early Divergence, Broad Distribution, and High Diversity of Animal Chitin Synthases. *Genome Biol. Evol.* **6**, 316–325 (2014).
6. Song, S. et al. Globins in the marine annelid Platynereis dumerilii shed new light on hemoglobin evolution in bilaterians. *BMC Evol. Biol.* **20**, 165 (2020).
7. Denes, A. S. et al. Molecular Architecture of Annelid Nerve Cord Supports Common Origin of Nervous System Centralization in Bilateria. *Cell* **129**, 277–288 (2007).
8. Kostyuchenko, R. P., Kozin, V. V., Filippova, N. A. & Sorokina, E. V. FoxA expression pattern in two polychaete species, Alitta virens and Platynereis dumerilii: Examination of the conserved key regulator of the gut development from cleavage through larval life, postlarval growth, and regeneration. *Dev. Dyn.* **248**, 728–743 (2019).
9. Gazave, E. et al. Posterior elongation in the annelid Platynereis dumerilii involves stem cells molecularly related to primordial germ cells. *Dev. Biol.* **382**, 246–267 (2013).
10. Gazave, E., Lemaître, Q. I. B. & Balavoine, G. The Notch pathway in the annelid Platynereis: insights into chaetogenesis and neurogenesis processes. *Open Biol.* **7**, 160242 (2017).
11. Vergara, H. M. et al. Whole-organism cellular gene-expression atlas reveals conserved cell types in the ventral nerve cord of Platynereis dumerilii. *Proc. Natl. Acad. Sci.* **114**, 5878–5885 (2017).
12. Schenk, S., Krauditsch, C., Frühauf, P., Gerner, C. & Raible, F. Discovery of methylfarnesoate as the annelid brain hormone reveals an ancient role of sesquiterpenoids in reproduction. eLife 5, e17126 (2016).
13. Rosa, R., Prud’homme, B. & Balavoine, G. caudal and even‐skipped in the annelid Platynereis dumerilii and the ancestry of posterior growth. *Evol. Dev.* **7**, 574–587 (2005).
14. Planques, A. et al. DNA methylation atlas and machinery in the developing and regenerating annelid Platynereis dumerilii. *BMC Biol.* **19**, 148 (2021).
15. Demilly, A., Steinmetz, P., Gazave, E., Marchand, L. & Vervoort, M. Involvement of the Wnt/β-catenin pathway in neurectoderm architecture in Platynereis dumerilii. *Nat. Commun.* **4**, 1915 (2013).
16. Raible, F. et al. Vertebrate-Type Intron-Rich Genes in the Marine Annelid Platynereis dumerilii. *Science* **310**, 1325–1326 (2005).
17. Grimmel, J., Dorresteijn, A. W. C. & Fröbius, A. C. Formation of body appendages during caudal regeneration in Platynereis dumerilii: adaptation of conserved molecular toolsets. *EvoDevo* **7**, 10 (2016).
18. Planques, A., Malem, J., Parapar, J., Vervoort, M. & Gazave, E. Morphological, cellular and molecular characterization of posterior regeneration in the marine annelid Platynereis dumerilii. Dev. Biol. **445**, 189–210 (2019).
19. Pfeifer, K., Dorresteijn, A. W. C. & Fröbius, A. C. Activation of Hox genes during caudal regeneration of the polychaete annelid Platynereis dumerilii. *Dev. Genes Evol.* **222**, 165–179 (2012).
20. Saudemont, A. et al. Complementary striped expression patterns of NK homeobox genes during segment formation in the annelid Platynereis. Dev. Biol. **317**, 430–443 (2008).
21. Rebscher, N., Zelada-González, F., Banisch, T. U., Raible, F. & Arendt, D. Vasa unveils a common origin of germ cells and of somatic stem cells from the posterior growth zone in the polychaete Platynereis dumerilii. Dev. Biol. **306**, 599–6**11** (2007).
22. Kerner, P., Simionato, E., Gouar, M. L. & Vervoort, M. Orthologs of key vertebrate neural genes are expressed during neurogenesis in the annelid Platynereis dumerilii. Evol. Dev. 11, 513–524 (2009).](https://app.readcube.com/library/?style=Nature%20Communications+%7B%22language%22:%22en-US%22%7D)
