# Supplementary material for: Molecular profiles, sources and lineage restrictions of stem cells in an annelid regeneration model
Source: Nat Commun. 2024 Nov 18;15:9882. doi: 10.1038/s41467-024-54041-3 (PMC11574210; doi:10.1038/s41467-024-54041-3)
Supplement: Supplementary file 8 — Supplementary Data 5 [file 41467_2024_54041_MOESM8_ESM.docx]

# Supplementary data 4:

**HCR probe target templates and probe sequences**

Gene: *Platynereis* *epig1*

Matching ID in draft genome annotation: XLOC-049416

CCAACATTTGCATCATATTGGCAGAATTATTCTAAATTATATCCTATTATATCTAGCAACAAATTTGCTGTGTCAAATAATTGTTGTGCTGATTGCAAGGCATTGAGGGCAACCACAAAGAAAAGAAGAGTGTGACACTGAGTCACAATGTTTGCTGACATGGCTCGATGTATGAGCGGCATAAACAGGGTGAATGCTCAAAGCAAAAATTATATTTTTGTCCAACAACAAACACATTGGTTCCGGATCGCCACCCTCTGTTGCTTGCAAAATCACTTTAGAGAAGATCCAACACCCACTTAAAACAGATCACAGCACACCAGCAGAATGAACACAACAATCCTACTTTGTTTTGCTATCTCATTTATAGCTATAACCCATGGCAGCAAAATTGAAGACCTGCTAAAAGAACTGAAAAGGAAGACTTTGGAAAAGAGGCAAGAGCCTAAACAGCCGGAAGAGGCATGTGATGCAACTGCGGCCTTCGTGAAATGTCATAAAAAAGCTAACATGAAACTTCCAGACCAGACTGAATATGACCTCAGCAATGCAGCAAGCTTGGCAAAATATCAAAAGGAGATTATGAGGATTAACGACCCAGTGACCATGTTGACATTTGGAAAGTGCTGGCAAGAAGAATGTCCAGTTGACTCAAGTGATGAAGAGCCTGAAGGTTGTGACTCCGACCAAACTTTCTATGAATGTCAAAAACAAGCAAAATTGACACTTCCGAGCTTGAGCAAATATGACTGGAAGAACGAGAAAGCTGAGGATCAATTCTTTTGCGATATTGTGAAAACGAATGGAAAGGGTAAAATGGATAACTTCAAAAAGTGCTGGATGAGCAAATGTCCGGGAGAAACAGAATTGGACCTGCCAAAATGTGAAAACCAAGCACCTGGAAACAAGCAAGACCAGTCCCTGCTGTTGAAGAAACTCCTCCAGTTGATTGATTCGGCCTAACTCCTTAAGGAGACTGGAGACAGAAAATACTGATTGAAAATCAGACTTATGACACACACAGATTTGACTGCTTTTTAGAAATACTTTTAAAAGTACATAGCATATGAGACCTCTCATGCATATTTGTCTCGCACTGGAAAAAACGTTTGTGTGAAACAGACAAAACAAACATTAGCTGGTGTCCATCCGAAAATTTTTGTAAATTAAGACAAAAG

HCR Probes (B1 adapter):

GAGGAGGGCAGCAAACGGaaGGATGGACACCAGCTAATGTTTGTT

CTTTTGTCTTAATTTACAAAAATTTtaGAAGAGTCTTCCTTTACG

GAGGAGGGCAGCAAACGGaaTGCGAGACAAATATGCATGAGAGGT

TCTGTTTCACACAAACGTTTTTTCCtaGAAGAGTCTTCCTTTACG

GAGGAGGGCAGCAAACGGaaCTAAAAAGCAGTCAAATCTGTGTGT

ATATGCTATGTACTTTTAAAAGTATtaGAAGAGTCTTCCTTTACG

GAGGAGGGCAGCAAACGGaaCTGTCTCCAGTCTCCTTAAGGAGTT

ATAAGTCTGATTTTCAATCAGTATTtaGAAGAGTCTTCCTTTACG

GAGGAGGGCAGCAAACGGaaTCAACAGCAGGGACTGGTCTTGCTT

CCGAATCAATCAACTGGAGGAGTTTtaGAAGAGTCTTCCTTTACG

GAGGAGGGCAGCAAACGGaaCAGGTCCAATTCTGTTTCTCCCGGA

TCCAGGTGCTTGGTTTTCACATTTTtaGAAGAGTCTTCCTTTACG

GAGGAGGGCAGCAAACGGaaTCCATTTTACCCTTTCCATTCGTTT

TTGCTCATCCAGCACTTTTTGAAGTtaGAAGAGTCTTCCTTTACG

GAGGAGGGCAGCAAACGGaaCTTTCTCGTTCTTCCAGTCATATTT

CAATATCGCAAAAGAATTGATCCTCtaGAAGAGTCTTCCTTTACG

GAGGAGGGCAGCAAACGGaaTTTTTGACATTCATAGAAAGTTTGG

CAAGCTCGGAAGTGTCAATTTTGCTtaGAAGAGTCTTCCTTTACG

GAGGAGGGCAGCAAACGGaaCTTGAGTCAACTGGACATTCTTCTT

GAGTCACAACCTTCAGGCTCTTCATtaGAAGAGTCTTCCTTTACG

GAGGAGGGCAGCAAACGGaaCTGGGTCGTTAATCCTCATAATCTC

AGCACTTTCCAAATGTCAACATGGTtaGAAGAGTCTTCCTTTACG

GAGGAGGGCAGCAAACGGaaGCTGAGGTCATATTCAGTCTGGTCT

TTGATATTTTGCCAAGCTTGCTGCAtaGAAGAGTCTTCCTTTACG

GAGGAGGGCAGCAAACGGaaTTCACGAAGGCCGCAGTTGCATCAC

AGTTTCATGTTAGCTTTTTTATGACtaGAAGAGTCTTCCTTTACG

GAGGAGGGCAGCAAACGGaaTCTTTTCCAAAGTCTTCCTTTTCAG

CCTCTTCCGGCTGTTTAGGCTCTTGtaGAAGAGTCTTCCTTTACG

GAGGAGGGCAGCAAACGGaaATGGGTTATAGCTATAAATGAGATA

TTTTAGCAGGTCTTCAATTTTGCTGtaGAAGAGTCTTCCTTTACG

GAGGAGGGCAGCAAACGGaaCTGCTGGTGTGCTGTGATCTGTTTT

AAACAAAGTAGGATTGTTGTGTTCAtaGAAGAGTCTTCCTTTACG

GAGGAGGGCAGCAAACGGaaTTTGCAAGCAACAGAGGGTGGCGAT

TGGGTGTTGGATCTTCTCTAAAGTGtaGAAGAGTCTTCCTTTACG

GAGGAGGGCAGCAAACGGaaAATATAATTTTTGCTTTGAGCATTC

GAACCAATGTGTTTGTTGTTGGACAtaGAAGAGTCTTCCTTTACG

GAGGAGGGCAGCAAACGGaaATGTCAGCAAACATTGTGACTCAGT

CTGTTTATGCCGCTCATACATCGAGtaGAAGAGTCTTCCTTTACG

GAGGAGGGCAGCAAACGGaaTCAATGCCTTGCAATCAGCACAACA

ACACTCTTCTTTTCTTTGTGGTTGCtaGAAGAGTCTTCCTTTACG

Gene: *Platynereis* *ccdc134-like*

Matching ID in draft genome annotation: XLOC-046912

CCCCTTTCCTTTAAGTCCCAGACTTCCACTTGTAACTAAGAGAGAGTGTTCCCTGGCAACCGCAAATTGCTATCAGCGCTTCTGCTTTGTACAAGGAGGGACCACAATGTTCACAAGTGTCGCCATGGCAACCACTGTTGTATTGTTCACAGTTTTACAACTGTTGCTACAAGCCTGCACTGCATTTGTACTGTTGCCTAGCAACAACCCTGGCAACAGTAGCAAGAGCATTAGCAACAGCACGATTAATGATGACATCACAAAGGCAAACGCAACCATCACCATGACGAATCACACCAAGAGCACAGAAAATGATGATGAGACCAAAATGCAGAGAACAAACGACACACGAGCATTCAGAGAACAGATGGTCCTGCGACACTTGGCGGAAAATAGGAGAGACCAACCGTGCTTCCAAGCTCGTCAGAGTTACGACCGTCTCATAGAAACAACCGGACCTATCCCTGGTTACGACCGGCCAGTCTGTTATGAAGATGGGACGTATATGCCAAAACAGTGCAAGGGAAATATTTGTTACTGCATGGCACCAGATGGCACCAGGTTAGGAAGATACAGACAGCAGCGCCACCGCGCTAAATACATGCATTGTTTGTGTGCACTGGACCAATACGGCCTCTCACTACTAGGACTGAGAGACACGTTTGCTTGCGCCAACAACGGTAACTACCGACGAATCCAATGCCACCGCTCAACCTGCTTCTGCACGGATCACCATGGCAACGTCCTAGAACATATCGACCCCGTCCATATTGATGATAAACAAAAATTGAACTGTTTGCAACGCACAAAAAGTAACACTAGATCTCAACATGATTCAGTCGAATAAAAAATCGATTAACAAATTAGTTTATATATGAAATTTTATATGAAATTCAAAATATCGATCTGTTATAACATGACATCTTACCAATTTATGGGAAAAATTCTTAAGTGATATAAATTAAATTCATTTGTATGAAATGTATGTGGTTAAAATTTTTTAAAATCAAATTCACAAAAAAACAACCTATTACATGAAATTGTTTATTTGGTATTTAAAAAGGGGAAAAACTGTCATATTCTGAATTATAACAATGTTCAATTTCGTTAACGGTCCTGAGTTTTAAAAGTTACTAATTCTCAAGATAGTACTGTGATGATTATGATACGGTGCTAATGAAATAAAGGGTGTCTTAATGTGTAAAAAAAAAAAAAAAAAAAAGTTAAGATAAAAATTTATTTAACTTGGGTGATGAAAATAAAAAAAAGAAAAATAAGATACAAAGCTTTTAA

HCR Probes (B2 adapter):

CCTCGTAAATCCTCATCAaaTCATTAGCACCGTATCATAATCATC

TTTTTACACATTAAGACACCCTTTAaaATCATCCAGTAAACCGCC

CCTCGTAAATCCTCATCAaaAAAACTCAGGACCGTTAACGAAATT

GTACTATCTTGAGAATTAGTAACTTaaATCATCCAGTAAACCGCC

CCTCGTAAATCCTCATCAaaTTTTCCCCTTTTTAAATACCAAATA

CATTGTTATAATTCAGAATATGACAaaATCATCCAGTAAACCGCC

CCTCGTAAATCCTCATCAaaCAAATGAATTTAATTTATATCACTT

AAAAATTTTAACCACATACATTTCAaaATCATCCAGTAAACCGCC

CCTCGTAAATCCTCATCAaaCATGTTATAACAGATCGATATTTTG

AATTTTTCCCATAAATTGGTAAGATaaATCATCCAGTAAACCGCC

CCTCGTAAATCCTCATCAaaTCTAGTGTTACTTTTTGTGCGTTGC

TTTTTATTCGACTGAATCATGTTGAaaATCATCCAGTAAACCGCC

CCTCGTAAATCCTCATCAaaTGGACGGGGTCGATATGTTCTAGGA

CAGTTCAATTTTTGTTTATCATCAAaaATCATCCAGTAAACCGCC

CCTCGTAAATCCTCATCAaaTTGAGCGGTGGCATTGGATTCGTCG

TGCCATGGTGATCCGTGCAGAAGCAaaATCATCCAGTAAACCGCC

CCTCGTAAATCCTCATCAaaGTCTCTCAGTCCTAGTAGTGAGAGG

GTTACCGTTGTTGGCGCAAGCAAACaaATCATCCAGTAAACCGCC

CCTCGTAAATCCTCATCAaaATGTATTTAGCGCGGTGGCGCTGCT

TATTGGTCCAGTGCACACAAACAATaaATCATCCAGTAAACCGCC

CCTCGTAAATCCTCATCAaaGTGCCATGCAGTAACAAATATTTCC

TGTATCTTCCTAACCTGGTGCCATCaaATCATCCAGTAAACCGCC

CCTCGTAAATCCTCATCAaaTTCATAACAGACTGGCCGGTCGTAA

GCACTGTTTTGGCATATACGTCCCAaaATCATCCAGTAAACCGCC

CCTCGTAAATCCTCATCAaaCGGTCGTAACTCTGACGAGCTTGGA

GGGATAGGTCCGGTTGTTTCTATGAaaATCATCCAGTAAACCGCC

CCTCGTAAATCCTCATCAaaAGTGTCGCAGGACCATCTGTTCTCT

ACGGTTGGTCTCTCCTATTTTCCGCaaATCATCCAGTAAACCGCC

CCTCGTAAATCCTCATCAaaTTTGGTCTCATCATCATTTTCTGTG

TGCTCGTGTGTCGTTTGTTCTCTGCaaATCATCCAGTAAACCGCC

CCTCGTAAATCCTCATCAaaGCGTTTGCCTTTGTGATGTCATCAT

TTGGTGTGATTCGTCATGGTGATGGaaATCATCCAGTAAACCGCC

CCTCGTAAATCCTCATCAaaTGTTGCCAGGGTTGTTGCTAGGCAA

TCGTGCTGTTGCTAATGCTCTTGCTaaATCATCCAGTAAACCGCC

CCTCGTAAATCCTCATCAaaCAGTTGTAAAACTGTGAACAATACA

TACAAATGCAGTGCAGGCTTGTAGCaaATCATCCAGTAAACCGCC

CCTCGTAAATCCTCATCAaaATTGTGGTCCCTCCTTGTACAAAGC

GTGGTTGCCATGGCGACACTTGTGAaaATCATCCAGTAAACCGCC

CCTCGTAAATCCTCATCAaaGGGAACACTCTCTCTTAGTTACAAG

AGCGCTGATAGCAATTTGCGGTTGCaaATCATCCAGTAAACCGCC

Gene: *Platynereis flrtl*

Matching ID in draft genome annotation: XLOC-008486

ATGTTGGTGCTTGGACTACCAGACTGTTCCCTTGCAGTCACTTTGTTATTTTTATTGACTGTCACAGTGCATGGACAATGTCCAGTATCCAGACGTTCCATGGCCTACTGCAGCTGCAACTACAGGGATAACAATGGCTACAGAGTGGCTCGTTCTATCGTTTGCAGTGGACTTAATCCTAATACGACCCTCATATTCACTGACACGAGGGACATTTTCGTGGATTTAATCATCAGGAATTCTACCATGGACCAAGTAATCCCATTTATGTTTTCGAGGGTCAAAGCAAGAAACTTCATCATGACCCATAACGAGATCCTAAGTATAGATGACAATGCCTTTCTCGGAGCTGAGGATTACATAGAAAGCCTGGATTTATCTCATAATCTGATTACCTCTTTGCCTCCTGCCATCTTCAAGTTGCTGCGATTCCTGAAGATCCTTAATCTGTCCAACAACAGACTGACCACGATTTACAAATCTGCTTTCGAAGGTTTGTCCAGTTTAACTTACCTCAACCTGGGTAGGAATAACATCTATGACATCAGAAGTGATGGTTTCTCTGAACTGGTTAATGTGAAGACTTTGTTGTTGGATGGAAATGCCATTGGTGAGCTGAAAAATGACTCTTTCTCTGGTCTGAGCAGGTTGCAACTCCTGCATCTTTCGTCCAACAAGATCAGCAAAATCTCAGGAGATGCATTCGCACCATTGACCAGTCTCCGCTCCCTCTACCTCCCTCTGAACGACTTGCCCTCCATCCCGGAGAGCATGCTCAGGATGAACGAATACCTGGAAGAACTTTCGCTCGCCGACAACGAACTCACGAGTATTCCTGAAAATGTGTTCTTCCTCAACAAATATCTAAAGTCAATAGATATCTCATTCAACAAATTAAATACGATTGAGAAGTTTCCTTTCAAAAAACTTGAACGCCTAGAAAAACTCAATTTACAAAACAATAAGATTAGATCAATATACAAAAACGTCTTTGAAAACTTAAACCAAGTGCAAACACTGAACTTGGCAAACAATCTAATTGACAACCTGGAAGAAAACGGGCTTGCTGGATTGGCTGTGACCCAGACAGTTAGATTAGACAACAACTTGATATCTATTGTCCCATATGGAGTGTTTGATGCAATAGGAAATGTGGTTTATTTGAACCTTTCAATGAATCTAATTGAAAACTTTGAGGACAGCAATCCATTCCTGGCTAATAATGTTTTAAAGGAGCTTGATTTAAGCTGGAATCGGATCAAAGAGGTTCCACCTACTTTATTTAAAAAGACCAGATCTCTCCAGAAACTCAGTATGGCTCACAATGAAATTACCATGATGGATCCAGAGAGTTTTATCAATGTACCCAAGTTAGAATCACTAGACTTGACACACAATCGGATAAACTCACCGGATGGGAACCTTTTCTACAATTGTCCTAAGCTCAAGACGCTGAAACTCTCGTGGAATCCTTTGCAAGAGCTTCCGCGGTCCTTCTTAGTCGGCCTCAGTGGCTTGGAAGAAGTGAATTTGGACCACGGATGCTTAACGGGTCTCCCAGATGGCTTCTTCACTCCAGTCGATGATACGGTCAATGCCACAACTCCTCCTGAACTTCAGCAGCCCGTTCCTCTTACGAACCTCAAAGTTGCTCATTTGAACAGTAACTTCCTCCAGGGCATCAACTCCACACAACTCCAGGGGCCAAAGTTGTCCACATTGAACATTCAGGAGAACAACATCTCCATGATAGACCCTGGGGTGTTCTCCTTATTCTCCACATTGGAGTCTGCGAACCTCTCGTGGAATGACCTAACATCATCCATCAGAGATTCATTTGGCAACATGCGCACTGTGAAAGTATTGGACTTGAGACATAACCAGATAGAAGAGTTGAACAGGAGAGCCCTTGAGGTTGAAACTGCTTTTGTGGAACTTTACTTGGAAGGGAACCCTATCTATTGTGATTGTGATATGGCTTTCCTCCGTGTGTATGATAATCTAAAGGACCCGGACTCATTGCTGTGTGGTTCCAGTAATACTGGATTAGATGAAGAATTAGTCATCTGCACTTCAACTCAGTCCCTCGGTTGCTCTAGTTGGCCGAGTAATCATCGAACTCTGTGTGCCAGAACGACACAAAATGACCTTGAGGCCAAACGTGACCAGCTGATTCAAGCTGCTGAAGACTACCAACAAGAAAGAATGGATTATGAGGAGTTCTGTGTAATTTATGAGGGAATGACGACAAGCGGGTATACCTTGTTAGATCCAAATTTTACCAACTTCTCTAACATTGATCTACCAGAGATCACCAAAATCACACCAACGCCTGACGATAATTATATCCGGGTTGAGTGGAAGCATGAGGATATAAGCCTTGTGTCGGGATACAGACTGAGGTACACAAATTCATCAAACAATGTGACACACACCAGTGAAGTGGTGGACGACCGCCTGTGGTATGTCCTGAACAACACTCACATTGATGAGAATTACCTCATCTGTGTGGAGGTGATCCTTTTGGATGGGCAGTCGAGGTTGGATGAGAGGAGATGCAGGGAAGTGAGAGATGTGCTGGTCAGGAAGGTCAGTGAAGAGGATGACCGCTCTGGGCTGCTGCAACTGCTCGTGCCCATCATCGCTGTAGCAGCGGTCCTCCTCATCATCATCCTCATCATCATCGGTGTCCGCGTCAGTCTGAGGAGGAAGCGTCATCGTGACAAGCAGCAGGAAGCCATGACAGCTGAACAAAAACTGGGCATGTGGGACTTTCCCTTCAAACCACATGCAAGTGAAGAACTGGTGGAGCAGACCACCTTGCCTTATGACGTCTACTCTGAGACTATCGTTGATCCCAAATTCCGTCTGAAGAAGTTGAGGGCGACAATCAGAGGCATGGGAAAGACAAAACGCGTCAAGAGGAGGAAGTCACAGAATAGACAGATTCCGTCCGTGGCTGGGTCCATGAGATCTATTAACTCCATGAAGAGCGATTCCATGAACAGCATCCATTCCAATAACAGCCTGGATGATGTTGACACGGGACTCTTTATTTCGTCACAGGGCGAGATCCTCCCCCCTCCTTACACAGAGTCTGCCAATGGATCTATTGCGAATGGATCTGTTGCGAATGGATCTGTCGCAAACGGTTCTGTTGCGAACGGTTCTTTAACCAGCATTTCCATTATGGGAAATGGCAGTGTGAAAAGCAGTGTGAAAAGTGGTGCTAACAGTATGAGAAGTGTGAAGAACAGTGCCAGGGGCAGCAGAGGAAGTGCCAGAAACAGTAGAAACAGTAAAAACAGTAGAAAAGGCAAGAGGAACAGTGGTTATGAGAGTGACAATAGAAGTGGAGAGTGA

HCR probes (B2 adapter):

CCTCGTAAATCCTCATCAaaATAACCACTGTTCCTCTTGCCTTTT

TCACTCTCCACTTCTATTGTCACTCaaATCATCCAGTAAACCGCC

CCTCGTAAATCCTCATCAaaGCACTTCCTCTGCTGCCCCTGGCAC

CTGTTTTTACTGTTTCTACTGTTTCaaATCATCCAGTAAACCGCC

CCTCGTAAATCCTCATCAaaCACTTTTCACACTGCTTTTCACACT

TCTTCACACTTCTCATACTGTTAGCaaATCATCCAGTAAACCGCC

CCTCGTAAATCCTCATCAaaAGAACCGTTCGCAACAGAACCGTTT

ATTTCCCATAATGGAAATGCTGGTTaaATCATCCAGTAAACCGCC

CCTCGTAAATCCTCATCAaaGCAATAGATCCATTGGCAGACTCTG

ACAGATCCATTCGCAACAGATCCATaaATCATCCAGTAAACCGCC

CCTCGTAAATCCTCATCAaaTAAAGAGTCCCGTGTCAACATCATC

GGGGGAGGATCTCGCCCTGTGACGAaaATCATCCAGTAAACCGCC

CCTCGTAAATCCTCATCAaaGGAATCGCTCTTCATGGAGTTAATA

GCTGTTATTGGAATGGATGCTGTTCaaATCATCCAGTAAACCGCC

CCTCGTAAATCCTCATCAaaTGTCTATTCTGTGACTTCCTCCTCT

CTCATGGACCCAGCCACGGACGGAAaaATCATCCAGTAAACCGCC

CCTCGTAAATCCTCATCAaaTTGTCGCCCTCAACTTCTTCAGACG

CGCGTTTTGTCTTTCCCATGCCTCTaaATCATCCAGTAAACCGCC

CCTCGTAAATCCTCATCAaaGACGTCATAAGGCAAGGTGGTCTGC

TTTGGGATCAACGATAGTCTCAGAGaaATCATCCAGTAAACCGCC

CCTCGTAAATCCTCATCAaaAAGGGAAAGTCCCACATGCCCAGTT

ACCAGTTCTTCACTTGCATGTGGTTaaATCATCCAGTAAACCGCC

CCTCGTAAATCCTCATCAaaTGTCACGATGACGCTTCCTCCTCAG

GTTCAGCTGTCATGGCTTCCTGCTGaaATCATCCAGTAAACCGCC

CCTCGTAAATCCTCATCAaaGATGATGAGGAGGACCGCTGCTACA

GACGCGGACACCGATGATGATGAGGaaATCATCCAGTAAACCGCC

CCTCGTAAATCCTCATCAaaCCAGAGCGGTCATCCTCTTCACTGA

ATGATGGGCACGAGCAGTTGCAGCAaaATCATCCAGTAAACCGCC

CCTCGTAAATCCTCATCAaaTGCATCTCCTCTCATCCAACCTCGA

TCCTGACCAGCACATCTCTCACTTCaaATCATCCAGTAAACCGCC

CCTCGTAAATCCTCATCAaaGATGAGGTAATTCTCATCAATGTGA

CCCATCCAAAAGGATCACCTCCACAaaATCATCCAGTAAACCGCC

CCTCGTAAATCCTCATCAaaTCCACCACTTCACTGGTGTGTGTCA

TTGTTCAGGACATACCACAGGCGGTaaATCATCCAGTAAACCGCC

CCTCGTAAATCCTCATCAaaTGTATCCCGACACAAGGCTTATATC

TGTTTGATGAATTTGTGTACCTCAGaaATCATCCAGTAAACCGCC

CCTCGTAAATCCTCATCAaaATCGTCAGGCGTTGGTGTGATTTTG

ATGCTTCCACTCAACCCGGATATAAaaATCATCCAGTAAACCGCC

CCTCGTAAATCCTCATCAaaTTGGTAAAATTTGGATCTAACAAGG

ATCTCTGGTAGATCAATGTTAGAGAaaATCATCCAGTAAACCGCC

Gene: *Platynereis prrx*

Matching ID in draft genome annotation: XLOC-055252

ATGAGTAGCTACGGAATTGCCTCAGCTTACTACTCTCCTGCAGCGGCATACTACGACCACAAGTTCCAACATTACAACGCCCTCAACAAACCCAACTTCTCGGTGAGCCACTTGCTGGACTTGGAAGAACTACCAAGAGAGAACTGCGCCATGTTCGCCAACACAGACATGACAGGCAAGCCTCACTCCGGGGGCTCGGCCCAGGGCATGTTGCTGCACACGGTGCCCGACAGGATCCCCTCCGAAAGGATTCCCTCTGACAGGATCCCCTCCGACAGGATCCCGTCCGACAGGATGGCCGATAGACTGGACAGGCCCAGCCCGGCCTCCCCTCCCCTCTCCCCAAACAAACTCATGAACAACAACAACTCGTCCAATCCCAGCAACCTGTCTTCTTCCTGCATCCACATTAAACCCTCCAGCCTCAGTCCGGACCTCGACAAGAAGTCAGAGGACGGCAAAGACTCAGAGTCAGAGGGCAAGGAGGAGGGCGGAGGCAAGGGGGGCAAGCGGAAGGCCCGTAGGAACAGGACGACATTCACCAGCACGCAACTGGCCGCCCTCGAGAGGGTCTTCGAGAGGACCCACTACCCGGACGCCTTCGTGAGGGAGGAGCTCGCCAGGAGGGTCAGCCTCAGCGAGGCCAGGGTCCAGGTTTGGTTCCAAAACAGAAGGGCAAAATTCCGACGCAACGAACGTAACATGCTTGCCCAGAGGTCATCACTGTACGGCAGTCCGCGGATGGAGTCTTCAGGTCCCCTGGAGCAACCCATTGGCCCACGACCCACCTCCCTCAGTCCCGAGTACTTGGGCTGGCCCGGGGCAGGGGGCACTTACAGCCCAGTGGCCAGCTCTCCTGGCTACAACATGTCTCCCGCCGGAATGGGCACCTCGACCACCTCTTCCTCCAGCTGTGCGTACGCTGCTCAAGGTGTGTACTCGGCGGCACCTCCTTCGGTGGGGTCGAGCATTGCCACTCTAAGACTGAAAGCGCGTGAATATAATATGCAGCAACAATCCCACCACTACATGCCTCCCCACCACCACCAGATGGCTCAGTGA

HCR probes (B2 adapter):

CCTCGTAAATCCTCATCAaaAGGCATGTAGTGGTGGGATTGTTGC

TCACTGAGCCATCTGGTGGTGGTGGaaATCATCCAGTAAACCGCC

CCTCGTAAATCCTCATCAaaAGAGTGGCAATGCTCGACCCCACCG

ATATTATATTCACGCGCTTTCAGTCaaATCATCCAGTAAACCGCC

CCTCGTAAATCCTCATCAaaCAGCGTACGCACAGCTGGAGGAAGA

GAGGTGCCGCCGAGTACACACCTTGaaATCATCCAGTAAACCGCC

CCTCGTAAATCCTCATCAaaCATGTTGTAGCCAGGAGAGCTGGCC

GGTCGAGGTGCCCATTCCGGCGGGAaaATCATCCAGTAAACCGCC

CCTCGTAAATCCTCATCAaaCAGCCCAAGTACTCGGGACTGAGGG

GGGCTGTAAGTGCCCCCTGCCCCGGaaATCATCCAGTAAACCGCC

CCTCGTAAATCCTCATCAaaGGGGACCTGAAGACTCCATCCGCGG

TGGGTCGTGGGCCAATGGGTTGCTCaaATCATCCAGTAAACCGCC

CCTCGTAAATCCTCATCAaaCATGTTACGTTCGTTGCGTCGGAAT

GCCGTACAGTGATGACCTCTGGGCAaaATCATCCAGTAAACCGCC

CCTCGTAAATCCTCATCAaaACCCTGGCCTCGCTGAGGCTGACCC

GCCCTTCTGTTTTGGAACCAAACCTaaATCATCCAGTAAACCGCC

CCTCGTAAATCCTCATCAaaCCGGGTAGTGGGTCCTCTCGAAGAC

TGGCGAGCTCCTCCCTCACGAAGGCaaATCATCCAGTAAACCGCC

CCTCGTAAATCCTCATCAaaGAATGTCGTCCTGTTCCTACGGGCC

CTCGAGGGCGGCCAGTTGCGTGCTGaaATCATCCAGTAAACCGCC

CCTCGTAAATCCTCATCAaaCCTCTGACTCTGAGTCTTTGCCGTC

CCCCCTTGCCTCCGCCCTCCTCCTTaaATCATCCAGTAAACCGCC

CCTCGTAAATCCTCATCAaaGCTGGAGGGTTTAATGTGGATGCAG

TGACTTCTTGTCGAGGTCCGGACTGaaATCATCCAGTAAACCGCC

CCTCGTAAATCCTCATCAaaTTGTTGTTGTTCATGAGTTTGTTTG

GAAGACAGGTTGCTGGGATTGGACGaaATCATCCAGTAAACCGCC

CCTCGTAAATCCTCATCAaaTGTCCAGTCTATCGGCCATCCTGTC

AGAGGGGAGGGGAGGCCGGGCTGGGaaATCATCCAGTAAACCGCC

CCTCGTAAATCCTCATCAaaAGAGGGAATCCTTTCGGAGGGGATC

CGGGATCCTGTCGGAGGGGATCCTGaaATCATCCAGTAAACCGCC

CCTCGTAAATCCTCATCAaaTGGGCCGAGCCCCCGGAGTGAGGCT

TCGGGCACCGTGTGCAGCAACATGCaaATCATCCAGTAAACCGCC

CCTCGTAAATCCTCATCAaaGGGTTTGTTGAGGGCGTTGTAATGT

GTCCAGCAAGTGGCTCACCGAGAAGaaATCATCCAGTAAACCGCC

CCTCGTAAATCCTCATCAaaGGAGAGTAGTAAGCTGAGGCAATTC

AACTTGTGGTCGTAGTATGCCGCTGaaATCATCCAGTAAACCGCC

CCTCGTAAATCCTCATCAaaAGGCATGTAGTGGTGGGATTGTTGC

TCACTGAGCCATCTGGTGGTGGTGGaaATCATCCAGTAAACCGCC

CCTCGTAAATCCTCATCAaaAGAGTGGCAATGCTCGACCCCACCG

ATATTATATTCACGCGCTTTCAGTCaaATCATCCAGTAAACCGCC

CCTCGTAAATCCTCATCAaaCAGCGTACGCACAGCTGGAGGAAGA

GAGGTGCCGCCGAGTACACACCTTGaaATCATCCAGTAAACCGCC

CCTCGTAAATCCTCATCAaaCATGTTGTAGCCAGGAGAGCTGGCC

GGTCGAGGTGCCCATTCCGGCGGGAaaATCATCCAGTAAACCGCC

CCTCGTAAATCCTCATCAaaCAGCCCAAGTACTCGGGACTGAGGG

GGGCTGTAAGTGCCCCCTGCCCCGGaaATCATCCAGTAAACCGCC

CCTCGTAAATCCTCATCAaaGGGGACCTGAAGACTCCATCCGCGG

TGGGTCGTGGGCCAATGGGTTGCTCaaATCATCCAGTAAACCGCC

CCTCGTAAATCCTCATCAaaCATGTTACGTTCGTTGCGTCGGAAT

GCCGTACAGTGATGACCTCTGGGCAaaATCATCCAGTAAACCGCC

CCTCGTAAATCCTCATCAaaACCCTGGCCTCGCTGAGGCTGACCC

GCCCTTCTGTTTTGGAACCAAACCTaaATCATCCAGTAAACCGCC

CCTCGTAAATCCTCATCAaaCCGGGTAGTGGGTCCTCTCGAAGAC

TGGCGAGCTCCTCCCTCACGAAGGCaaATCATCCAGTAAACCGCC

CCTCGTAAATCCTCATCAaaGAATGTCGTCCTGTTCCTACGGGCC

CTCGAGGGCGGCCAGTTGCGTGCTGaaATCATCCAGTAAACCGCC

CCTCGTAAATCCTCATCAaaCCTCTGACTCTGAGTCTTTGCCGTC

CCCCCTTGCCTCCGCCCTCCTCCTTaaATCATCCAGTAAACCGCC

CCTCGTAAATCCTCATCAaaGCTGGAGGGTTTAATGTGGATGCAG

TGACTTCTTGTCGAGGTCCGGACTGaaATCATCCAGTAAACCGCC

CCTCGTAAATCCTCATCAaaTTGTTGTTGTTCATGAGTTTGTTTG

GAAGACAGGTTGCTGGGATTGGACGaaATCATCCAGTAAACCGCC

CCTCGTAAATCCTCATCAaaTGTCCAGTCTATCGGCCATCCTGTC

AGAGGGGAGGGGAGGCCGGGCTGGGaaATCATCCAGTAAACCGCC

CCTCGTAAATCCTCATCAaaAGAGGGAATCCTTTCGGAGGGGATC

CGGGATCCTGTCGGAGGGGATCCTGaaATCATCCAGTAAACCGCC

CCTCGTAAATCCTCATCAaaTGGGCCGAGCCCCCGGAGTGAGGCT

TCGGGCACCGTGTGCAGCAACATGCaaATCATCCAGTAAACCGCC

CCTCGTAAATCCTCATCAaaGGGTTTGTTGAGGGCGTTGTAATGT

GTCCAGCAAGTGGCTCACCGAGAAGaaATCATCCAGTAAACCGCC

CCTCGTAAATCCTCATCAaaGGAGAGTAGTAAGCTGAGGCAATTC

AACTTGTGGTCGTAGTATGCCGCTGaaATCATCCAGTAAACCGCC

Gene: *Platynereis* *col6a6*

Matching ID in draft genome annotation: XLOC- 015056

GAATAGGGCTTGCAGAAAGATTTAGAGTTTGCTTAAAGTTTATTTTCGAGTCTGTAGCTAGCACCAGTGGATGAGGCTTTGAACTGAATAAGGTCAGAATGCCTCGTCTAGGGTTTTTAGTTTTGCTTGCAGCCGTGTTTGCTGTACAGAATGCAGATGCTCAAAGAGACAGATGCAGGTCCAGCACTGATTTCATCTTTGTTCTGGACTCATCCGGAAGTCTGGTGGATCAGCCGCTAAGAACGAGCAACTGGGGGAACATCACGGAATTCCTGGAGATAGCCGTTCTCAATATTGCGGACACAATTGAGAGGAACTATACAGGAGCATCTGCCGGATTGCGGTTTGGTCTCGTCCAGTTCCGAGACATAGGGGTCATCACATTCGGTCTCAATGACTTCAGAGACTACCGCCGAGCAGCTGAAATCATGGGACTTGCAACAAACCTTGGCGGACAGACAAACATGGCCGCCGGCCTGGAGGAGGTAGAAAAAATGCTCGGTCCACCACGAGCCAACAATGTCAGAAAAACGGTCATCATTGTTGTGTCGGACTGGAGTCCATTTAACGACACTGTCAGGACAAACTCAGTCCTGGCGGCCCGAAGACTGGAGCGTATGTATGATGCTAGCATTATAACCGTGGGAGTCCTTGGATCGGTTGAGCGGGATTTCCTAATGGACGTTTCAACAGGAGGAAGAAATGTCATGTCCGAGGACCACGCTACTTTAATAGAACATGTTTCTAGTTTATTACAAGTTGTCTGCGAGACTCCAGGTGGCGGCACTGGTGGGCCAGGAGGGCCCTGTGAAAGAGATGTTGTTTTTGCTATCGACGCCTCCTACAGTTTATCTCGGCAACAAGACTTCATGCGGCTGAAAACCTTTGTTTCTCAAGTGGCTGGAAATATTTTCCAGACTGCGAGAGACACTCGGATGGGGGTGGTGGTCTTCAGTACCACTGCCAATGTTACTATTCCTTTGCAGTTGTACACCCAGAACTTCGCACGACAAGTGGAAGCAATAAGTTTCGACCCGAGGTTCACCAACCCCGCAGATGCCCTCAGGAAAGCTCGCGAAATGCTCCAAGGAAGCAATCGTTCCAAGTTTGTTATAATTTTCACGGATGGTTTGACGAACGAAGAACAAGGGGAGGAGGCTATCATCGCTGAGGAGATGAGAAATGACGATGGAATCGTCATAATTGCTTTTGGTGTCAGTGATAAAATTGATTTTGCAACCCTGGAGAAGTTGGTCGGAAGTGATAAAACAGGTTCAAACATCTTTTGGAGTCCGGATTACCAGACGAACATGATCTCGAAGCTAAACACGTTAACCTTGACTGCTGAGGGGTGTCCTGCTAGGGACATACCTTGTGACATTAGGCCTCAAGAAGTGATTGTTGCACTGGATGCCTCAGGAAGTATCAAGGACAAAGGTCCAAACAACTGGCAACGAATGCAAGACTTTGGCGCTGCGGTGGTGCAAAGCATCCACAGGACTTATCCCGACAGCCGGGTGGGAGTGCTCATCTTCAGTGATTTTGCGAGTGAAATAATTCCACTAACAGCTACGGAGAACACAAGGGGAACACAGAGAATCATCGCAGCGATAAGGAATGCAGAGCATGTGAACGGATATACCAACGAAGCCGACGCCTTGAGAGTTGCGATGAGGATGTTGCAAGGAGCTAGGAATCCATCCTACAGATATGTTGTCATGGTAACAGATGGCCAGCCGACACAGGAAATTGGCCAAGAAATCAACGAAGCTGATACGCTAGAGAGTAACGGCATCACACGTATTATGATTGGTGTGACAAGTGACATCAAGGAAGAAAGGTTGAAAAGATTGGGTTCCTCTCCTTCCGATCGTTACGTTCTCTTTACGAAAGACTTTAACACTCTTAATGCTGTTGGTCTGATCACTTCTGTAGTCACGGCTGCCAGCCAATGTGACCAAACACAACAAGCCAGACCTGTAGCTACTTCACCGCCAAGACAGTGTTTTGGAGCTGATGTTATTCTAGTGATGGATAGCTCAGGCAGCATTGAAGAAAAGGGAAAAGGAAATTTTGAACTCTTGAAAACATTCACTGCCACCTTAGCCTCAAATATTGCCAATAGATATCCGCAAACCAAATTCGGACTTGTGCTGTTCAGTGATGTAGGGCGAGTGGTATCCAGTCTAAGGGATTACAGGTCCATCCTCGGCTTTATCGATGTCATCGGGCAGCAGCGGTACAACGGAAGTCAGACAAACATTGCATCAGCCCTCAGGGTGGCCAGACAGGATGTGGTTCAGTATGGACAGAACAACAGACAGATCATCATCATAACTGATGGAACCCCAACGGCCGAAGTGGACCAAACAGTTCGAGAAGCAGAACTTGCAAGACGAGCCGATATCCAGATATCCGCTGTTGGTATCACCTATGATATAGCAGAAGAGACCCTAGGAGGCATCACCGGAAGTTCCAGTAGAGTGTTCATGACTCCTAAATTCACCGACCTACAAAATGAGATCGTCAAAGTGACGCAGGTTGCCTGTGAACCTGGAAGTGCGGCAAGAGCTGGTGCACCATGCTCTGGTGACTTGGAGGTGGTTTTCTCCCTAGATGCATCCGGTAGCATAAAAGACCTTTCTCCCACAGCATGGGAAGATGCTAAAAACCTTGTCAAGTCTGCGATCAACGCCTTTTACAACTTCAACAGAAACACGAGATTCGGTCTTGTGATATTCAGTGACTTTGGTCGACGAGAATTTGGACTCTCTGGTGATGTAAACGACCTTCCACGGTTGGTCGACGCGGCCTTTTATAATGACGGACGAACAAACATCGCAGATGGCATTGAAATCGCCCGTAGGGATGTATTCTCTGCATCAAGACCAGGAGCCAATCAAATCTTGATCCTCATAACAGATGGTGAACCAAACGAAAGGGTTAATGACACTGATAGAGAGGCTGGAATTGCTAAGAGGGATGGAATTACAATTGTATCTGTAGGTATTACTGAAGACGGCCCTGATTATGCTCTCCTCACAAGGGTGGCCAGCAGCCCCAGCCAAGTCATCAGGTCCCTCAGTTTCAGCAGCCTCAGTTCCCAGATCAACAATCTTGTGTCTACATCTTGTCAGGGAGCCTTAACCACACGACCTAACCCTAATGTTGTCGGCGGTGGTTTCGGAGGGTTTACCACTAAGCCTGGCCGTTTCTTTCCATTCAGACCTGCTGGCACCACTCCCAGAATTTTAAATAGAGTTCCAGGTGGTATTGTTACCACTCCCATTAGACCACAACGACCTGCATTTACAGCGCCACCCAGAGGGCCTGGTCGCACCTTAGGCATTTACCCTACCACAGCACTACCGAGACCACCAATAATTAACAATGTTGGGCCTACCCCAATTTCGTTGGTTTTTGACATTCCATCTGTTCCATGCATTGGAGGGTATGTCGTACTAGTCTTGGATGTCTCCCGGCGATTGAACTACAAGACTTTCCCAAGCAATGGCACAAGGGGATTACTTGCCATGGCAGCAAATATCATTACTGTCATGTCACAAATGAAGAGGGATGCAAAATTTGCTATTGTTACTTATAACGCCACAGCACAGGTTCAAAGAGATTTTGGAATTTACAGGCCCGGGGAGTTGGAGAGTTTCCTACAGAATCTGAATCCTGCACAATCCTATGACTCGAATATTGCTGATGGATTCGCCAAAGCTCAAGAAGTTCTACAGAGATCCAGAGCTTCTAGATCACTGGTTGTTCTTGTCACTGATGGGGAAGCCACCCTAGATTCTGGAATTGAACAAACTCGAGCCGATCAATTAAGAACATTTGGCAGCAAGGTGGTTGTCATTGCTGTATCAAAGAACTTCAATGAGAACAAACTGCGAGCCATCACTAAAAATGACCCATCTCACTTTGTTGCTCAGTCATATAACTTTGACCCTAACTTGCTTGAGCCTTATTACCAAGGATGCAGTAGAAATCCAAACTCAGTCGCTGCACTCAGAGATGATGACACGGAAGTTTGTCCTAAAGAAGTGGTCTTCCTAATTGATAGTTCAGGAAGCATAAGTGACTCCCAGTGGGAACAAATGAAATTCTTCTTAATCACTCTTGTCGACAAATTTTCTGAAAAATTGGATGGAGTCAAATTTGGTACCGTAATGTTCGGAAATAGTGCCCAGTTGGGCTTCTACTTGTCAGATGACTTGTATGATGTGAAAACTCGTCTTCAGAATCTGCGTCAATTGAGAGGACAAACGAATATGGCGAGAGGCTTGAGCGTGACAAGACGATACATGTTCAGTGACAACGATGCCAAGAAGTTGGTGCTGTTAGTTGCCGAGGGAAATCCAACAAGTCGATTATCAGAAACGTACCAGGAAGCGGATAAGCTACGAGAAGATGTCGATCTGAAGGTAGTCGGTCTCACAGATGTACCAGCAAAGACCATGTTGGACAGAATCGCAGGATCGGAAGATAAAGTTGTCCGCATTAACGGTTTCTCAGATTTGTTAAACTCAGTTGACACTGTTCGCGATCTTGGTTGTAAAGCCGGTCAACCAGAACAACAACCATGAGTCATTCAATATGATGGTTATGTTGGTTATATTGTATATTGTGTATGCATATTGAATATCTCAACATATATTGAATGGATTAACTGCCTTTGGACCGAGTGTCTTTCAATTATATCAAATATTATATCAAATATTTTTTGAAGAATTTTATACTTTTATATGACTTTTATGATATATTAAAATGCTCAAAATTCAACGAGGAAAAGAGTATTGAACTAATACCGAATATTCCCGGAGAACCTGAAATAGCTTGTGTCAACCATGTACTTGAGTGAAATAAGGAAAACGGGGGAATAAATTTTACTAGAACCGTAGAAATTAAACATTATCTTGATTTCTCGATTGTGCTGAAACGTAGCAAGGAAAACATATGAAAAATCACTGATTTGCCTATCGTATTGATGAAGTGACAAGTACATCATATTGTTGGATATACTTCTGAAACAGTTTATCATAATGGATTAATCAAATAGACAGTACTCATGGATTTATGGTGACCTTTATAACAGAAATACAAAGTGATGAATTCATCTTGCATATATTGGACTTACGCCTATGATTTTTACATGTCTCATGGATTAGTTGATGTTAAACATGTGTACAAAGAAATTGTGAAAAGTAAATGATCCAAATTAGGGCTAAAGGTGAACTCCCGTTTGCAGCAACGAGTCAACATGAGACCTCCGTCATCATTCTTTTCTCTGCCTTATTTCTTCAAGGCTTCAAGCCGTGTCCTTTCTGCTCATATTCATAGACGTTCCAGAGACTACGACAATTTTTTATAGAATCTTACGCTGAGGTATTTAAGGTAATTCTGTTATAGATTGATCGATAAAGATACAAATGTAATTGGCCAATGAACAATGGTGCTGGTTTAAGGGACTTTAGCATATGCATGTAGAGCTAGCTATTTTACTGCCATATTTTTATCCATATTGTACTATTACCATTTATACGAATTTTGCAAATTATTTTTCAATTTTGGTTCACTGCATGTACTTGCAGAAAGACATATTGGACTCACAGACATGAGTTGAATGATATCTGCCCAATGTTATCTTTTGTTGCTCGTAAATTATCAAAATACATTCCAAAACAATTGACATAATACAACAACTGACAATATGGCAGTTGTGTATTATCTTAACATTCCTGCAATTTCTTTGTTACAAACTTTAACTTTGTTATATTTTTACCTTCTCTGTAAGTTTACATATTTATAATATATTGTGTAATTGGAATTTGATTATAATTGTTACTTATAATGGTATGTGATATGTAGTTAATTTGAACCTAATTCCACTGAATGTATGCATTTTTATTCTGTAAATGGTGAGAAGTGATATATGATAGTGTACTTTATTTGGGTGAACAAATTATTCTGCAAGATAATTATAATTTACTAATGTTTAACCATGACACACTGCCTGATGTAATAAAGAATAAAGATATTAAATGACAATAAAAAAAAAAAAAAAAAAATTGTGCGGAAGGGGGGGGGG

HCR Probes (B1 adapter):

GAGGAGGGCAGCAAACGGaaTCCATCAGCAATATTCGAGTCATAG

CTGTAGAACTTCTTGAGCTTTGGCGtaGAAGAGTCTTCCTTTACG

GAGGAGGGCAGCAAACGGaaCTCTCCAACTCCCCGGGCCTGTAAA

TGTGCAGGATTCAGATTCTGTAGGAtaGAAGAGTCTTCCTTTACG

GAGGAGGGCAGCAAACGGaaTGGAAAGAAACGGCCAGGCTTAGTG

TCTGGGAGTGGTGCCAGCAGGTCTGtaGAAGAGTCTTCCTTTACG

GAGGAGGGCAGCAAACGGaaTTAGGGTTAGGTCGTGTGGTTAAGG

AACCCTCCGAAACCACCGCCGACAAtaGAAGAGTCTTCCTTTACG

GAGGAGGGCAGCAAACGGaaTGATCTGGGAACTGAGGCTGCTGAA

CCTGACAAGATGTAGACACAAGATTtaGAAGAGTCTTCCTTTACG

GAGGAGGGCAGCAAACGGaaGCTGGCCACCCTTGTGAGGAGAGCA

GAGGGACCTGATGACTTGGCTGGGGtaGAAGAGTCTTCCTTTACG

GAGGAGGGCAGCAAACGGaaGATACAATTGTAATTCCATCCCTCT

TCAGGGCCGTCTTCAGTAATACCTAtaGAAGAGTCTTCCTTTACG

GAGGAGGGCAGCAAACGGaaTAACCCTTTCGTTTGGTTCACCATC

CAATTCCAGCCTCTCTATCAGTGTCtaGAAGAGTCTTCCTTTACG

GAGGAGGGCAGCAAACGGaaTGGTCTTGATGCAGAGAATACATCC

TATGAGGATCAAGATTTGATTGGCTtaGAAGAGTCTTCCTTTACG

GAGGAGGGCAGCAAACGGaaTTTGTTCGTCCGTCATTATAAAAGG

CGGGCGATTTCAATGCCATCTGCGAtaGAAGAGTCTTCCTTTACG

GAGGAGGGCAGCAAACGGaaGAATCTCGTGTTTCTGTTGAAGTTG

ACCAAAGTCACTGAATATCACAAGAtaGAAGAGTCTTCCTTTACG

GAGGAGGGCAGCAAACGGaaCTAGGGAGAAAACCACCTCCAAGTC

AAAGGTCTTTTATGCTACCGGATGCtaGAAGAGTCTTCCTTTACG

GAGGAGGGCAGCAAACGGaaACTTCCAGGTTCACAGGCAACCTGC

AGAGCATGGTGCACCAGCTCTTGCCtaGAAGAGTCTTCCTTTACG

GAGGAGGGCAGCAAACGGaaGTGAATTTAGGAGTCATGAACACTC

ACTTTGACGATCTCATTTTGTAGGTtaGAAGAGTCTTCCTTTACG

GAGGAGGGCAGCAAACGGaaTCTCTTCTGCTATATCATAGGTGAT

TGGAACTTCCGGTGATGCCTCCTAGtaGAAGAGTCTTCCTTTACG

GAGGAGGGCAGCAAACGGaaTGGCCACCCTGAGGGCTGATGCAAT

TCTGTCCATACTGAACCACATCCTGtaGAAGAGTCTTCCTTTACG

GAGGAGGGCAGCAAACGGaaCCCGATGACATCGATAAAGCCGAGG

TGTCTGACTTCCGTTGTACCGCTGCtaGAAGAGTCTTCCTTTACG

GAGGAGGGCAGCAAACGGaaTTTTCCCTTTTCTTCAATGCTGCCT

GAATGTTTTCAAGAGTTCAAAATTTtaGAAGAGTCTTCCTTTACG

GAGGAGGGCAGCAAACGGaaCCAAAACACTGTCTTGGCGGTGAAG

CTATCCATCACTAGAATAACATCAGtaGAAGAGTCTTCCTTTACG

GAGGAGGGCAGCAAACGGaaCACATTGGCTGGCAGCCGTGACTAC

CTACAGGTCTGGCTTGTTGTGTTTGtaGAAGAGTCTTCCTTTACG

GAGGAGGGCAGCAAACGGaaTTCAACCTTTCTTCCTTGATGTCAC

CGATCGGAAGGAGAGGAACCCAATCtaGAAGAGTCTTCCTTTACG

GAGGAGGGCAGCAAACGGaaCGTTACTCTCTAGCGTATCAGCTTC

TCACACCAATCATAATACGTGTGATtaGAAGAGTCTTCCTTTACG

GAGGAGGGCAGCAAACGGaaCTGGCCATCTGTTACCATGACAACA

GATTTCTTGGCCAATTTCCTGTGTCtaGAAGAGTCTTCCTTTACG

GAGGAGGGCAGCAAACGGaaAACATCCTCATCGCAACTCTCAAGG

CTGTAGGATGGATTCCTAGCTCCTTtaGAAGAGTCTTCCTTTACG

GAGGAGGGCAGCAAACGGaaGCTCTGCATTCCTTATCGCTGCGAT

CGGCTTCGTTGGTATATCCGTTCACtaGAAGAGTCTTCCTTTACG

GAGGAGGGCAGCAAACGGaaTGCAACAATCACTTCTTGAGGCCTA

GTCCTTGATACTTCCTGAGGCATCCtaGAAGAGTCTTCCTTTACG

GAGGAGGGCAGCAAACGGaaCCGGACTCCAAAAGATGTTTGAACC

GCTTCGAGATCATGTTCGTCTGGTAtaGAAGAGTCTTCCTTTACG

GAGGAGGGCAGCAAACGGaaGGTTGCAAAATCAATTTTATCACTG

TTTATCACTTCCGACCAACTTCTCCtaGAAGAGTCTTCCTTTACG

GAGGAGGGCAGCAAACGGaaGCGAGCTTTCCTGAGGGCATCTGCG

GGAACGATTGCTTCCTTGGAGCATTtaGAAGAGTCTTCCTTTACG

GAGGAGGGCAGCAAACGGaaCTCCTGGCCCACCAGTGCCGCCACC

CAAAAACAACATCTCTTTCACAGGGtaGAAGAGTCTTCCTTTACG

GAGGAGGGCAGCAAACGGaaGTTGAAACGTCCATTAGGAAATCCC

TCCTCGGACATGACATTTCTTCCTCtaGAAGAGTCTTCCTTTACG

GAGGAGGGCAGCAAACGGaaTAATGCTAGCATCATACATACGCTC

CAACCGATCCAAGGACTCCCACGGTtaGAAGAGTCTTCCTTTACG

GAGGAGGGCAGCAAACGGaaCTGACATTGTTGGCTCGTGGTGGAC

TCCGACACAACAATGATGACCGTTTtaGAAGAGTCTTCCTTTACG

GAGGAGGGCAGCAAACGGaaCATGTTTGTCTGTCCGCCAAGGTTT

TTTTTCTACCTCCTCCAGGCCGGCGtaGAAGAGTCTTCCTTTACG

GAGGAGGGCAGCAAACGGaaGGACGAGACCAAACCGCAATCCGGC

ATGTGATGACCCCTATGTCTCGGAAtaGAAGAGTCTTCCTTTACG

GAGGAGGGCAGCAAACGGaaAATCAGTGCTGGACCTGCATCTGTC

TTCCGGATGAGTCCAGAACAAAGATtaGAAGAGTCTTCCTTTACG

Gene: *Platynereis* *piwi*

Matching ID in draft genome annotation: XLOC- 026648

ATGAGTGGCAGGGCACGCGGTCGGGCAAGGGGCCGTGCCCAAGGAGGAGCGGGCGACTCGGCTGCCCCAGCCCCTCGGCCAGGAGACACGGCTGCGGCCATGAGGACCCCCTCTGAGGCCCAGGCAGCACAGGCGGCCCATGCAGCAGCAGCGCCCCCTGCTCATGGCAGTGGTCGTGCAAGTCACCGTGGTGGAGCGAAGGTGGACCAGAGGCCGGGAGGGGCCCCCGTGGAGGGAATGGCAGGACTGAGCATTGGAGGAGACGACCGGGGACGCCGGGCACCTCAGATGAGGTATATCGAGCCAAGGACCAGACCTGCTGAGCTCCTTAGCAAAGTTGGTGGACATGGAACTGCTGTGCAGCTGCTGTGTAACTACTTCAAGTTGGAGAGCAAGCCAGACTGGCACCTGTATCAATACCACGTCTCGTTCGCCCCTGAGGTTGAGAGCAAGGGCACGAGGATGCGCCTCATCAAGGACCAGTCTTCTCACTTGGGACCCGTCCGAGCGTATGACGGGGGTGTCCTCTTCCTGCCCATCAAAATGCCCAATCCAGTGACTGAACTGACTGGCACCAAACGAGATGGCACTATCATACAGATCACGATCAAGTTAACCAACGAATTGCCCCCATCTGACCCCACCTGTATTCACGTTTACAACATCATATTCAACCGAGTATTGAAGATGATTGACATGAAACAAGTCGGTCGTCATTTCTTCTGTCCTGCCGAAGCAGTGACGATCGAAAGGCACAAGCTGGAGGTTTGGCCAGGTTTTATAACCTCCATTTTGCAATACGAGTCCTCCCTCCTCCTTTGTGCAGAGATCTCCCACAAGATCATGCGTTCCGACACCGTCCTGGACATTATGTACGACCTCCACAGGTCGCGTCGCGATGGTTTCCATGATGCTGCTGTTCGCAAATTAGTCGGCGAAATTGTTCTCACAAGGTACAACAACAAAACCTACAGAGTGGACGACATCAACTGGCAAATGAACCCAGCCTCTGAATTTGACACCCGAGATGGCAGCAAAATCTCTTTCTTCAAGTATTACGAAGAGCACCACAACCACAAAATCACGGACAAGGACCAGCCCCTGCTTGTATCCCGGCCAAAGAAGAAGGACATCCGACGTGGCATCCAGGGCGACATCCTGCTCATTCCAGAGCTCTGCACATTGACGGGACTCTCGGATGACGTCAGGGCCGACTTCAATGTCATGAGAGATGTGTCTGCCCAGACGCGCTTGGACCCTGAAGCCAGAATAAGAGAGCTGGACACCTTCAGGAAAAAGATTGCTACAAATGAGCAGGCAGTGAAGGAGCTGCAGGGATGGGGCTTGAAGTTCTCGCCAAATGTGGTCCAAGTGGGTGGCCGAGTGTTGCCCCCTGAGAACATCTGCCAGGGCAACAAACGCTTCAGCTACAAGCAGGCGGATGCCGATTGGTCGAGGGAGATGCGTGGAGTGCCACTGATCAGCTGTGTGCCTCTGAGGAACTGGATCATGGTCCACACAGGAAGAAACGAACAAGTTGCCATGGAATTCCTGACCAATCTGAAGAGGGTCGGCCCCCCTATGGGAATTCAAGTCTCCGATCCTACTAGGATCCCATTGCGTGATGAGAACAACAACACTTTCCTTAGTGCCATCAAACAAGCACTGAACGACAAGACTCAGCTGGTGCTATGTATCCTGCCCTCAAACAAGAAGGACCGATATGATGCTATCAAGAAGCTCTGCTGCATCAACTGTCCAGTTCCCTCTCAGATGGTGCTTGCCAAGACCTTGAGCAAAGCCAAGATGTTGATGTCCGTGGCGACCAAGATTGCCATCCAGATCAACTGCAAGTTGGGAGGAGAGGTTTGGGCCCTGGAGATCCCTTTGAAAGGCCTGATGGTGGTGGGCGTGGACAGCTACCACGACTCCAAGCAGAAGGGTCGCTCAGTGGGTGCTTTCGTGGCGTCCATGAACCAGAGCTTGACTCGATACTACTCTCGATGCTGCTTCCAGCATCAACACCAGGAGTTGCTGGATGGACTGCGCGTGTGTGCAACTGGTGCCCTGAAGGAGTACCACAAATTGAATCAAGCCCTGCCCCAGAGGATCATCATCTACAGAGATGGAGTGGGAGATGGCCAGCTGGGTCAGGTGGTGGAGCATGAGATCCCTCAGCTGCTTAAGTGCTTCTCAACCATGGGCCAGGACTACAGCCCGAAGACCACTGTTGTTGTCGTCAAAAAGAGGATCAACACAAGATTCTTCGCCTCTGGGGCTAGGAGCTTGGCTAACCCCATGCCAGGAACAGTTGTGGATCAGGTGGTCACCAAACCTGAATGGTACGATTTCTTCGTGGTGTCACAGTCAGTGCGCCAAGGAACAGTGAGTCCAACTCACTACAACGTGGTCTGGGACAGCAACTCCCTAGGACCAGACAAGATGCAGCGGCTGACCTACAAAATGACACATCTCTACTACAACTGGCCTGGAACAATCCGTGTGCCGGCTCCTTGCCAATACGCCCACAAGATGGCCTTCCTCGTTGGCCAGTCGCTTCATGAAGAGCCAGCCATGGATCTGGCCGACCGTTTGTATTACTTGTAA

HCR Probes (B1 adapter):

GAGGAGGGCAGCAAACGGaaATCCATGGCTGGCTCTTCATGAAGC

TTACAAGTAATACAAACGGTCGGCCtaGAAGAGTCTTCCTTTACG

GAGGAGGGCAGCAAACGGaaGCGTATTGGCAAGGAGCCGGCACAC

TGGCCAACGAGGAAGGCCATCTTGTtaGAAGAGTCTTCCTTTACG

GAGGAGGGCAGCAAACGGaaCCAGACCACGTTGTAGTGAGTTGGA

CTTGTCTGGTCCTAGGGAGTTGCTGtaGAAGAGTCTTCCTTTACG

GAGGAGGGCAGCAAACGGaaACCACGAAGAAATCGTACCATTCAG

ACTGTTCCTTGGCGCACTGACTGTGtaGAAGAGTCTTCCTTTACG

GAGGAGGGCAGCAAACGGaaTTTGTGGTACTCCTTCAGGGCACCA

GATCCTCTGGGGCAGGGCTTGATTCtaGAAGAGTCTTCCTTTACG

GAGGAGGGCAGCAAACGGaaTCCTGGTGTTGATGCTGGAAGCAGC

GCACACACGCGCAGTCCATCCAGCAtaGAAGAGTCTTCCTTTACG

GAGGAGGGCAGCAAACGGaaTCATGGACGCCACGAAAGCACCCAC

GAGAGTAGTATCGAGTCAAGCTCTGtaGAAGAGTCTTCCTTTACG

GAGGAGGGCAGCAAACGGaaGCTGTCCACGCCCACCACCATCAGG

GCGACCCTTCTGCTTGGAGTCGTGGtaGAAGAGTCTTCCTTTACG

GAGGAGGGCAGCAAACGGaaTCTCCTCCCAACTTGCAGTTGATCT

TTCAAAGGGATCTCCAGGGCCCAAAtaGAAGAGTCTTCCTTTACG

GAGGAGGGCAGCAAACGGaaGTTGATGCAGCAGAGCTTCTTGATA

AAGCACCATCTGAGAGGGAACTGGAtaGAAGAGTCTTCCTTTACG

GAGGAGGGCAGCAAACGGaaAGGATACATAGCACCAGCTGAGTCT

TCATATCGGTCCTTCTTGTTTGAGGtaGAAGAGTCTTCCTTTACG

GAGGAGGGCAGCAAACGGaaGGAAAGTGTTGTTGTTCTCATCACG

CGTTCAGTGCTTGTTTGATGGCACTtaGAAGAGTCTTCCTTTACG

GAGGAGGGCAGCAAACGGaaGGAATTCCATGGCAACTTGTTCGTT

GGGGGCCGACCCTCTTCAGATTGGTtaGAAGAGTCTTCCTTTACG

GAGGAGGGCAGCAAACGGaaCAGAGGCACACAGCTGATCAGTGGC

TCCTGTGTGGACCATGATCCAGTTCtaGAAGAGTCTTCCTTTACG

GAGGAGGGCAGCAAACGGaaTCCGCCTGCTTGTAGCTGAAGCGTT

CCACGCATCTCCCTCGACCAATCGGtaGAAGAGTCTTCCTTTACG

GAGGAGGGCAGCAAACGGaaACACTCGGCCACCCACTTGGACCAC

TGCCCTGGCAGATGTTCTCAGGGGGtaGAAGAGTCTTCCTTTACG

GAGGAGGGCAGCAAACGGaaCAGCTCCTTCACTGCCTGCTCATTT

TGGCGAGAACTTCAAGCCCCATCCCtaGAAGAGTCTTCCTTTACG

GAGGAGGGCAGCAAACGGaaTCTCTTATTCTGGCTTCAGGGTCCA

GCAATCTTTTTCCTGAAGGTGTCCAtaGAAGAGTCTTCCTTTACG

GAGGAGGGCAGCAAACGGaaCATTGAAGTCGGCCCTGACGTCATC

GCGTCTGGGCAGACACATCTCTCATtaGAAGAGTCTTCCTTTACG

GAGGAGGGCAGCAAACGGaaAATGAGCAGGATGTCGCCCTGGATG

GAGTCCCGTCAATGTGCAGAGCTCTtaGAAGAGTCTTCCTTTACG

GAGGAGGGCAGCAAACGGaaGATACAAGCAGGGGCTGGTCCTTGT

CGTCGGATGTCCTTCTTCTTTGGCCtaGAAGAGTCTTCCTTTACG

GAGGAGGGCAGCAAACGGaaAATACTTGAAGAAAGAGATTTTGCT

TGATTTTGTGGTTGTGGTGCTCTTCtaGAAGAGTCTTCCTTTACG

GAGGAGGGCAGCAAACGGaaGTTCATTTGCCAGTTGATGTCGTCC

ATCTCGGGTGTCAAATTCAGAGGCTtaGAAGAGTCTTCCTTTACG

GAGGAGGGCAGCAAACGGaaAGAACAATTTCGCCGACTAATTTGC

CTGTAGGTTTTGTTGTTGTACCTTGtaGAAGAGTCTTCCTTTACG

GAGGAGGGCAGCAAACGGaaGCGACCTGTGGAGGTCGTACATAAT

CAGCAGCATCATGGAAACCATCGCGtaGAAGAGTCTTCCTTTACG

GAGGAGGGCAGCAAACGGaaGTGGGAGATCTCTGCACAAAGGAGG

CAGGACGGTGTCGGAACGCATGATCtaGAAGAGTCTTCCTTTACG

GAGGAGGGCAGCAAACGGaaGACAGAAGAAATGACGACCGACTTG

GCCTTTCGATCGTCACTGCTTCGGCtaGAAGAGTCTTCCTTTACG

GAGGAGGGCAGCAAACGGaaGAATATGATGTTGTAAACGTGAATA

CATGTCAATCATCTTCAATACTCGGtaGAAGAGTCTTCCTTTACG

GAGGAGGGCAGCAAACGGaaGTTAACTTGATCGTGATCTGTATGA

GTGGGGTCAGATGGGGGCAATTCGTtaGAAGAGTCTTCCTTTACG

GAGGAGGGCAGCAAACGGaaCAGTCACTGGATTGGGCATTTTGAT

TGCCATCTCGTTTGGTGCCAGTCAGtaGAAGAGTCTTCCTTTACG

GAGGAGGGCAGCAAACGGaaTCGGACGGGTCCCAAGTGAGAAGAC

CAGGAAGAGGACACCCCCGTCATACtaGAAGAGTCTTCCTTTACG

GAGGAGGGCAGCAAACGGaaTTGCTCTCAACCTCAGGGGCGAACG

TCCTTGATGAGGCGCATCCTCGTGCtaGAAGAGTCTTCCTTTACG

GAGGAGGGCAGCAAACGGaaGCTTGCTCTCCAACTTGAAGTAGTT

CGTGGTATTGATACAGGTGCCAGTCtaGAAGAGTCTTCCTTTACG

GAGGAGGGCAGCAAACGGaaACCAACTTTGCTAAGGAGCTCAGCA

CAGCAGCTGCACAGCAGTTCCATGTtaGAAGAGTCTTCCTTTACG

GAGGAGGGCAGCAAACGGaaTGAGGTGCCCGGCGTCCCCGGTCGT

CTGGTCCTTGGCTCGATATACCTCAtaGAAGAGTCTTCCTTTACG

GAGGAGGGCAGCAAACGGaaGGGCAGCCGAGTCGCCCGCTCCTCC

CAGCCGTGTCTCCTGGCCGAGGGGCtaGAAGAGTCTTCCTTTACG
