# Supplementary material for: Molecular profiles, sources and lineage restrictions of stem cells in an annelid regeneration model
Source: Nat Commun. 2024 Nov 18;15:9882. doi: 10.1038/s41467-024-54041-3 (PMC11574210; doi:10.1038/s41467-024-54041-3)
Supplement: Supplementary file 9 — Supplementary Data 6 [file 41467_2024_54041_MOESM9_ESM.pdf]

Supplementary Data 6:

Molecular phylogenetic analyses of selected genes

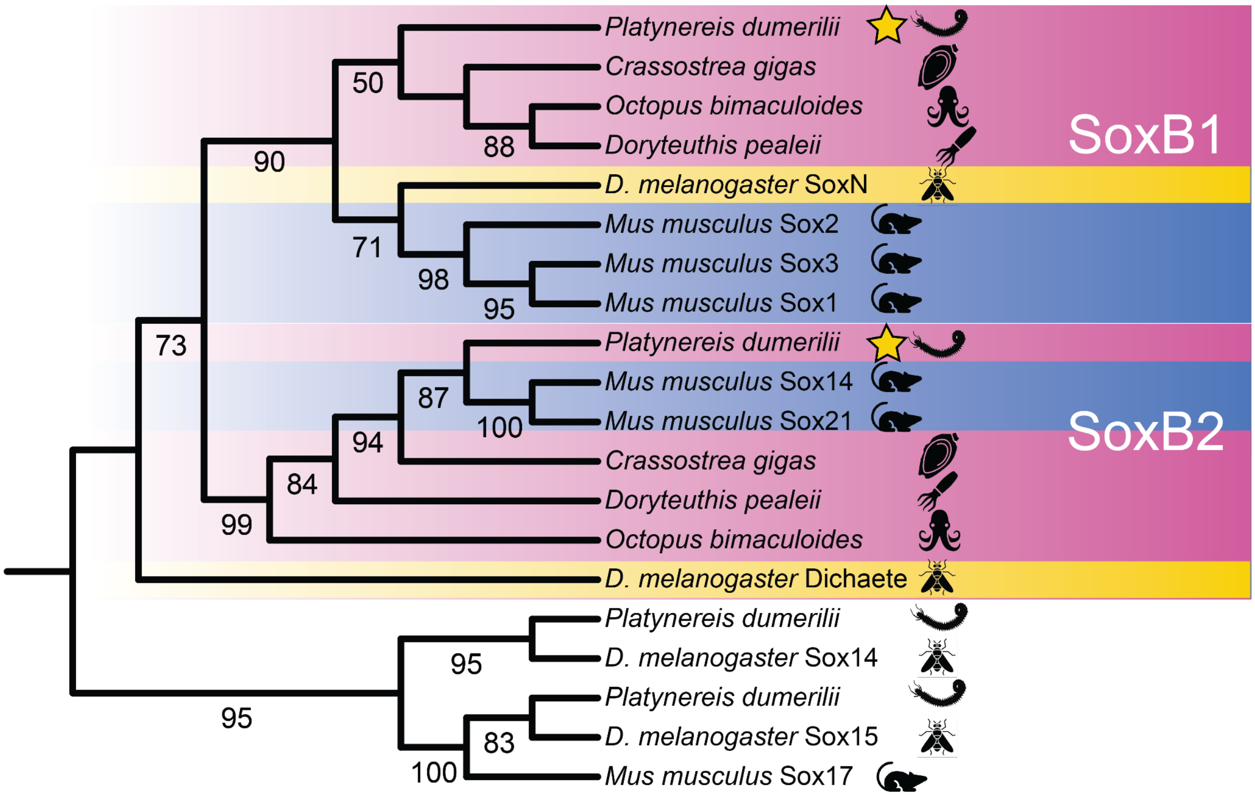

Supplementary Data 6 Figure 1. Orthology relationships of *Platynereis* SoxB proteins. Sequence identifiers: **SoxB1 group:** KT266552.1 (*Platynereis dumerilii*), XP_011455662.1 (*Crassotrea gigas*), XP_014780771.1 (*Octopus bimaculoides*), OM481471.1 (*Doryteuthis pealeii*), NP_524735.1 (*Drosophila melanogaster*), ENSMUSP00000137203, ENSMUSP00000096755, ENSMUSP00000115237 (*Mus musculus*); **SoxB2 group:** SoxB2 group: ANS60443.1 (*Platynereis dumerilii*), ENSMUSP0000009131, ENSMUSP00000127396 (*Mus musculus*), XP_011433975.1 (*Crassostrea gigas*), OM481470.1 (*Doryteuthis pealeii*), XP_014789971.1 (*Octopus bimaculoides*), NP_524066.1 (*Drosophila melanogaster*); Outgroup: P40656.3, P40657.2 (*Drosophila melanogaster*), Q61473.1 (*Mus musculus*), *Platynereis dumerilii:* CAY12635.1, this study. Bootstrap support (in %) is indicated below relevant nodes.

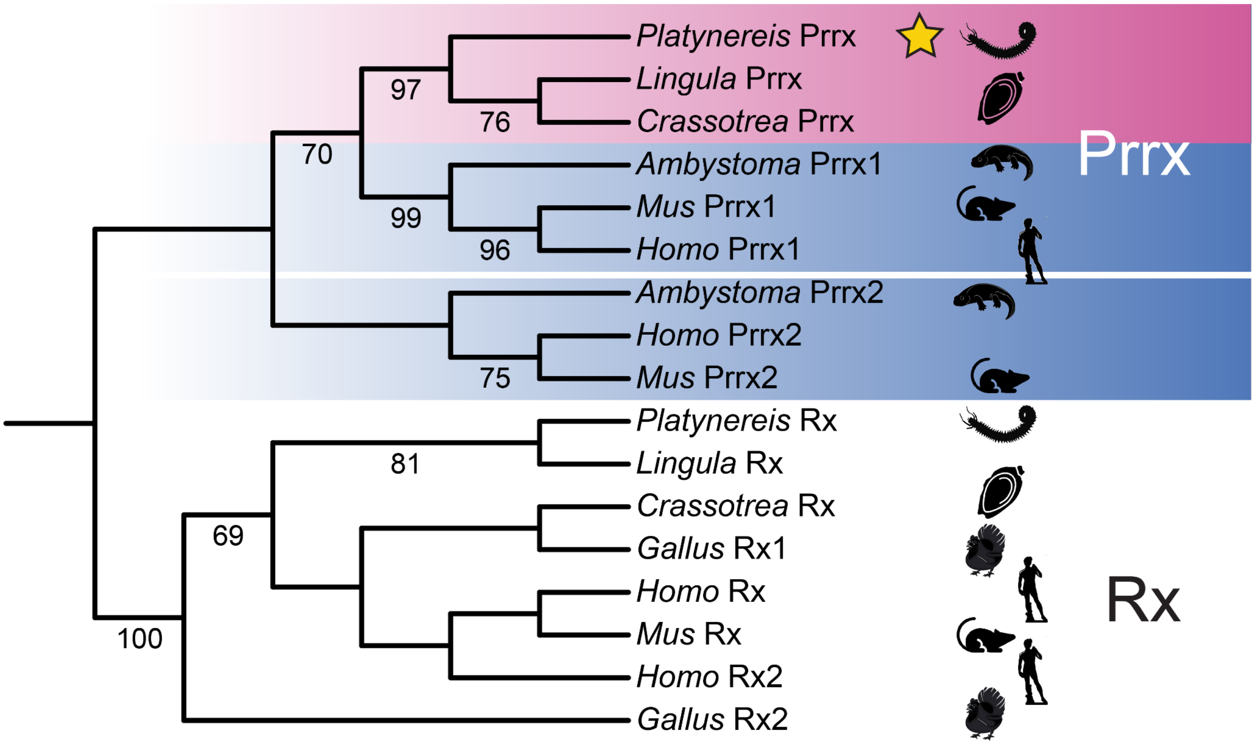

Supplementary Data 6 Figure 2. *Platynereis* Prrx is a homolog of vertebrate Prrx proteins. Sequence Identifiers: **Prrx proteins:** XGX14289 / PQ334003 (*Platynereis dumerilii*; this study); XP_013387302.1 (*Lingula anatina*), XP_011417364.1 (*Crassostrea gigas*), AMEX60DD201018450.2 (*Ambystoma mexicanum), NP_035257.1 (Mus musculus), NP_008833.1 (Homo sapiens), Prrx2: AMEX60DD201050342.16 (*Ambystoma mexicanum), NP_057391.1 (*Homo sapiens*), NP_033142.2 (*Mus musculus*); **Outgroup (Rx proteins):** XP_011417364.1 (*Mus musculus*), NP_038463.2, NP_001306003.2 (*Homo sapiens*), NP_001230653.1, NP_989435.2 (*Gallus gallus*), XP_023932426.1 (*Lingula anatina*), AAU20320.1 (*Platynereis dumerilii*), XP_011427710.2 (*Crassotrea gigas*). Bootstrap support (in %) is indicated below relevant nodes.

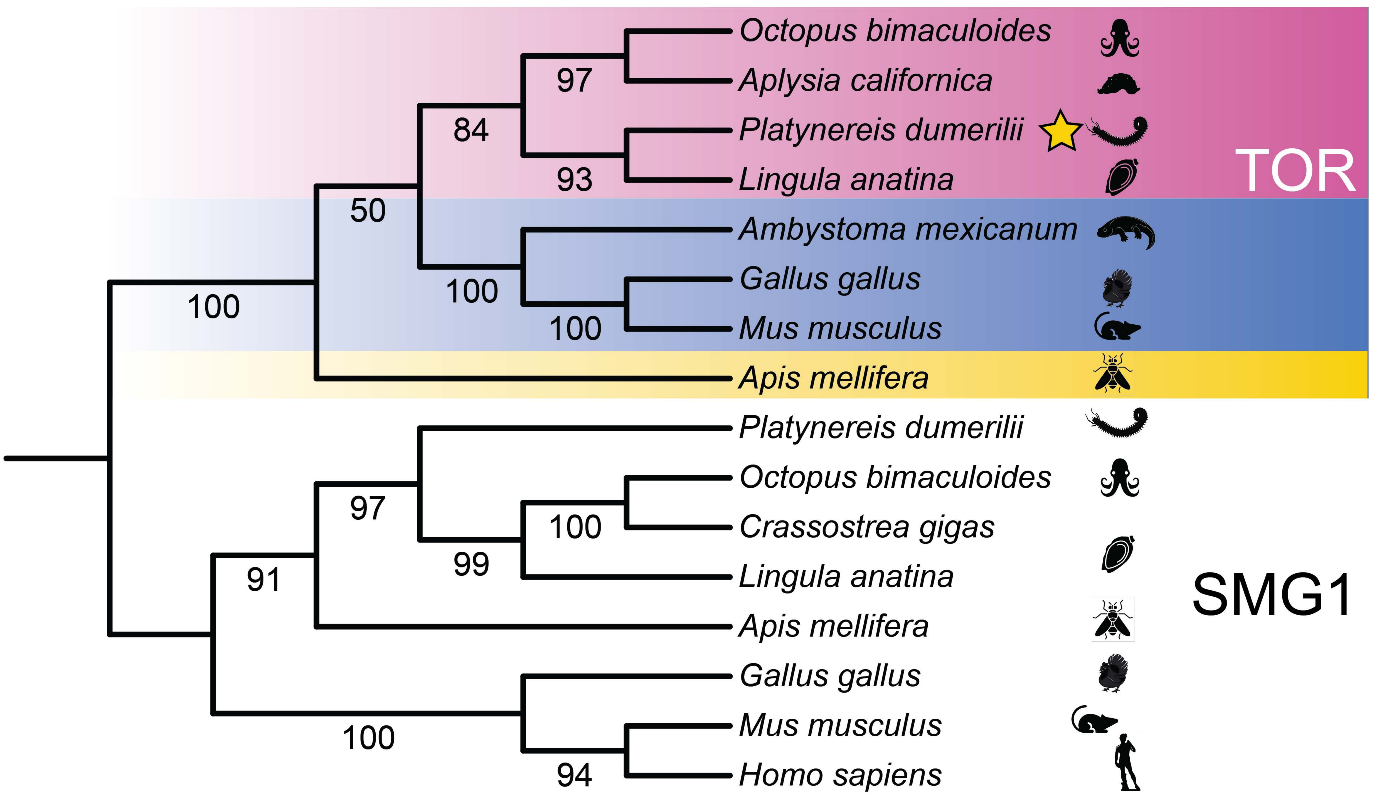

Supplementary Data 6 Figure 3. *Platynereis* possesses a TOR ortholog. Sequence Protein identifiers: **TOR:** AVY54328.1 (*Octopus vulgaris*), XP_005094248.1 (*Aplysia californica*), XGX14290 / PQ334004 (*Platynereis dumerilii;* this study), XP_013395747.1 (*Lingula anatina*), GB44905-PA (*Apis mellifera*), AMEX60DD301051133.3 (*Ambystoma mexicanum*), ENSGALP00000005274 (*Gallus gallus*), |Q9JLN9 (*Mus musculus*); **Outgroup (SMG1):** XGX14291 / PQ334005 (*Platynereis dumerilii*; this study), XP_052825533.1 (*Octopus bimaculoides*), XP_011454765.2 (*Crassostrea gigas*), XP_013417614.1 (*Lingula anatina*), KAG9429244.1 *(Apis mellifera)*, XP_040539861.1 (*Gallus gallus*), XP_006507741.1 (*Mus musculus*), NP_055907.3 (*Homo sapiens*). Bootstrap support (in %) is indicated below relevant nodes.
