# Supplementary material for: Molecular profiles, sources and lineage restrictions of stem cells in an annelid regeneration model
Source: Nat Commun. 2024 Nov 18;15:9882. doi: 10.1038/s41467-024-54041-3 (PMC11574210; doi:10.1038/s41467-024-54041-3)
Supplement: Supplementary file 10 — Supplementary Data 7 [file 41467_2024_54041_MOESM10_ESM.pdf]

Supplementary data file 7, transgenic clones, part A

Description of the transverse section scheme used for the mapping of transgenic clones

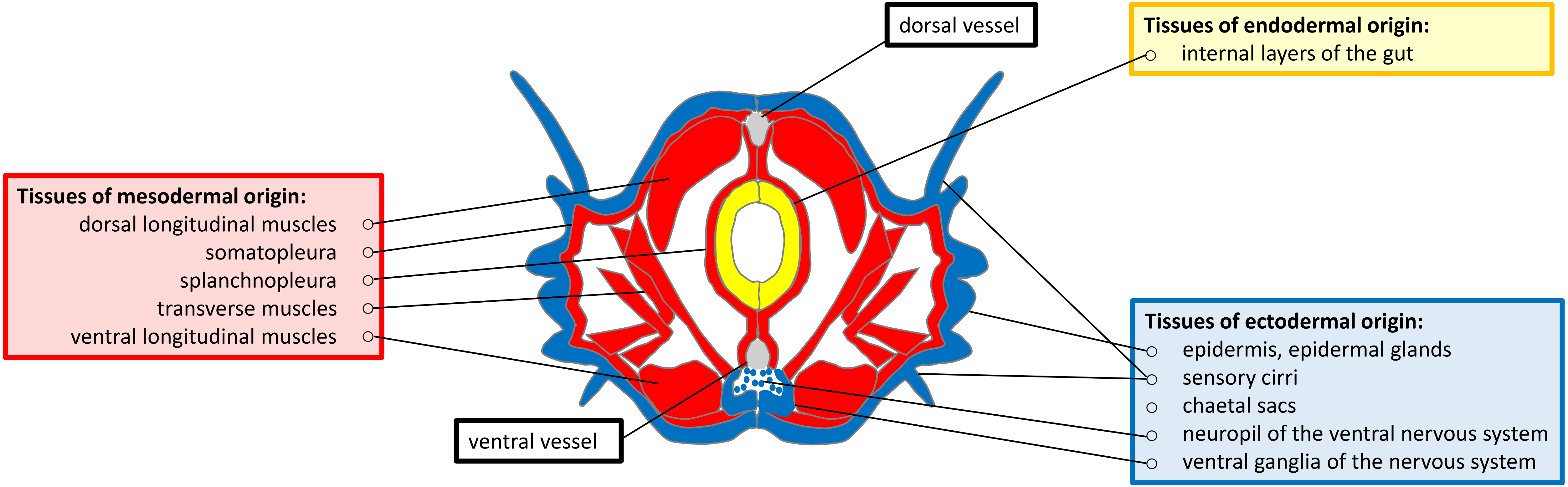

Transgenic tissues are highlighted in the germ layer color code — ectoderm, mesoderm, endoderm

Transgenic tissues displaying stochastic expression — ectoderm, mesoderm, endoderm

Non fluorescent tissues (or slightly autofluorescent) are in grey

Schematic frontal section used showing the locations of putative posterior stem cells

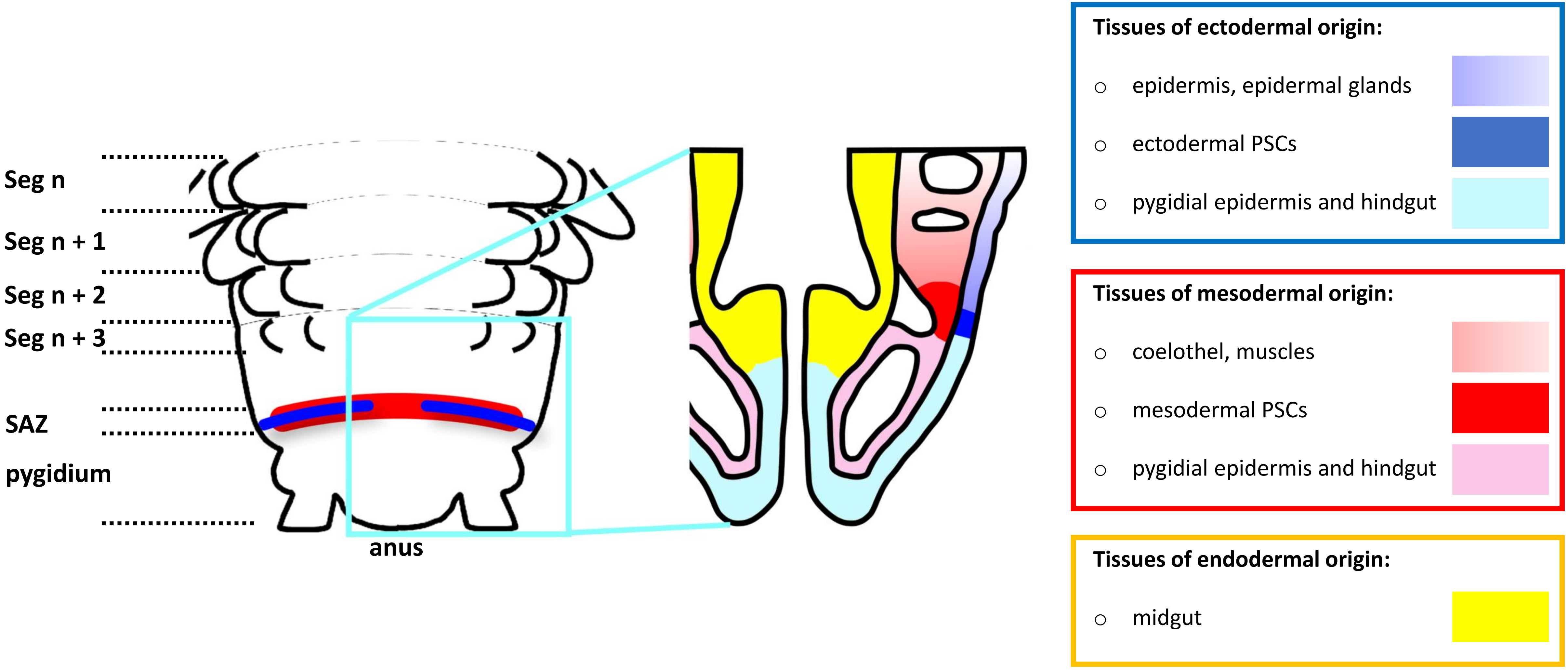

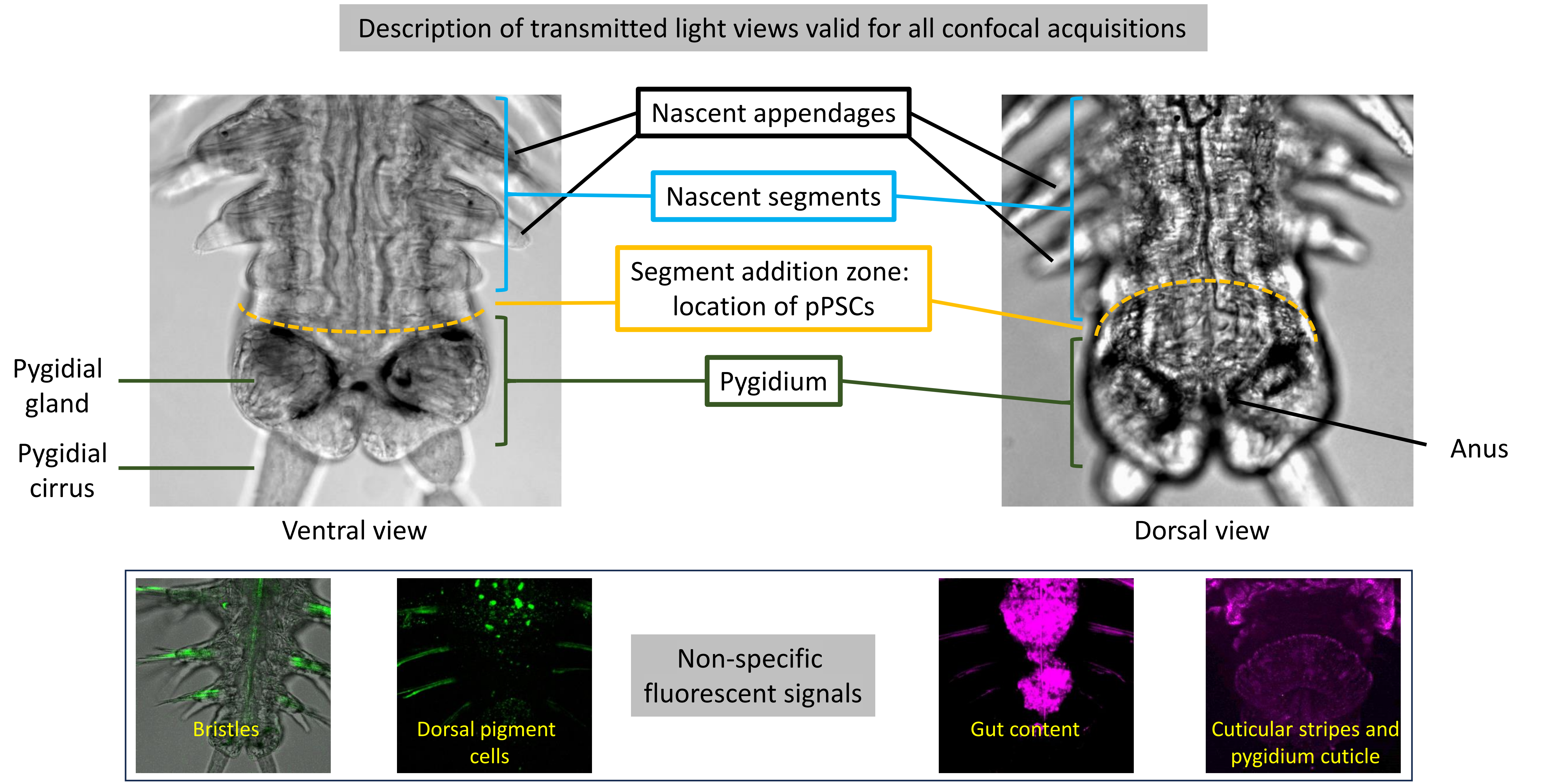
Description of transmitted light views valid for all confocal acquisitions
Nascent appendages
Nascent segments
Segment addition zone:
location of pPSCs
Pygidium
Pygidial
gland
Pygidial
cirrus
Anus
Ventral view
Dorsal view
Bristles
Dorsal pigment
cells
Non-specific
fluorescent signals
Gut content
Cuticular stripes and
pygidium cuticle

Supplementary data file 7, transgenic clones, part B

M01

membranes
nuclei

Full Z-projections

Frontal section
+ transverse scheme

Transgenic tissues :

- ectoderm
- mesoderm
- endoderm

ORIGINAL TAIL
(no regeneration)

No data

Accidentally amputated before 6 weeks

After 1st amputation-
regeneration

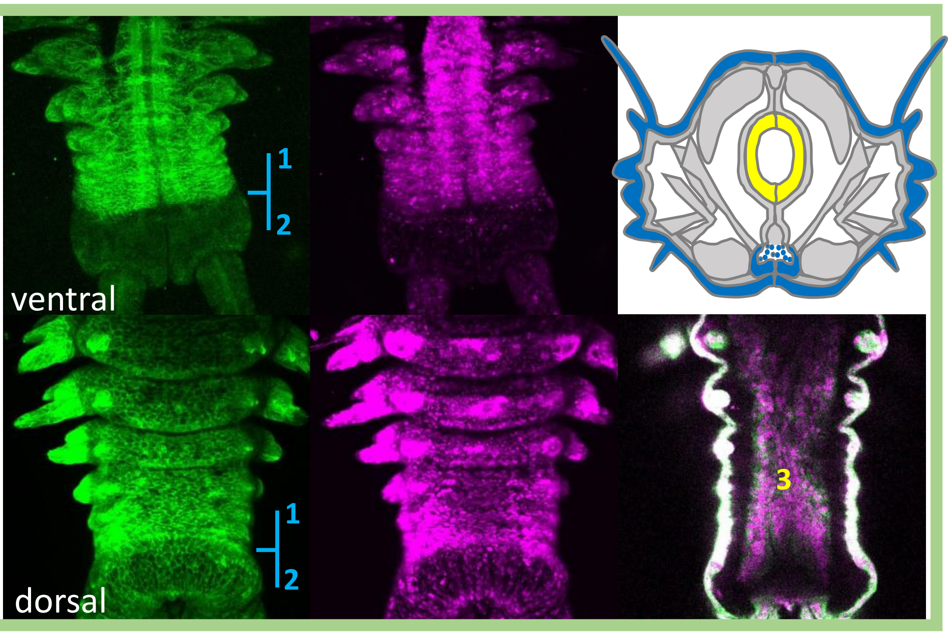

- **Segmental ectoderm 1**
- **Pygidium ectoderm, weak 2**
- **Gut endoderm 3**

After 2nd amputation-
regeneration

No ventral view

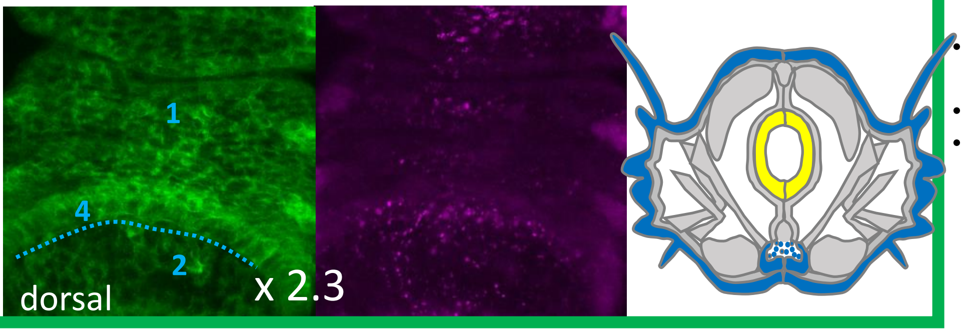

- **Segmental ectoderm 1**
- **Pygidium ectoderm, weaker 2**
- **Ectodermal PSCs? 4**
- **Gut endoderm**

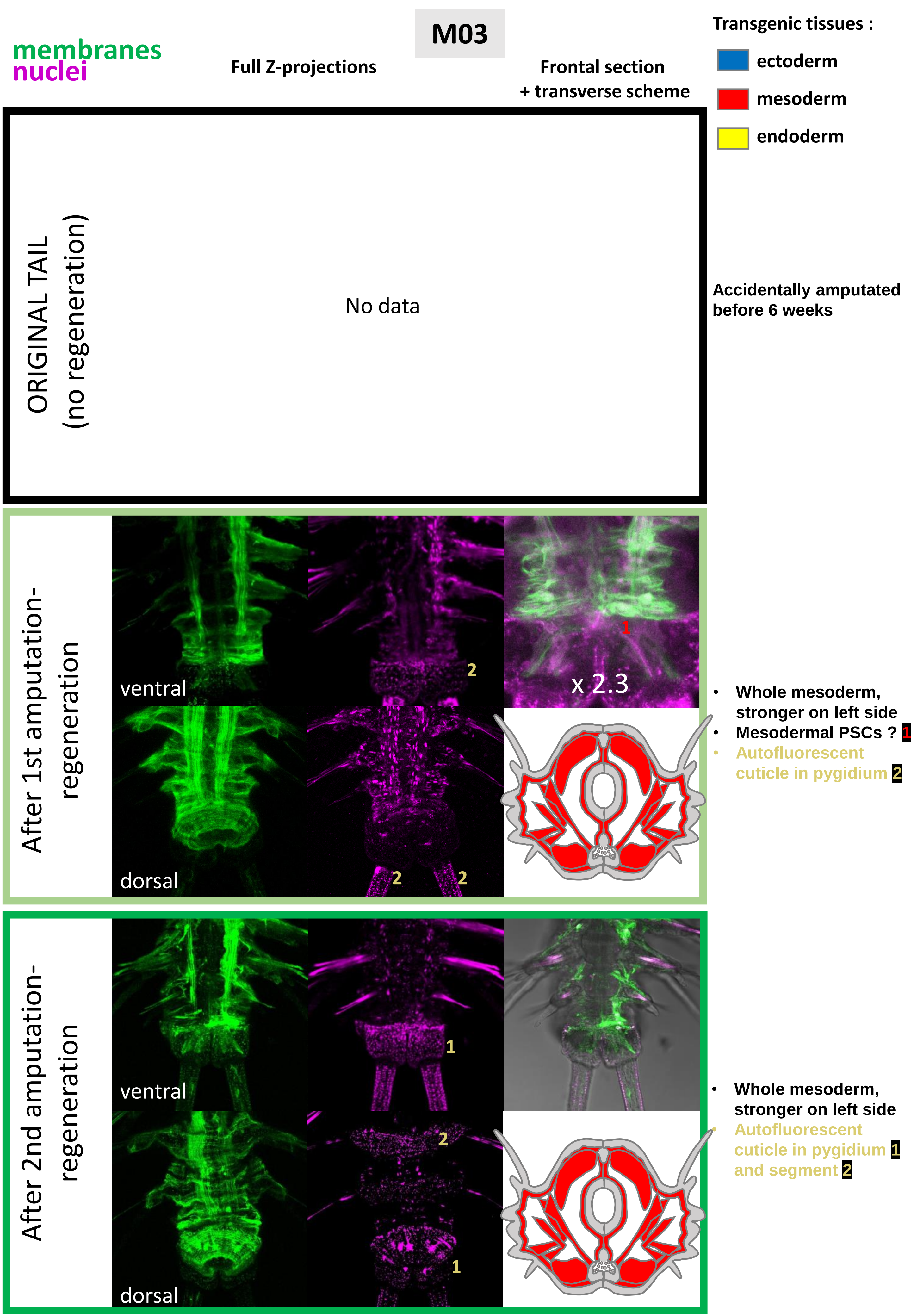
M03
membranes
nuclei
Full Z-projections
Frontal section
+ transverse scheme
Transgenic tissues :
ectoderm
mesoderm
endoderm
ORIGINAL TAIL
(no regeneration)
No data
Accidentally amputated before 6 weeks
After 1st amputation-regeneration
ventral
dorsal
x 2.3
1
2
2
2
• Whole mesoderm, stronger on left side
• Mesodermal PSCs ? 1
• Autofluorescent cuticle in pygidium 2
After 2nd amputation-regeneration
ventral
dorsal
1
2
1
• Whole mesoderm, stronger on left side
• Autofluorescent cuticle in pygidium 1 and segment 2

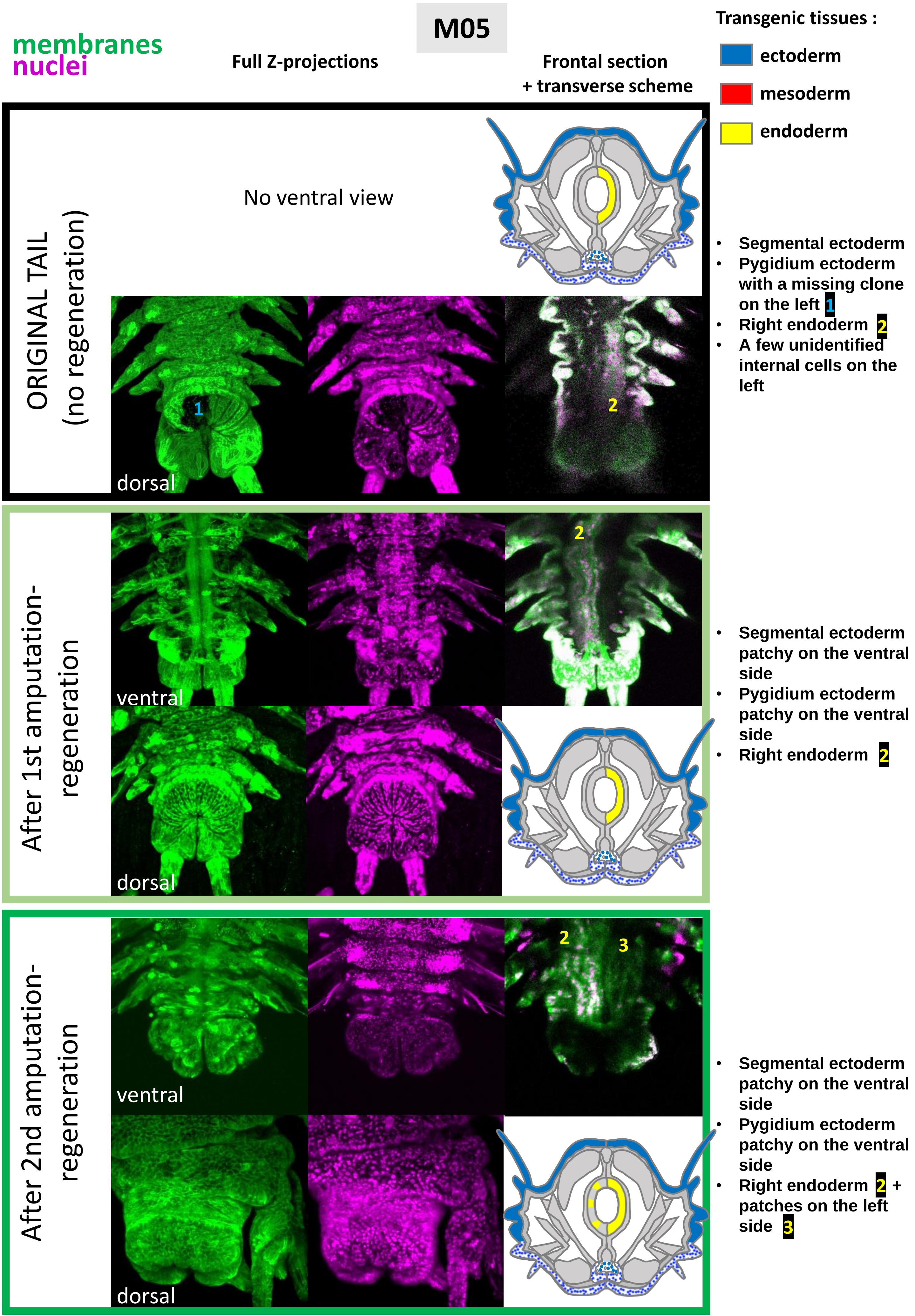
M05
membranes
nuclei
Full Z-projections
Frontal section
+ transverse scheme
Transgenic tissues :
ectoderm
mesoderm
endoderm
ORIGINAL TAIL
(no regeneration)
No ventral view
1
2
dorsal
• Segmental ectoderm
• Pygidium ectoderm with a missing clone on the left 1
• Right endoderm 2
• A few unidentified internal cells on the left
After 1st amputation-regeneration
2
ventral
dorsal
• Segmental ectoderm patchy on the ventral side
• Pygidium ectoderm patchy on the ventral side
• Right endoderm 2
After 2nd amputation-regeneration
2
3
ventral
dorsal
• Segmental ectoderm patchy on the ventral side
• Pygidium ectoderm patchy on the ventral side
• Right endoderm 2 + patches on the left side 3

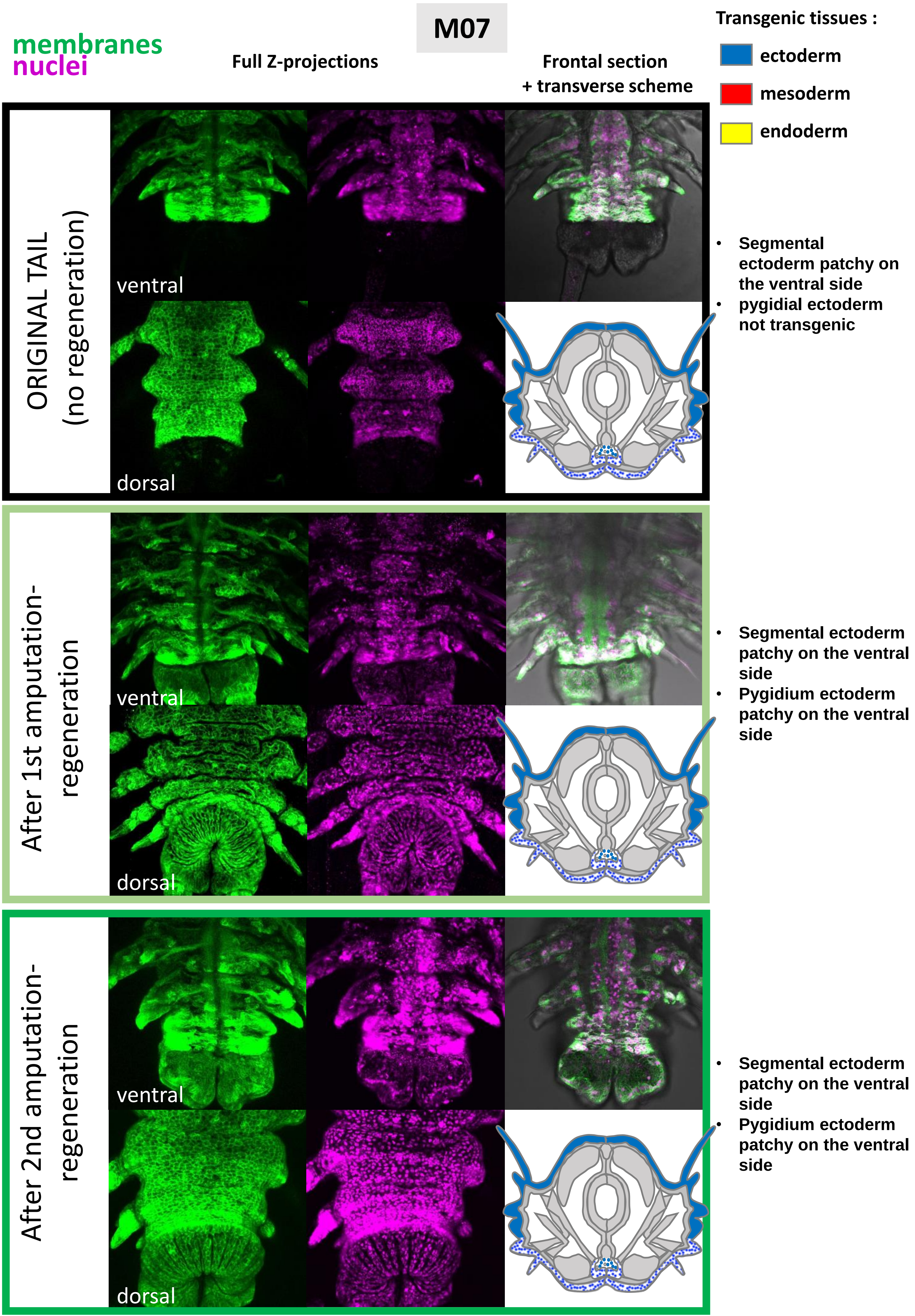
M07
membranes
nuclei
Full Z-projections
Frontal section
+ transverse scheme
Transgenic tissues :
ectoderm
mesoderm
endoderm
ORIGINAL TAIL
(no regeneration)
ventral
dorsal
• Segmental ectoderm patchy on the ventral side
• pygidial ectoderm not transgenic
After 1st amputation-regeneration
ventral
dorsal
• Segmental ectoderm patchy on the ventral side
• Pygidium ectoderm patchy on the ventral side
After 2nd amputation-regeneration
ventral
dorsal
• Segmental ectoderm patchy on the ventral side
• Pygidium ectoderm patchy on the ventral side

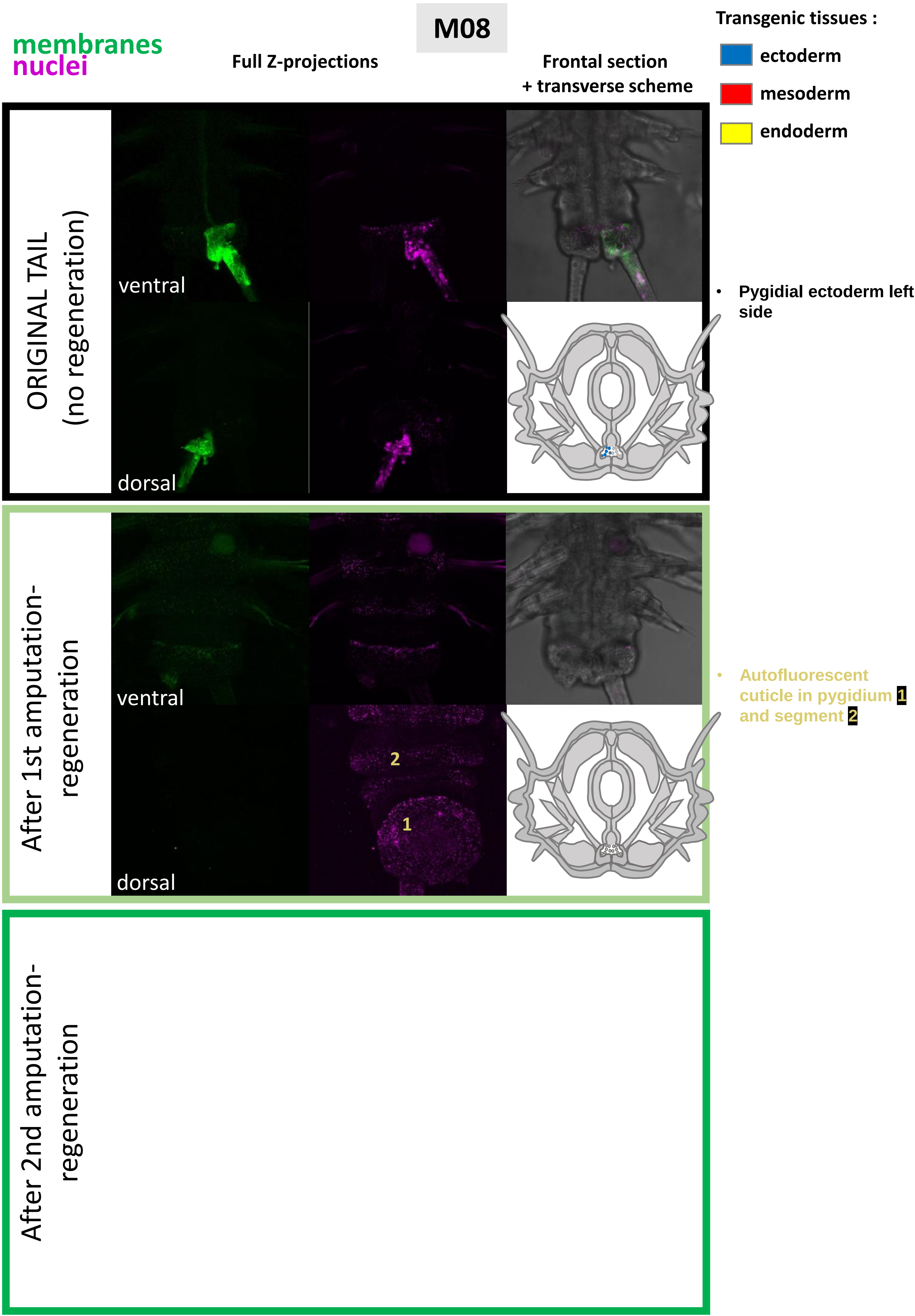
M08
membranes
nuclei
Full Z-projections
Frontal section
+ transverse scheme
Transgenic tissues :
ectoderm
mesoderm
endoderm
ORIGINAL TAIL
(no regeneration)
ventral
dorsal
• Pygidial ectoderm left side
After 1st amputation-
regeneration
ventral
dorsal
2
1
• Autofluorescent cuticle in pygidium 1 and segment 2
After 2nd amputation-
regeneration

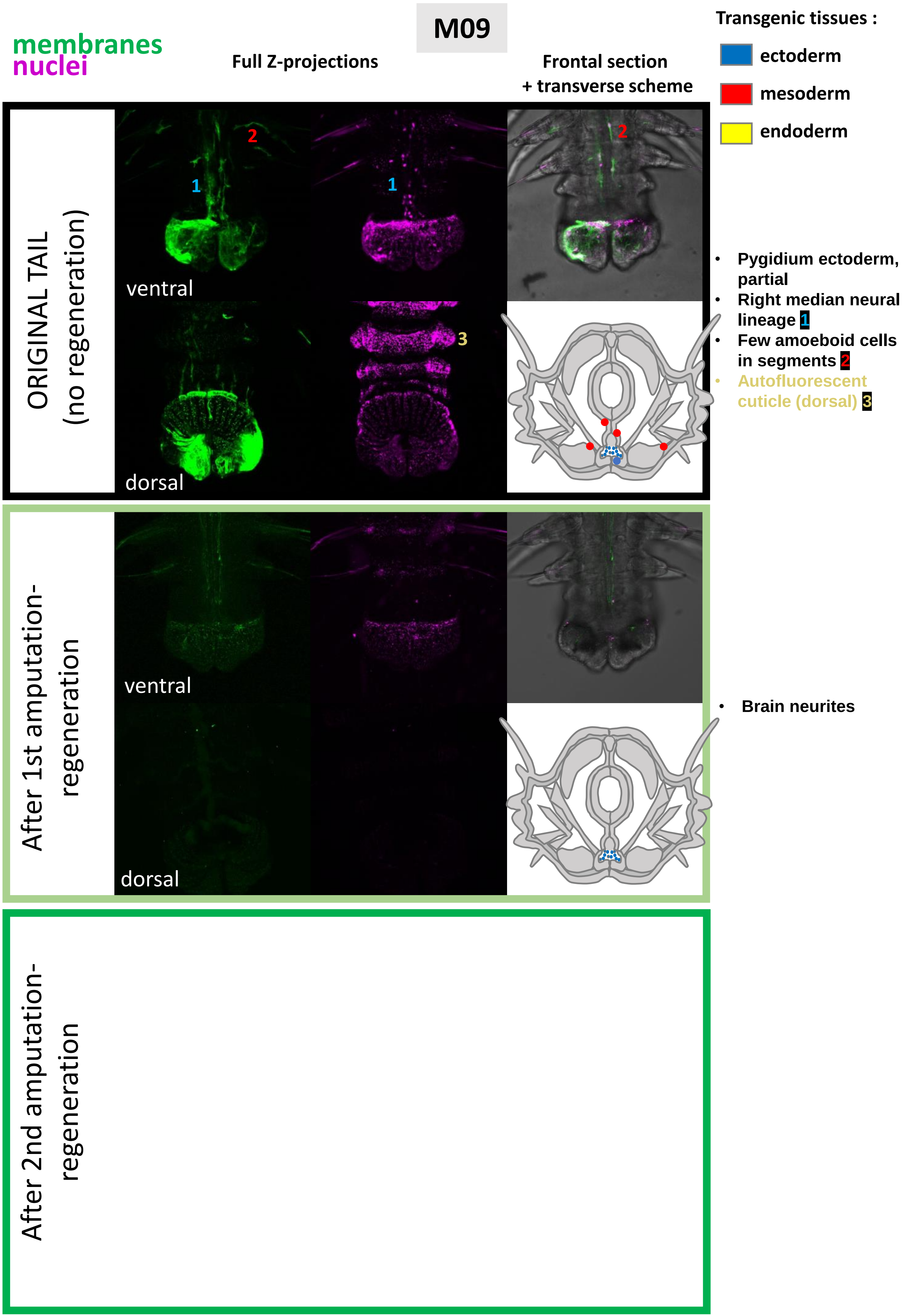
M09
membranes
nuclei
Full Z-projections
Frontal section
+ transverse scheme
Transgenic tissues :
ectoderm
mesoderm
endoderm
ORIGINAL TAIL
(no regeneration)
ventral
dorsal
1
2
3
Pygidium ectoderm, partial
Right median neural lineage 1
Few amoeboid cells in segments 2
Autofluorescent cuticle (dorsal) 3
After 1st amputation-regeneration
ventral
dorsal
Brain neurites
After 2nd amputation-regeneration

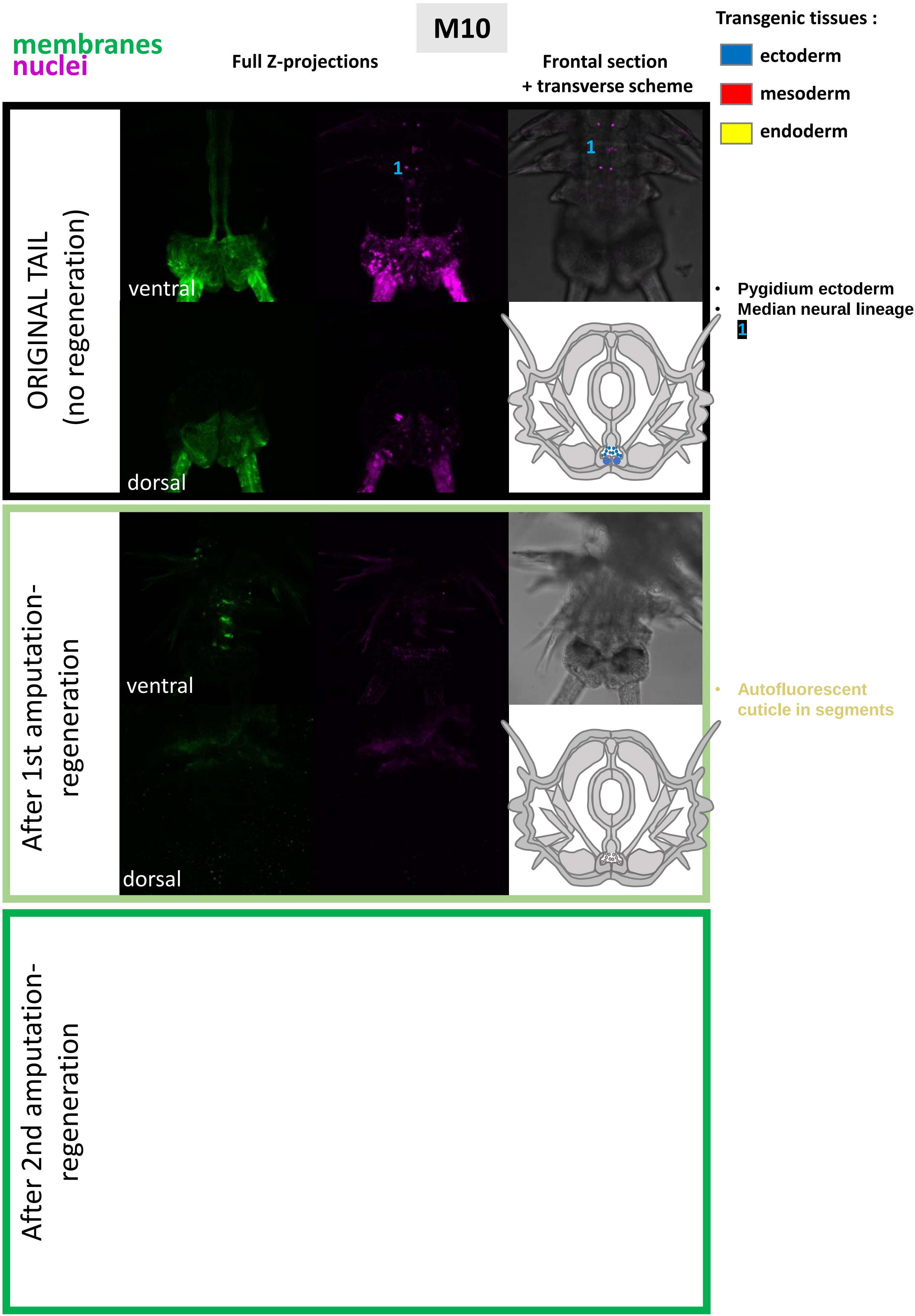
M10
membranes
nuclei
Full Z-projections
Frontal section
+ transverse scheme
Transgenic tissues :
ectoderm
mesoderm
endoderm
ORIGINAL TAIL
(no regeneration)
ventral
dorsal
1
Pygidium ectoderm
Median neural lineage
1
After 1st amputation-
regeneration
Autofluorescent
cuticle in segments
After 2nd amputation-
regeneration

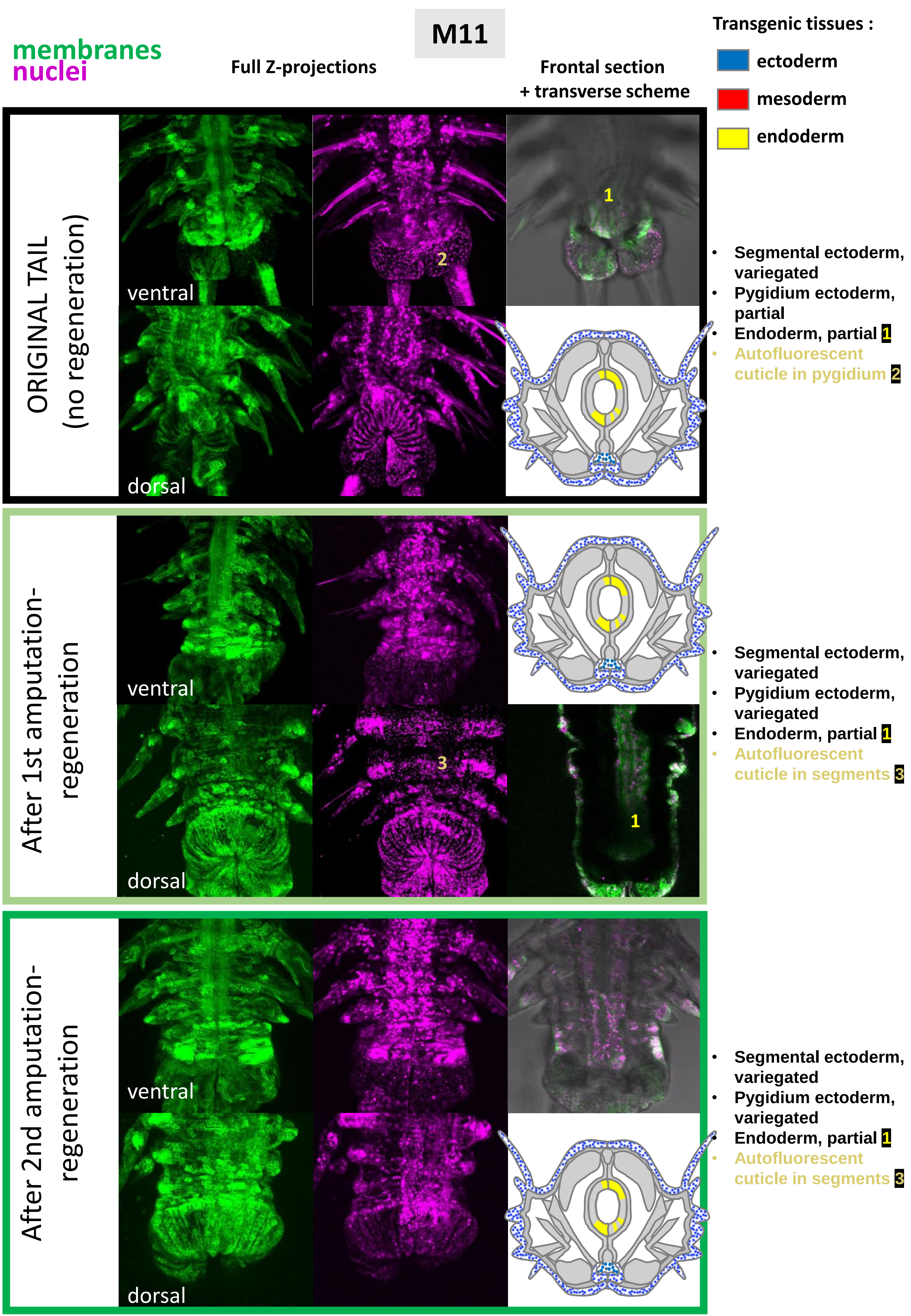

M11
membranes
nuclei
Full Z-projections
Frontal section
+ transverse scheme
Transgenic tissues :
ectoderm
mesoderm
endoderm
ORIGINAL TAIL
(no regeneration)
ventral
dorsal
1
2
Segmental ectoderm, variegated
Pygidium ectoderm, partial
Endoderm, partial 1
Autofluorescent cuticle in pygidium 2
After 1st amputation-regeneration
ventral
dorsal
3
1
Segmental ectoderm, variegated
Pygidium ectoderm, variegated
Endoderm, partial 1
Autofluorescent cuticle in segments 3
After 2nd amputation-regeneration
ventral
dorsal
Segmental ectoderm, variegated
Pygidium ectoderm, variegated
Endoderm, partial 1
Autofluorescent cuticle in segments 3

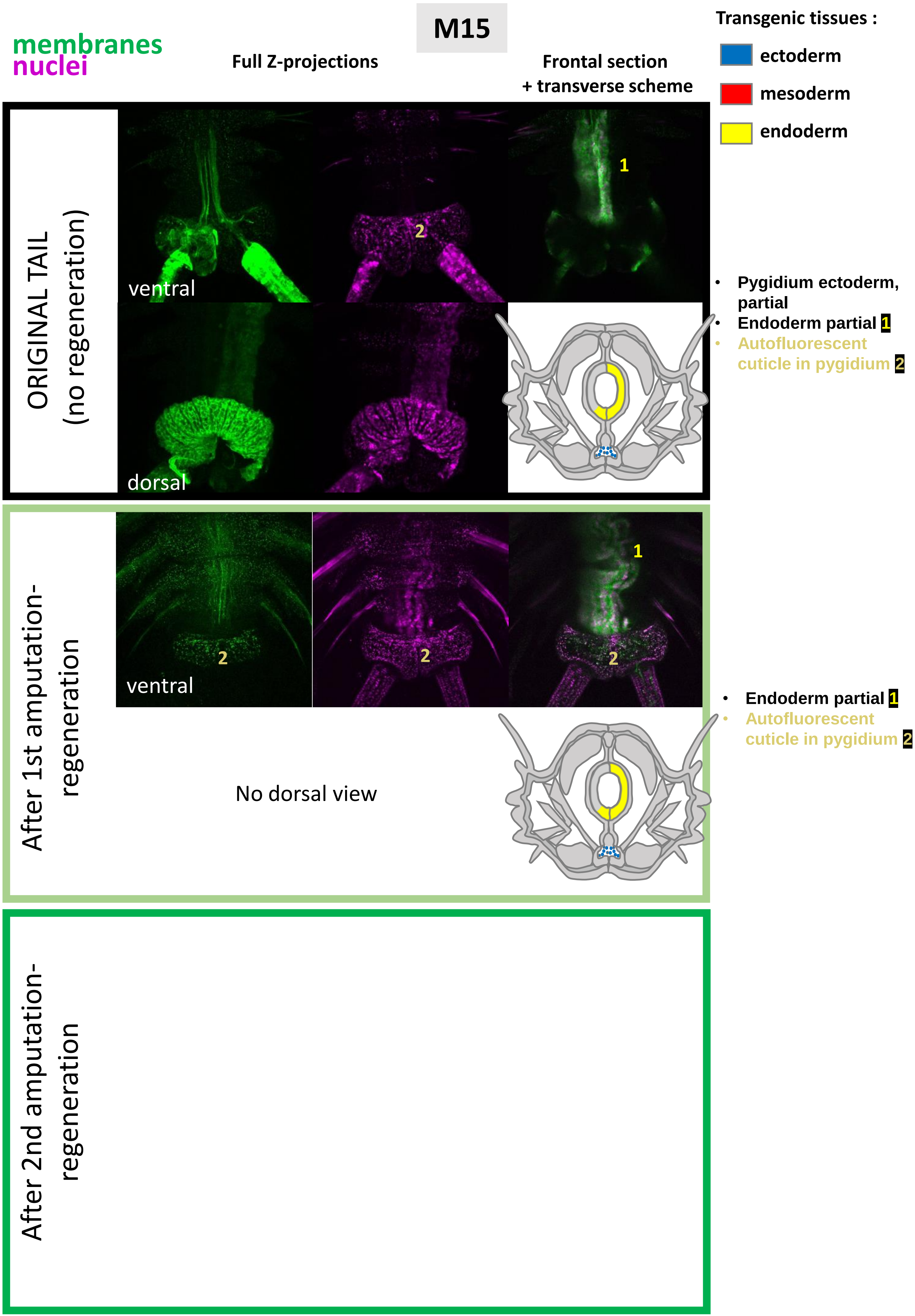
M15
membranes
nuclei
Full Z-projections
Frontal section
+ transverse scheme
Transgenic tissues :
ectoderm
mesoderm
endoderm
ORIGINAL TAIL
(no regeneration)
ventral
dorsal
1
2
• Pygidium ectoderm, partial
• Endoderm partial 1
• Autofluorescent cuticle in pygidium 2
After 1st amputation-regeneration
ventral
No dorsal view
• Endoderm partial 1
• Autofluorescent cuticle in pygidium 2
After 2nd amputation-regeneration

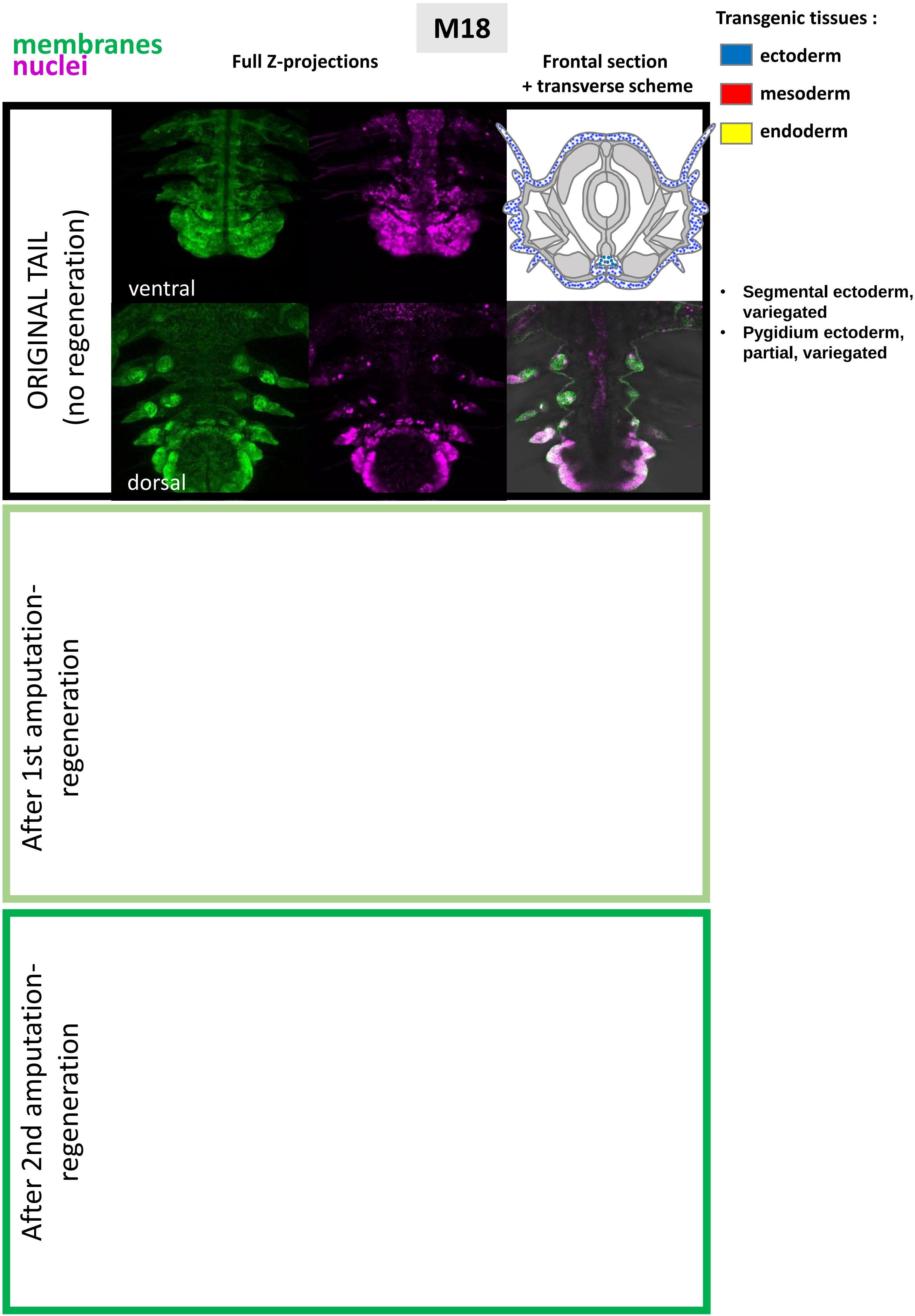

M18
membranes
nuclei
Full Z-projections
Frontal section
+ transverse scheme
Transgenic tissues :
ectoderm
mesoderm
endoderm
ORIGINAL TAIL
(no regeneration)
ventral
dorsal
• Segmental ectoderm, variegated
• Pygidium ectoderm, partial, variegated
After 1st amputation-regeneration
After 2nd amputation-regeneration

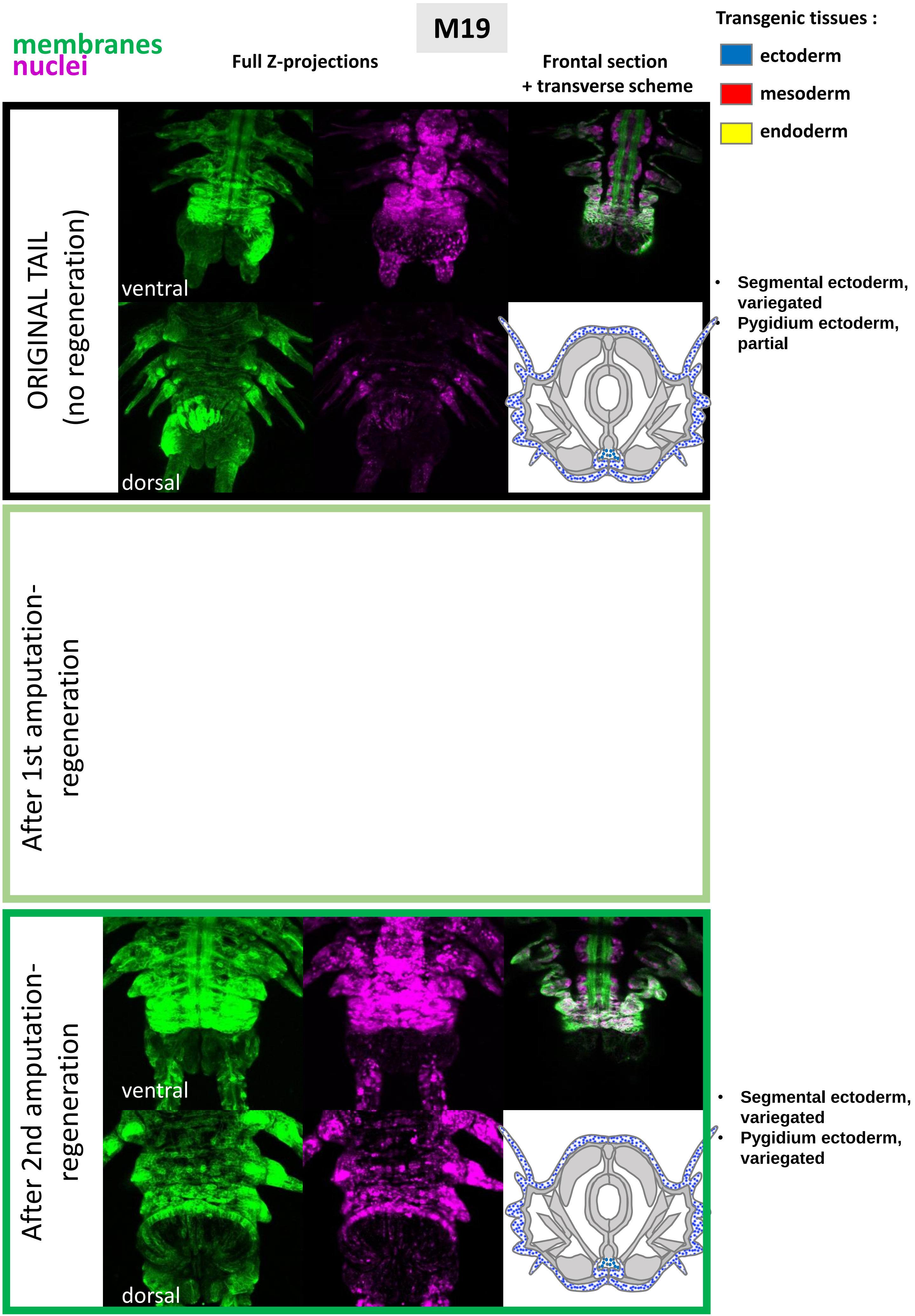
M19
membranes
nuclei
Full Z-projections
Frontal section
+ transverse scheme
Transgenic tissues :
ectoderm
mesoderm
endoderm
ORIGINAL TAIL
(no regeneration)
ventral
dorsal
• Segmental ectoderm, variegated
• Pygidium ectoderm, partial
After 1st amputation-regeneration
After 2nd amputation-regeneration
ventral
dorsal
• Segmental ectoderm, variegated
• Pygidium ectoderm, variegated

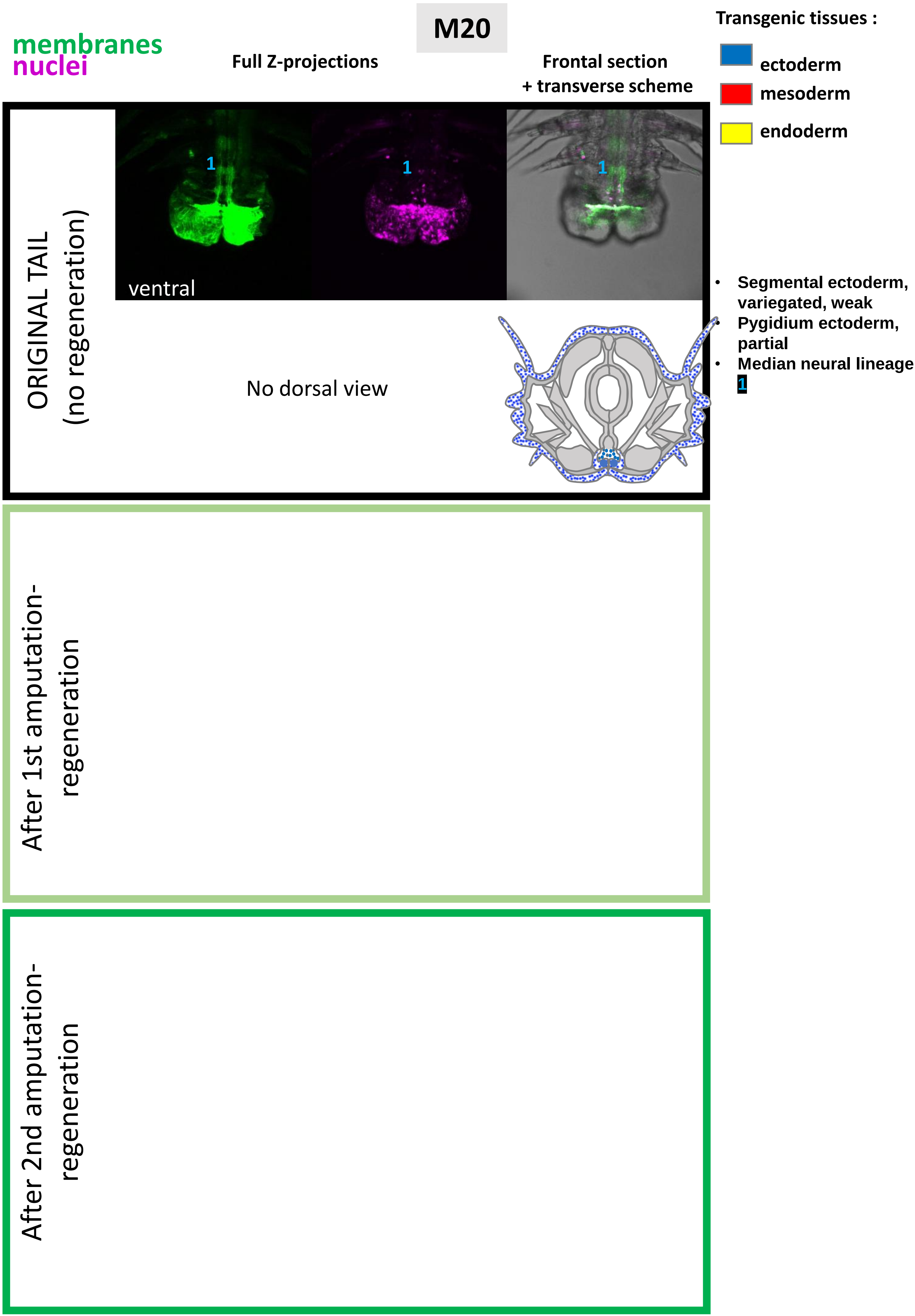
M20
membranes
nuclei
Full Z-projections
Frontal section
+ transverse scheme
Transgenic tissues :
ectoderm
mesoderm
endoderm
ORIGINAL TAIL
(no regeneration)
1
1
1
ventral
No dorsal view
Segmental ectoderm, variegated, weak
Pygidium ectoderm, partial
Median neural lineage
1
After 1st amputation-regeneration
After 2nd amputation-regeneration

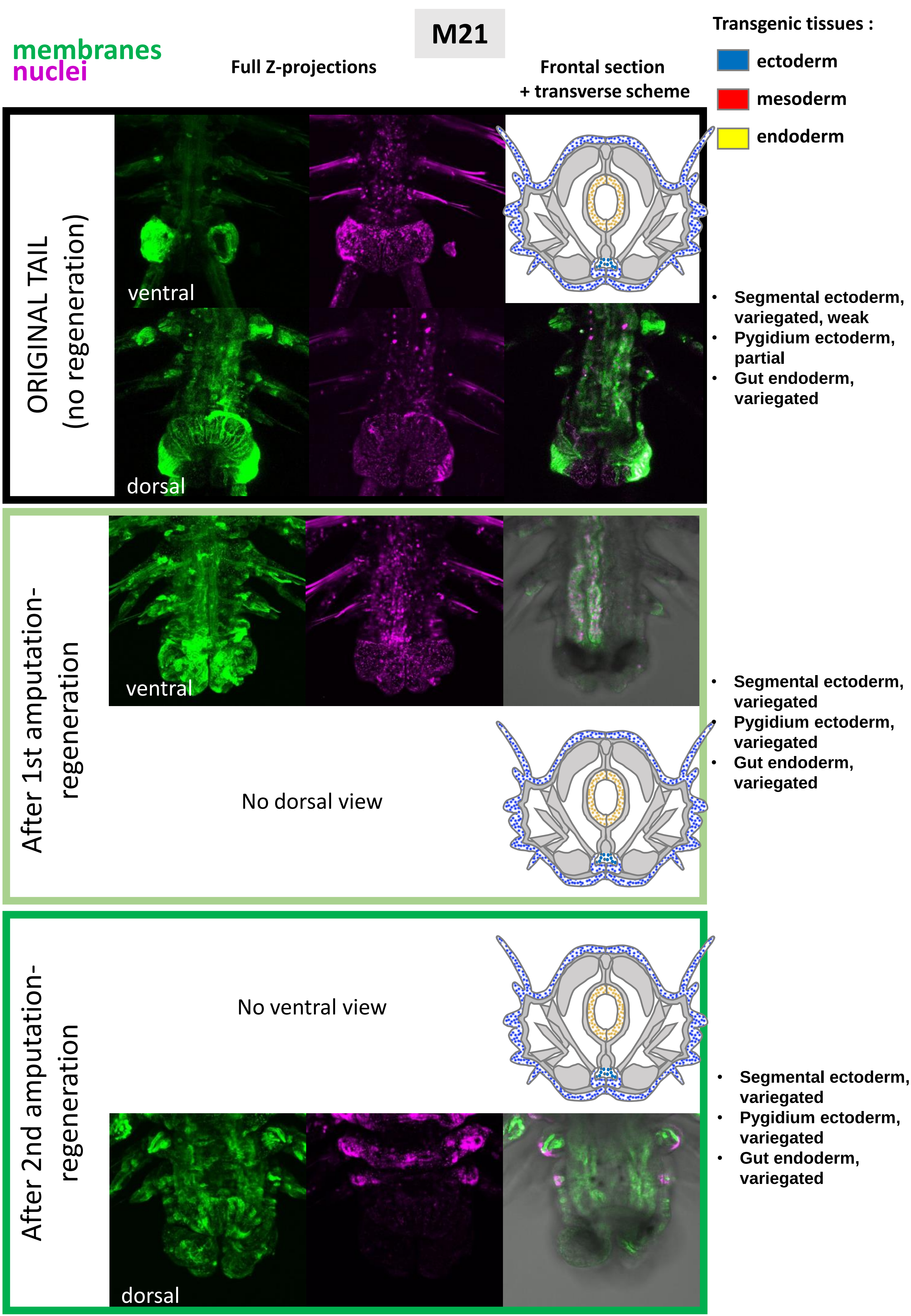
M21
membranes
nuclei
Full Z-projections
Frontal section
+ transverse scheme
Transgenic tissues :
ectoderm
mesoderm
endoderm
ORIGINAL TAIL
(no regeneration)
ventral
dorsal
• Segmental ectoderm, variegated, weak
• Pygidium ectoderm, partial
• Gut endoderm, variegated
After 1st amputation-regeneration
ventral
No dorsal view
• Segmental ectoderm, variegated
• Pygidium ectoderm, variegated
• Gut endoderm, variegated
After 2nd amputation-regeneration
No ventral view
dorsal
• Segmental ectoderm, variegated
• Pygidium ectoderm, variegated
• Gut endoderm, variegated

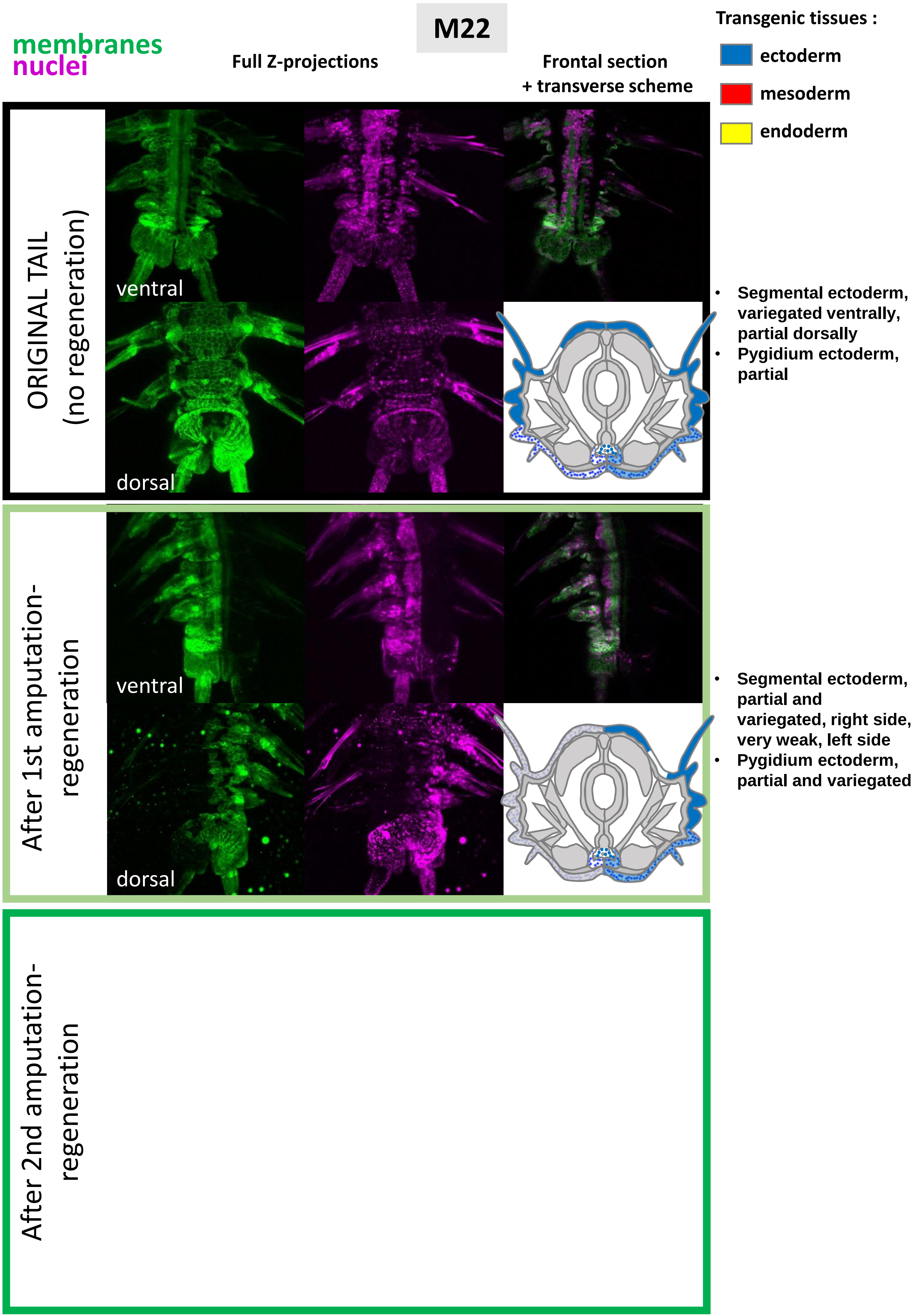
M22
membranes
nuclei
Full Z-projections
Frontal section
+ transverse scheme
Transgenic tissues :
ectoderm
mesoderm
endoderm
ORIGINAL TAIL
(no regeneration)
ventral
dorsal
• Segmental ectoderm, variegated ventrally, partial dorsally
• Pygidium ectoderm, partial
After 1st amputation-regeneration
ventral
dorsal
• Segmental ectoderm, partial and variegated, right side, very weak, left side
• Pygidium ectoderm, partial and variegated
After 2nd amputation-regeneration

membranes
nuclei

M23

Full Z-projections

Frontal section + transverse scheme

Transgenic tissues :

- ectoderm
- mesoderm
- endoderm

ORIGINAL TAIL (no regeneration)

No ventral view

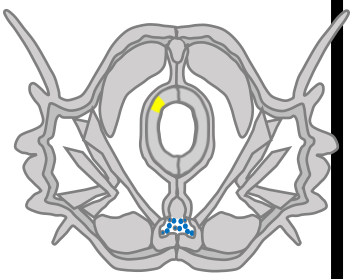

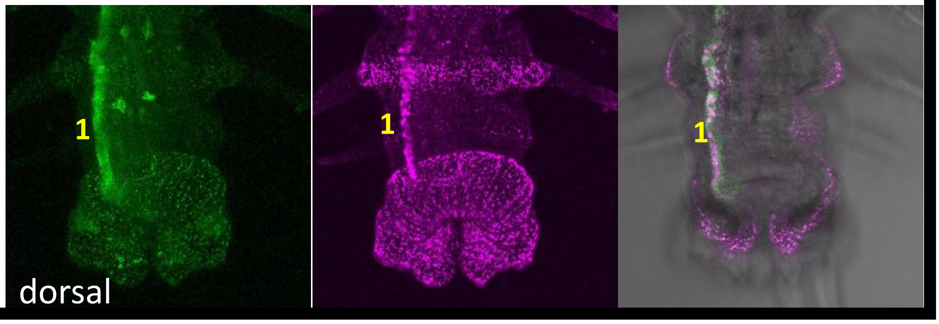

- **Gut endoderm, partial**
1

After 1st amputation-regeneration

After 2nd amputation-regeneration

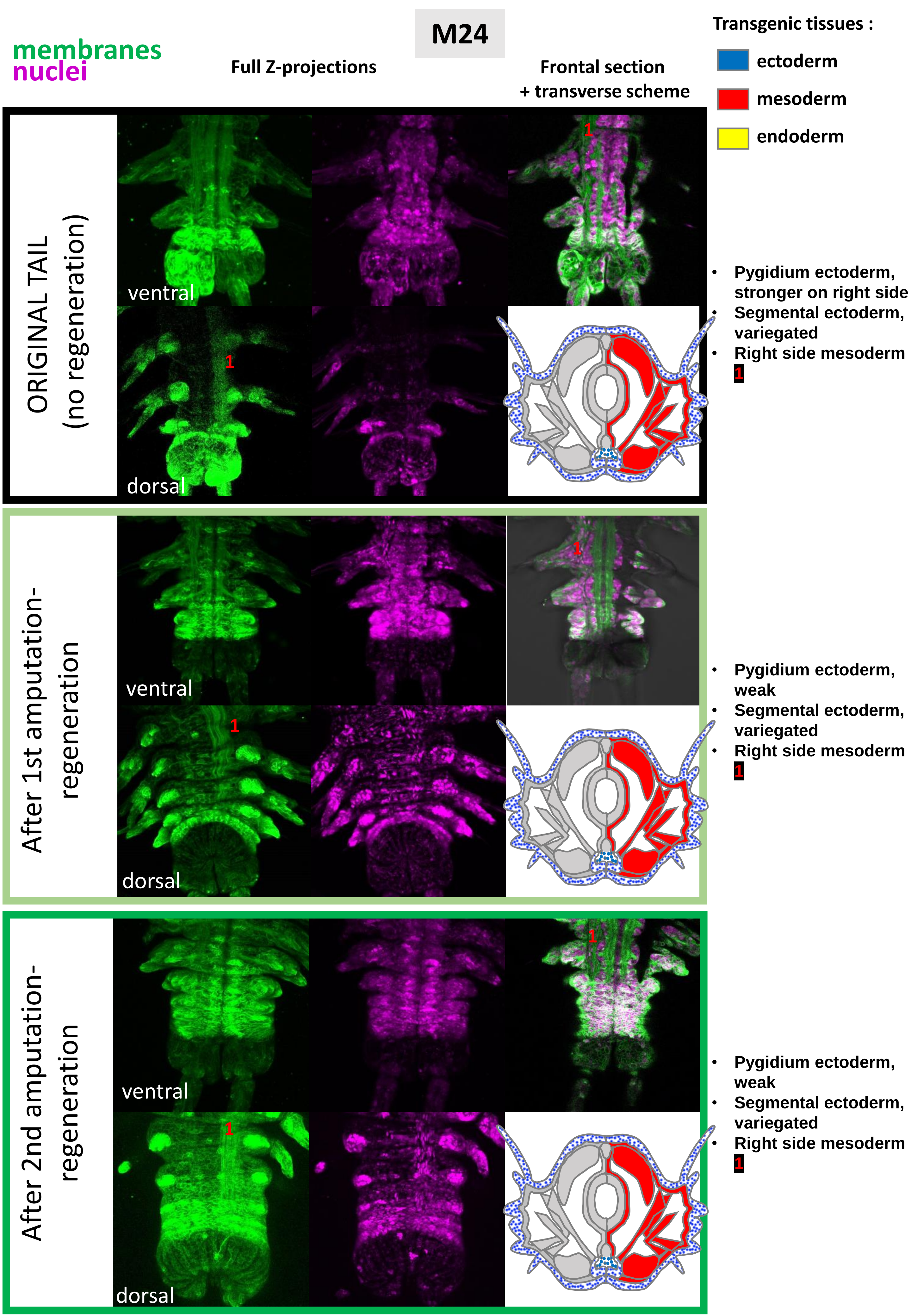
M24
membranes
nuclei
Full Z-projections
Frontal section
+ transverse scheme
Transgenic tissues :
ectoderm
mesoderm
endoderm
ORIGINAL TAIL
(no regeneration)
ventral
dorsal
1
Pygidium ectoderm, stronger on right side
Segmental ectoderm, variegated
Right side mesoderm
After 1st amputation-regeneration
ventral
dorsal
Pygidium ectoderm, weak
Segmental ectoderm, variegated
Right side mesoderm
After 2nd amputation-regeneration
ventral
dorsal
Pygidium ectoderm, weak
Segmental ectoderm, variegated
Right side mesoderm

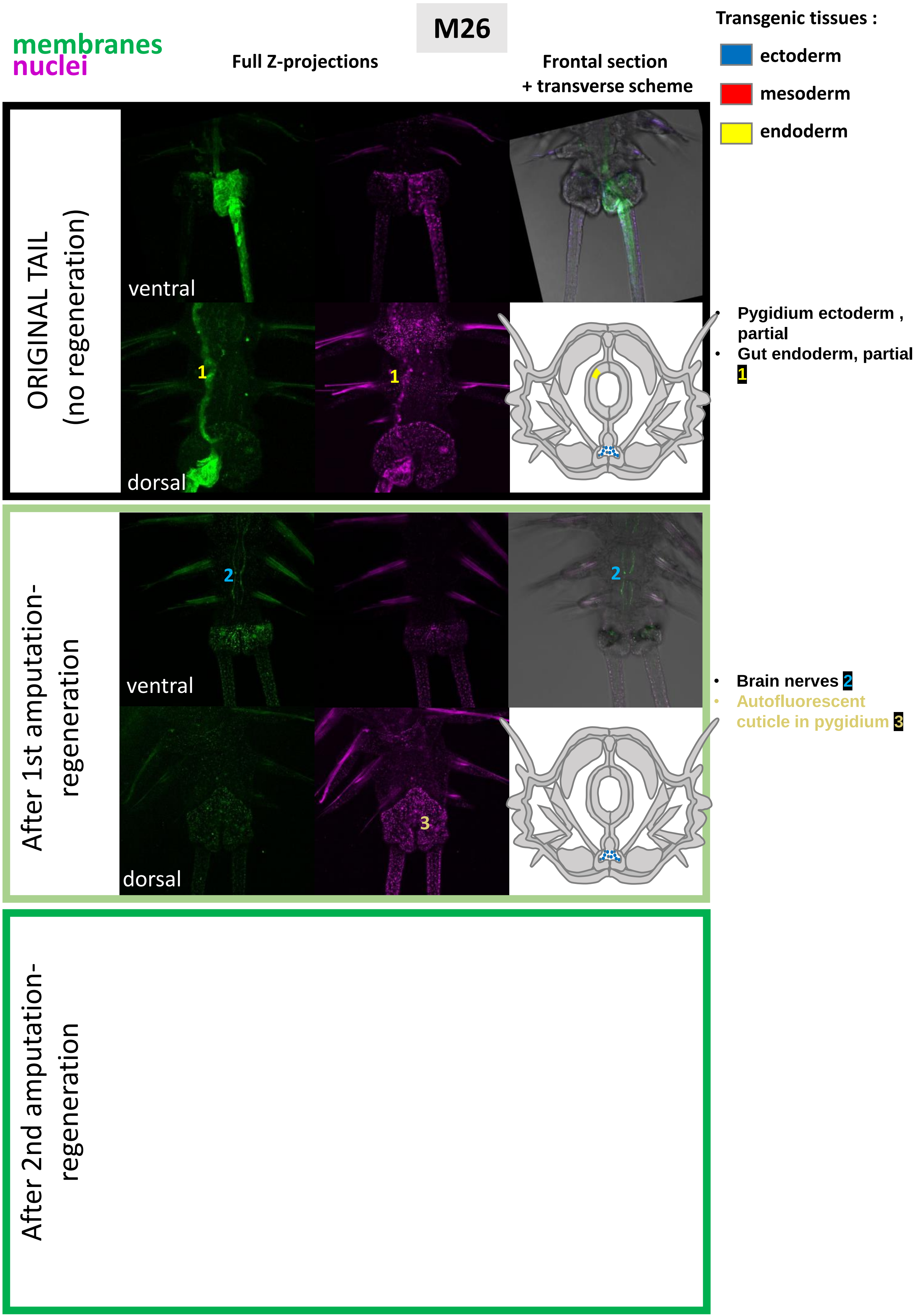
M26
membranes
nuclei
Full Z-projections
Frontal section
+ transverse scheme
Transgenic tissues :
ectoderm
mesoderm
endoderm
ORIGINAL TAIL
(no regeneration)
ventral
dorsal
1
1
• Pygidium ectoderm , partial
• Gut endoderm, partial 1
After 1st amputation-regeneration
2
2
ventral
dorsal
3
• Brain nerves 2
• Autofluorescent cuticle in pygidium 3
After 2nd amputation-regeneration

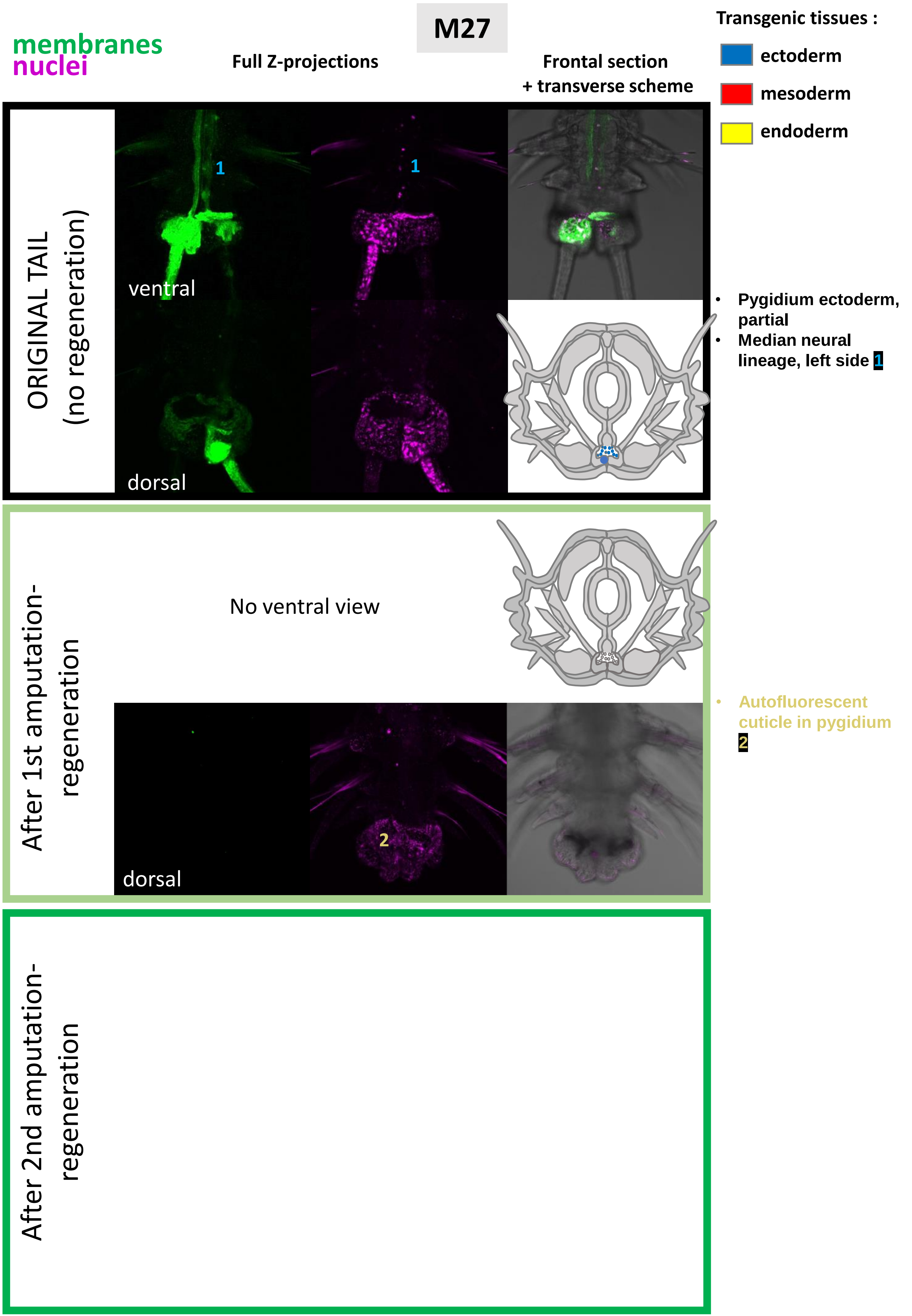
M27
membranes
nuclei
Full Z-projections
Frontal section
+ transverse scheme
Transgenic tissues :
ectoderm
mesoderm
endoderm
ORIGINAL TAIL
(no regeneration)
1
1
ventral
dorsal
Pygidium ectoderm, partial
Median neural lineage, left side 1
After 1st amputation-regeneration
No ventral view
2
dorsal
Autofluorescent cuticle in pygidium 2
After 2nd amputation-regeneration

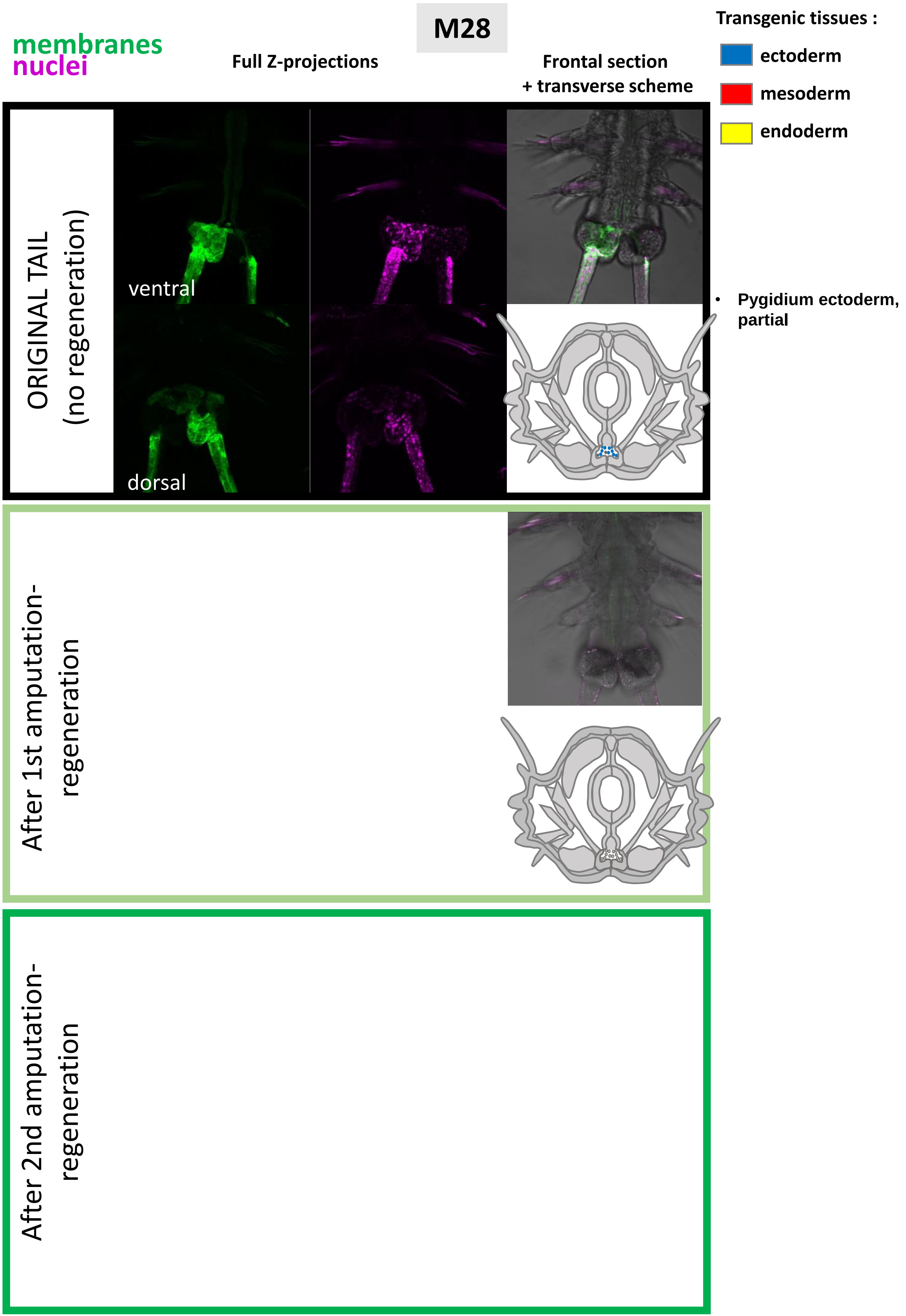
M28
membranes
nuclei
Full Z-projections
Frontal section
+ transverse scheme
Transgenic tissues :
ectoderm
mesoderm
endoderm
ORIGINAL TAIL
(no regeneration)
ventral
dorsal
• Pygidium ectoderm, partial
After 1st amputation-
regeneration
After 2nd amputation-
regeneration

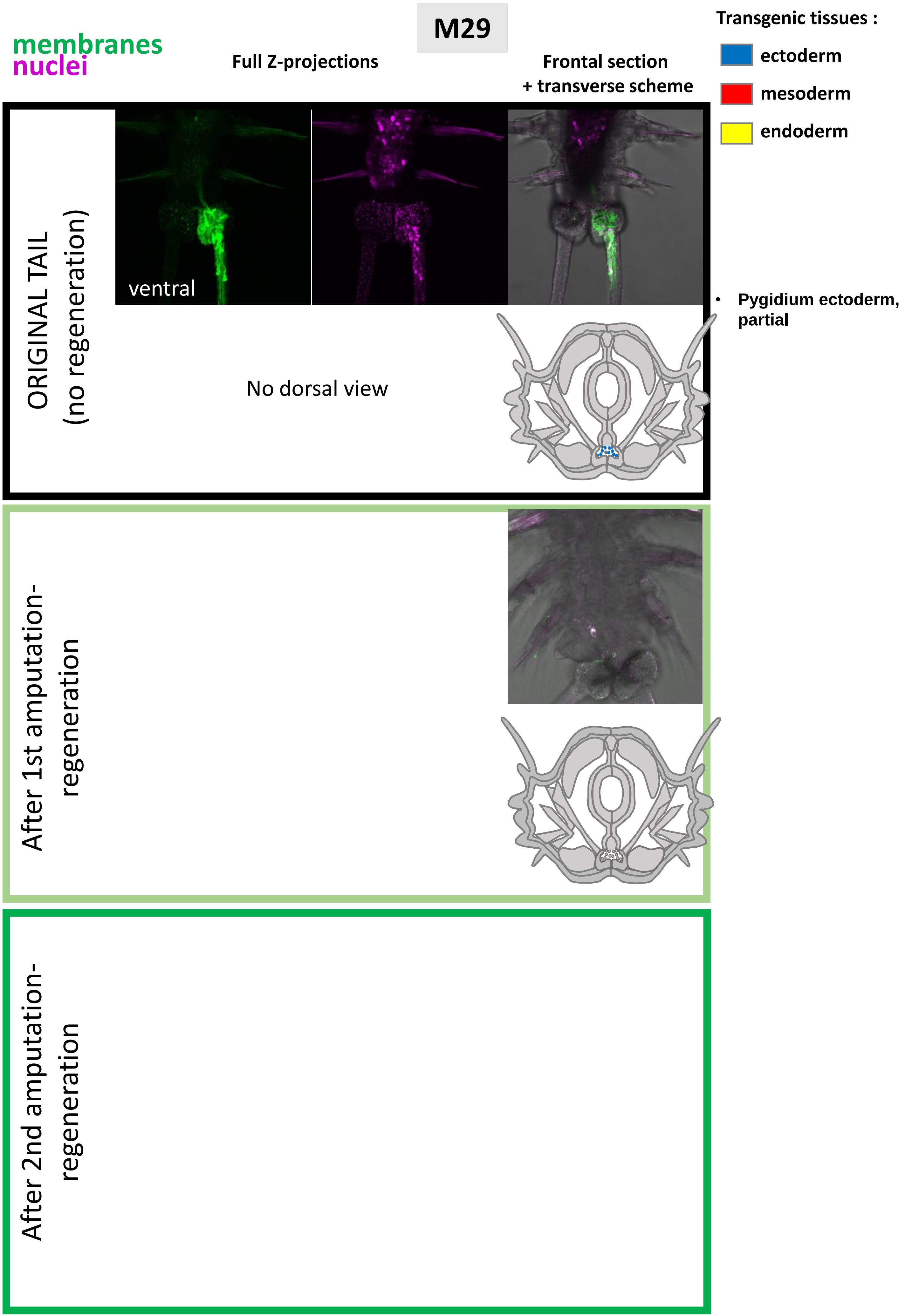
M29
membranes
nuclei
Full Z-projections
Frontal section
+ transverse scheme
Transgenic tissues :
ectoderm
mesoderm
endoderm
ORIGINAL TAIL
(no regeneration)
ventral
No dorsal view
• Pygidium ectoderm, partial
After 1st amputation-regeneration
After 2nd amputation-regeneration

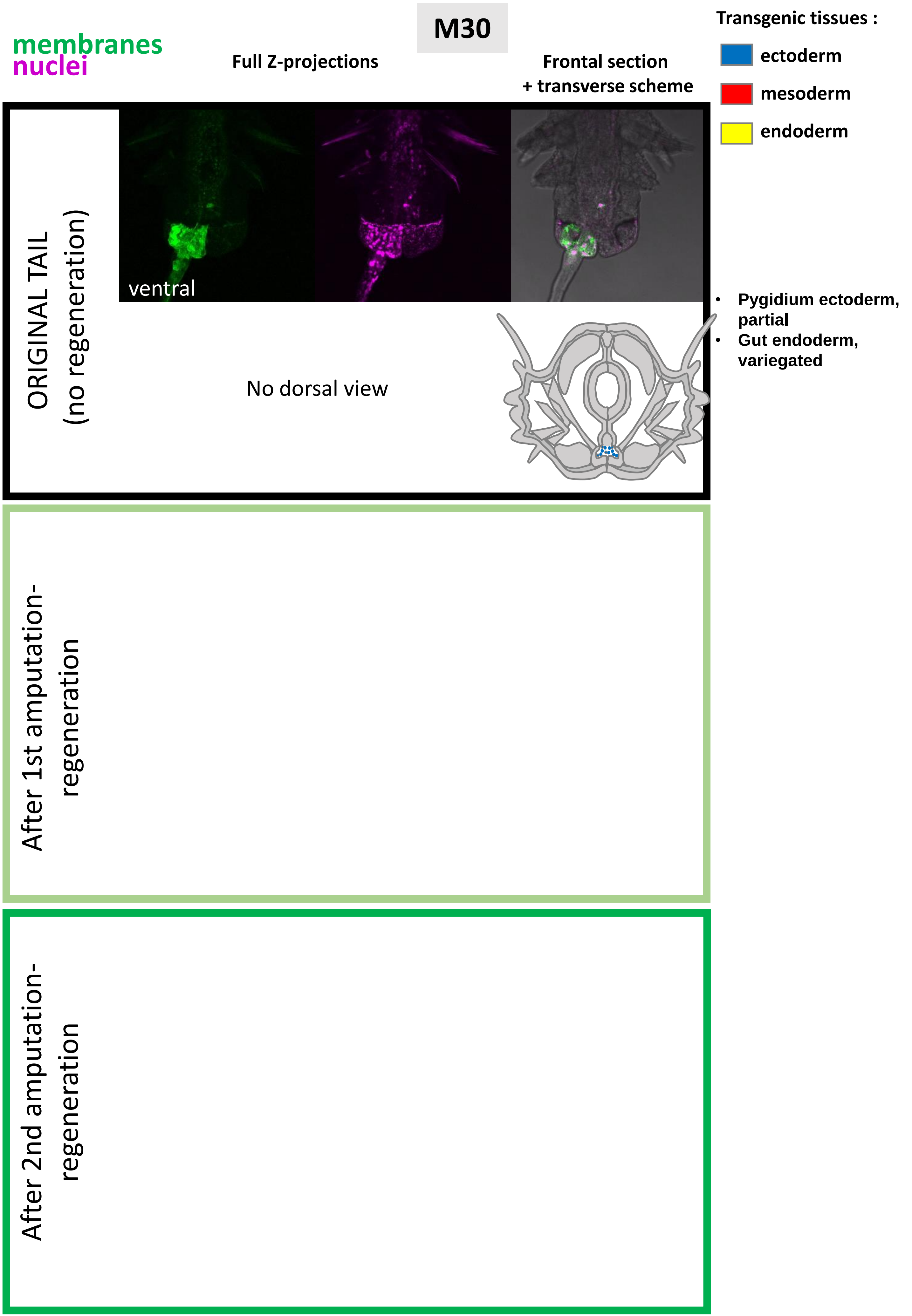
M30
membranes
nuclei
Full Z-projections
Frontal section
+ transverse scheme
Transgenic tissues :
ectoderm
mesoderm
endoderm
ORIGINAL TAIL
(no regeneration)
ventral
No dorsal view
• Pygidium ectoderm, partial
• Gut endoderm, variegated
After 1st amputation-regeneration
After 2nd amputation-regeneration

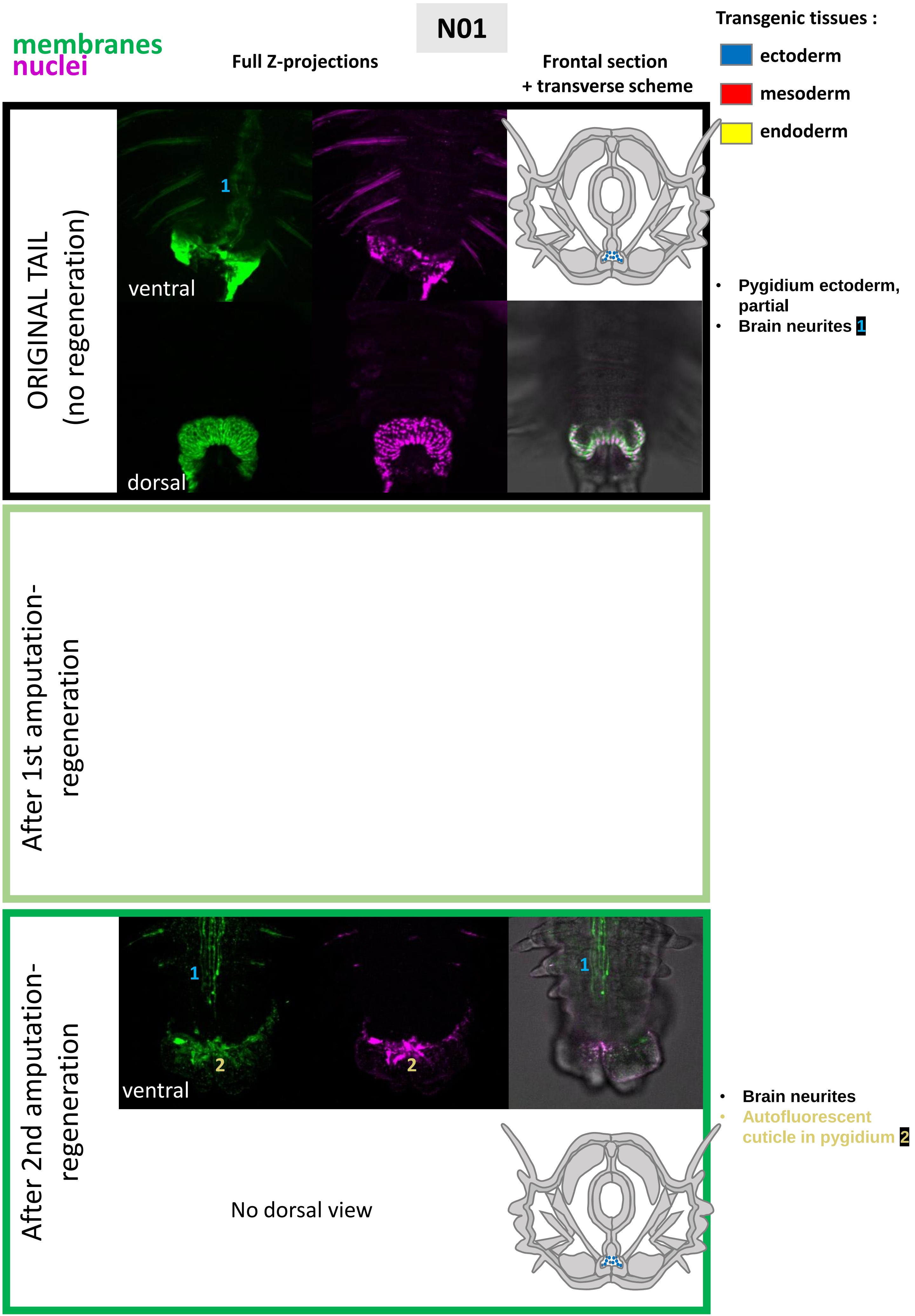
N01
membranes
nuclei
Full Z-projections
Frontal section
+ transverse scheme
Transgenic tissues :
ectoderm
mesoderm
endoderm
ORIGINAL TAIL
(no regeneration)
1
ventral
dorsal
• Pygidium ectoderm, partial
• Brain neurites 1
After 1st amputation-regeneration
After 2nd amputation-regeneration
1
2
2
1
ventral
No dorsal view
• Brain neurites
• Autofluorescent cuticle in pygidium 2

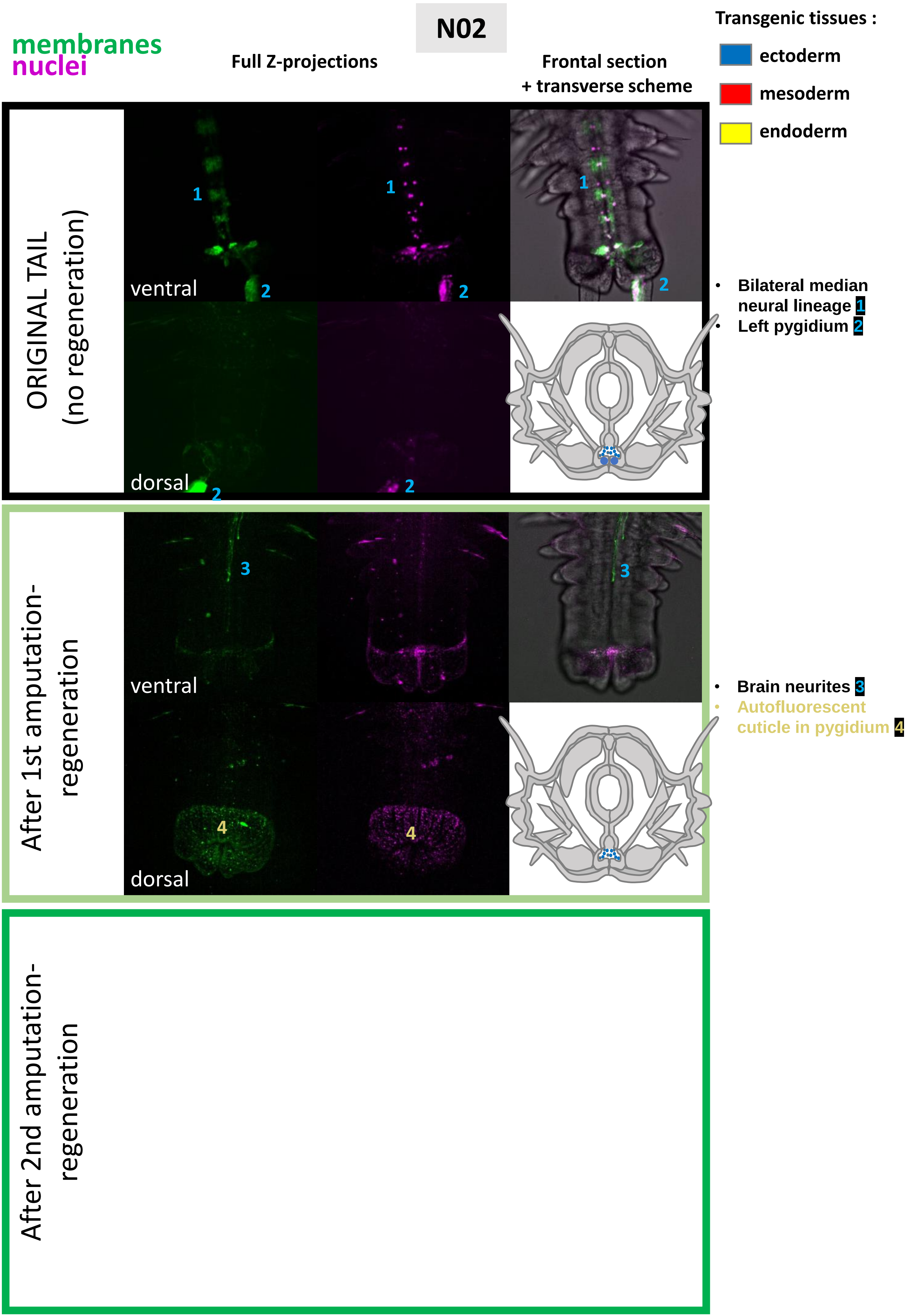
N02
membranes
nuclei
Full Z-projections
Frontal section
+ transverse scheme
Transgenic tissues :
ectoderm
mesoderm
endoderm
ORIGINAL TAIL
(no regeneration)
ventral
dorsal
1
2
Bilateral median neural lineage 1
Left pygidium 2
After 1st amputation-regeneration
3
4
Brain neurites 3
Autofluorescent cuticle in pygidium 4
After 2nd amputation-regeneration

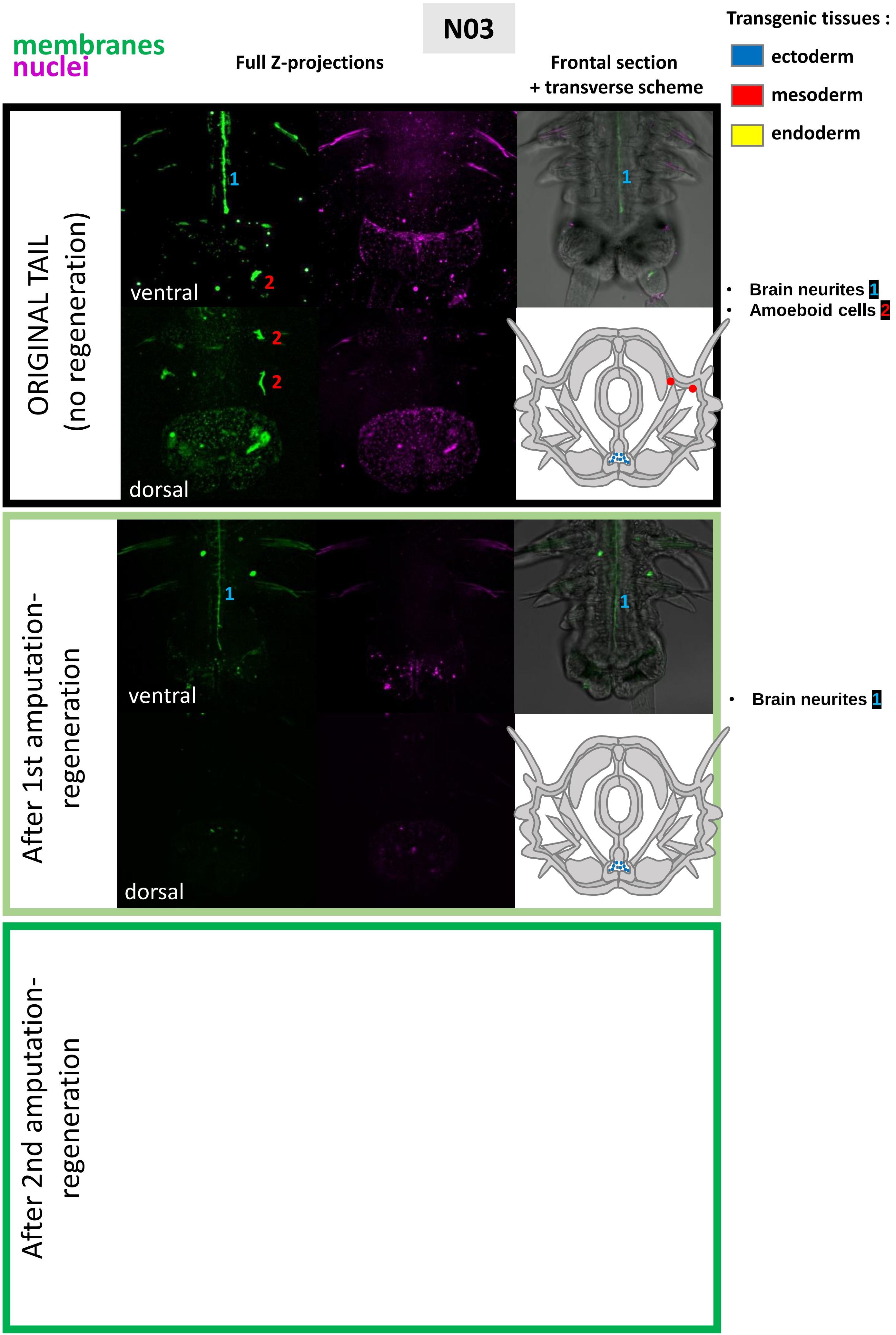
N03
membranes
nuclei
Full Z-projections
Frontal section
+ transverse scheme
Transgenic tissues :
ectoderm
mesoderm
endoderm
ORIGINAL TAIL
(no regeneration)
ventral
dorsal
1
2
Brain neurites 1
Amoeboid cells 2
After 1st amputation-
regeneration
Brain neurites 1
After 2nd amputation-
regeneration

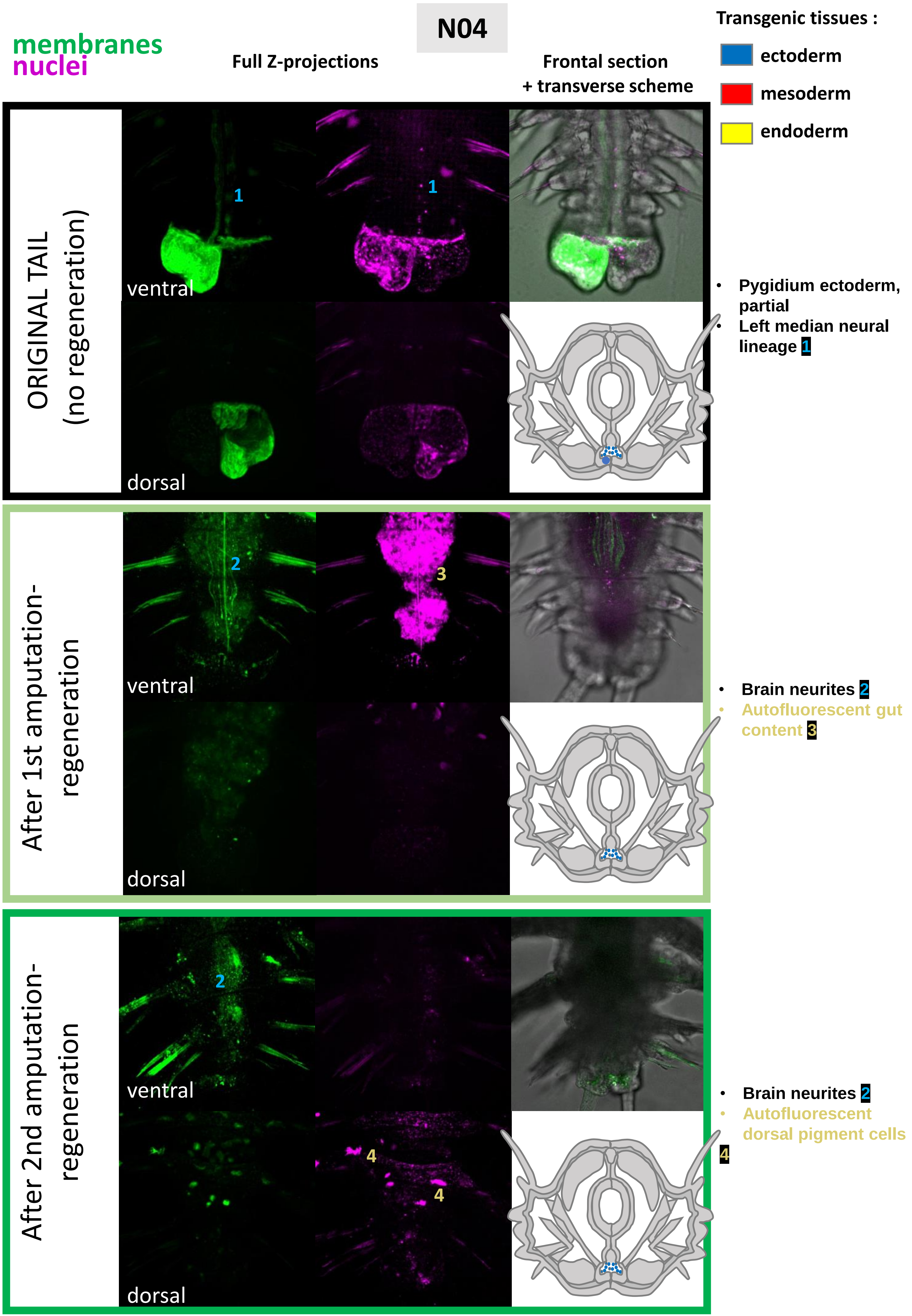
N04
membranes
nuclei
Full Z-projections
Frontal section
+ transverse scheme
Transgenic tissues :
ectoderm
mesoderm
endoderm
ORIGINAL TAIL
(no regeneration)
ventral
dorsal
1
1
Pygidium ectoderm, partial
Left median neural lineage 1
After 1st amputation-regeneration
2
3
Brain neurites 2
Autofluorescent gut content 3
After 2nd amputation-regeneration
2
4
4
Brain neurites 2
Autofluorescent dorsal pigment cells 4

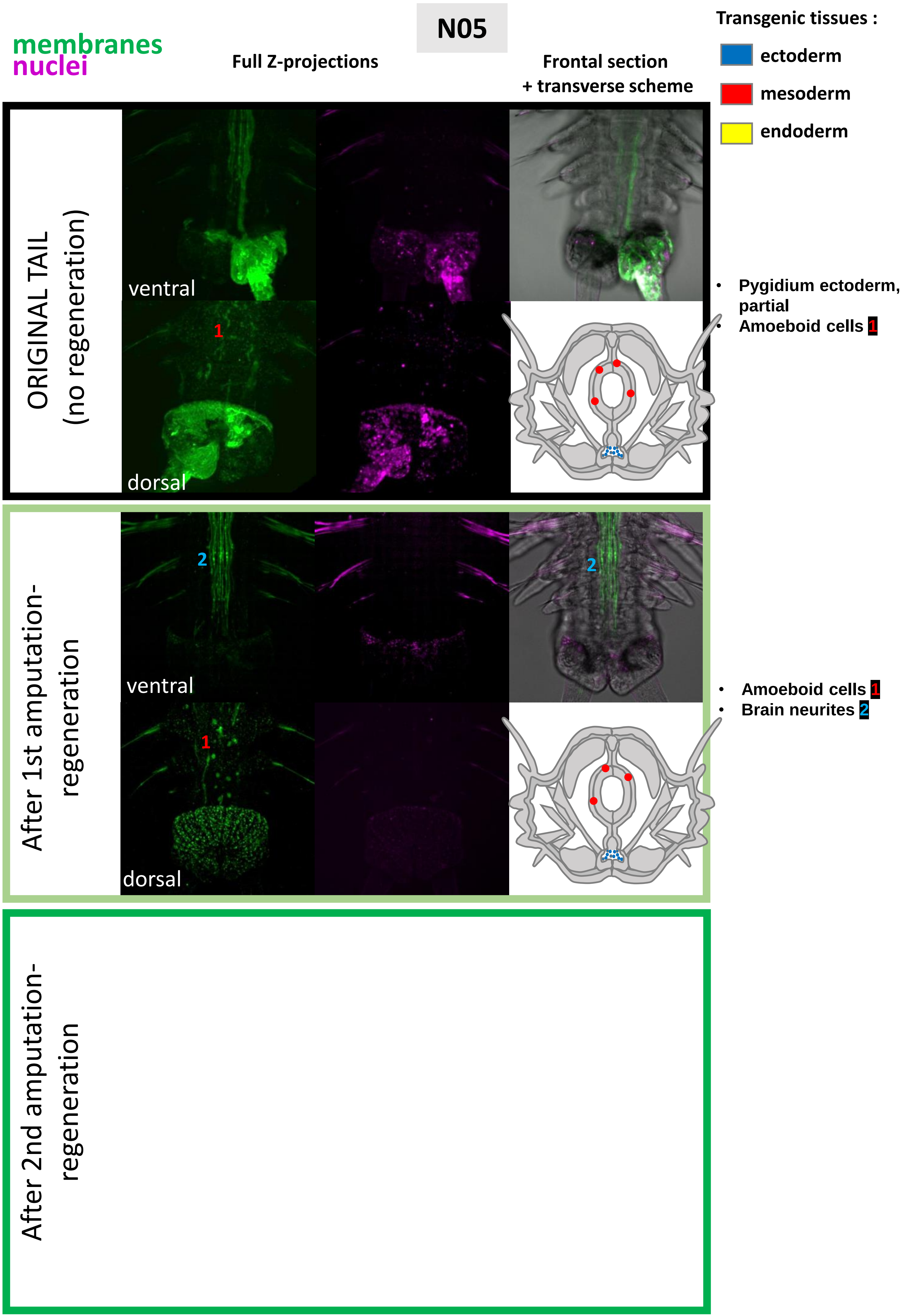
N05
membranes
nuclei
Full Z-projections
Frontal section
+ transverse scheme
Transgenic tissues :
ectoderm
mesoderm
endoderm
ORIGINAL TAIL
(no regeneration)
ventral
dorsal
1
• Pygidium ectoderm, partial
• Amoeboid cells 1
After 1st amputation-regeneration
2
1
• Amoeboid cells 1
• Brain neurites 2
After 2nd amputation-regeneration

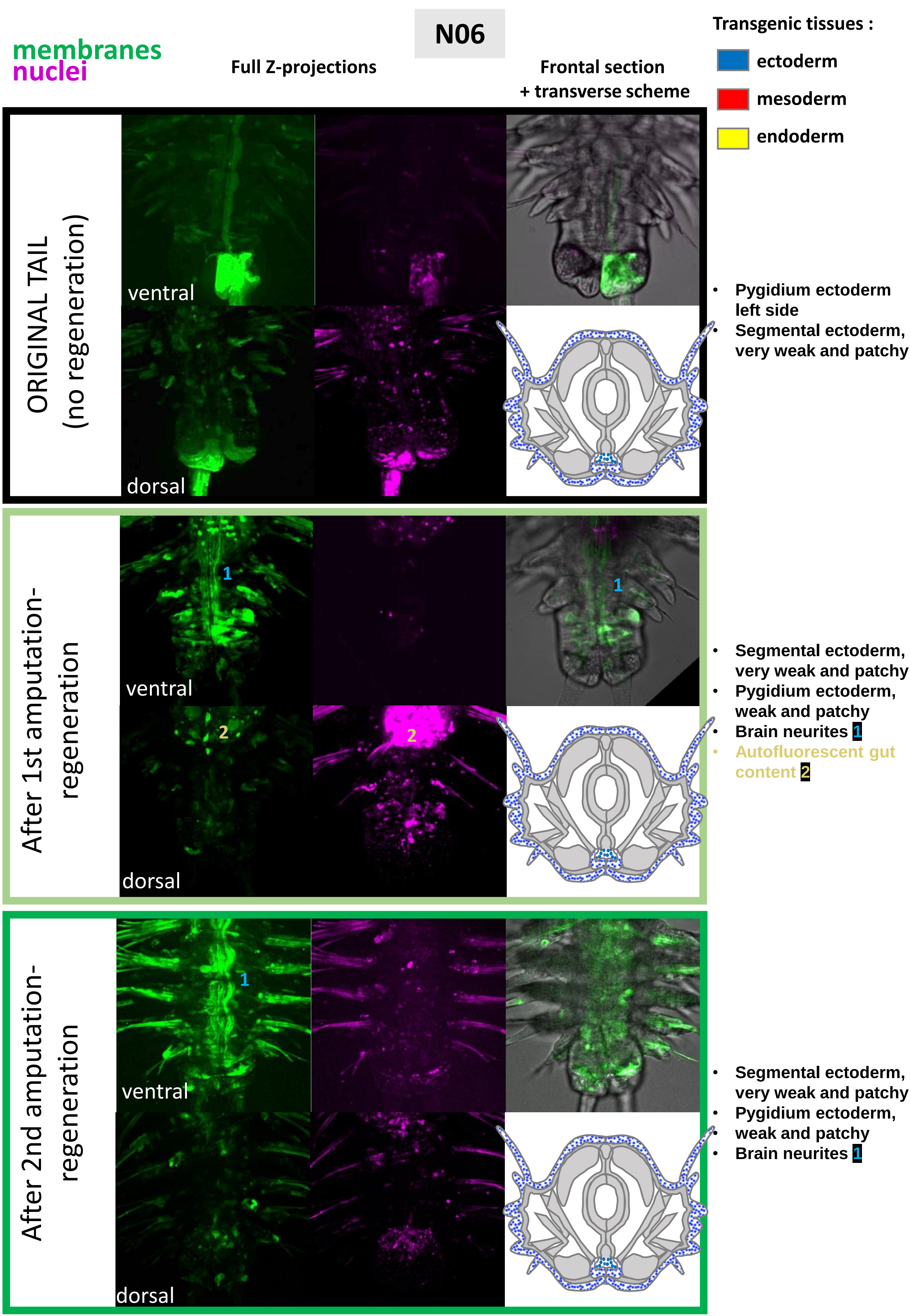
N06
membranes
nuclei
Full Z-projections
Frontal section
+ transverse scheme
Transgenic tissues :
ectoderm
mesoderm
endoderm
ORIGINAL TAIL
(no regeneration)
ventral
dorsal
• Pygidium ectoderm left side
• Segmental ectoderm, very weak and patchy
After 1st amputation-regeneration
ventral
dorsal
1
2
• Segmental ectoderm, very weak and patchy
• Pygidium ectoderm, weak and patchy
• Brain neurites 1
• Autofluorescent gut content 2
After 2nd amputation-regeneration
ventral
dorsal
1
• Segmental ectoderm, very weak and patchy
• Pygidium ectoderm, weak and patchy
• Brain neurites 1

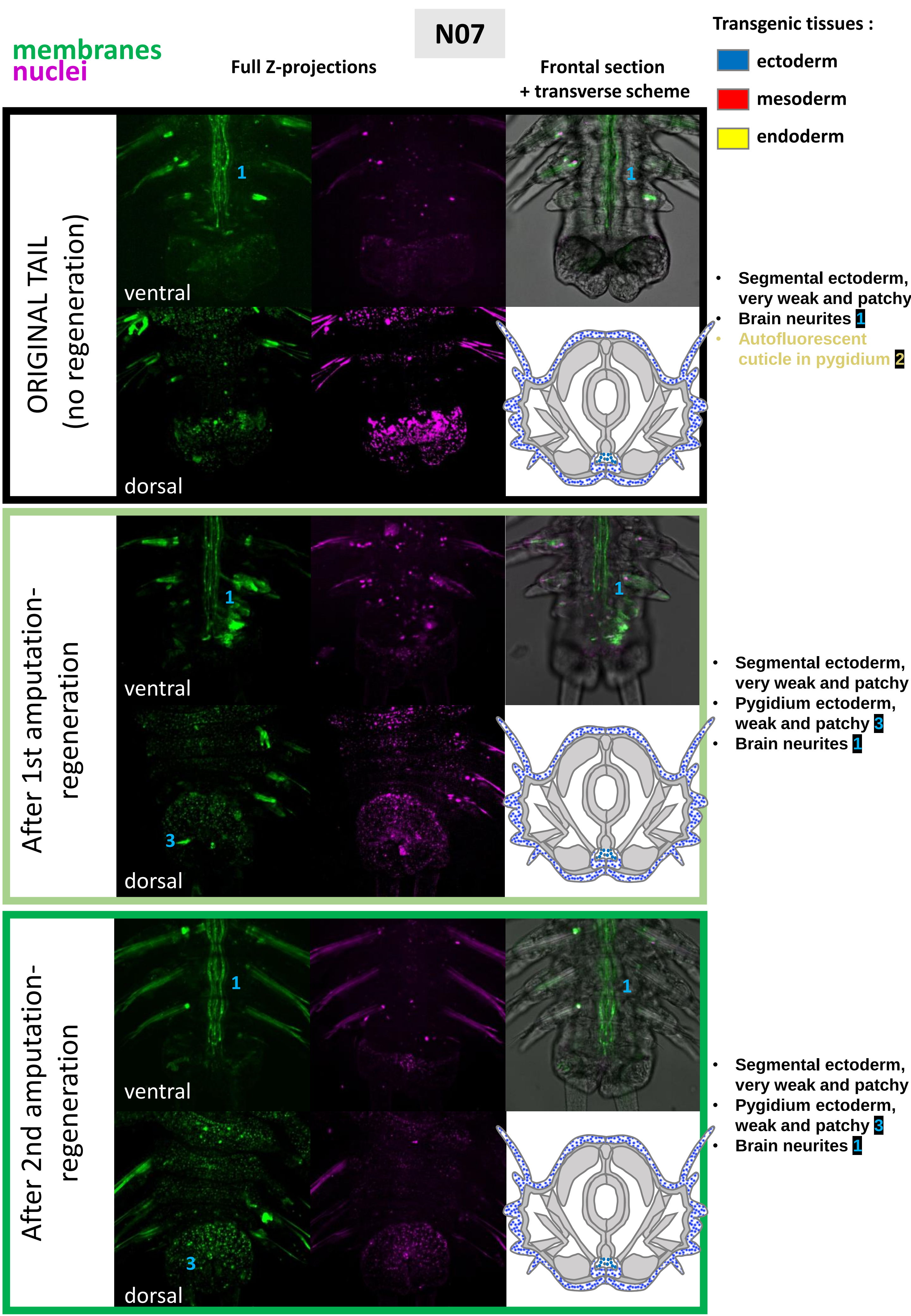

N07
membranes
nuclei
Full Z-projections
Frontal section
+ transverse scheme
Transgenic tissues :
ectoderm
mesoderm
endoderm
ORIGINAL TAIL
(no regeneration)
ventral
dorsal
1
• Segmental ectoderm, very weak and patchy
• Brain neurites 1
• Autofluorescent cuticle in pygidium 2
After 1st amputation-regeneration
ventral
dorsal
1
3
• Segmental ectoderm, very weak and patchy
• Pygidium ectoderm, weak and patchy 3
• Brain neurites 1
After 2nd amputation-regeneration
ventral
dorsal
1
3
• Segmental ectoderm, very weak and patchy
• Pygidium ectoderm, weak and patchy 3
• Brain neurites 1

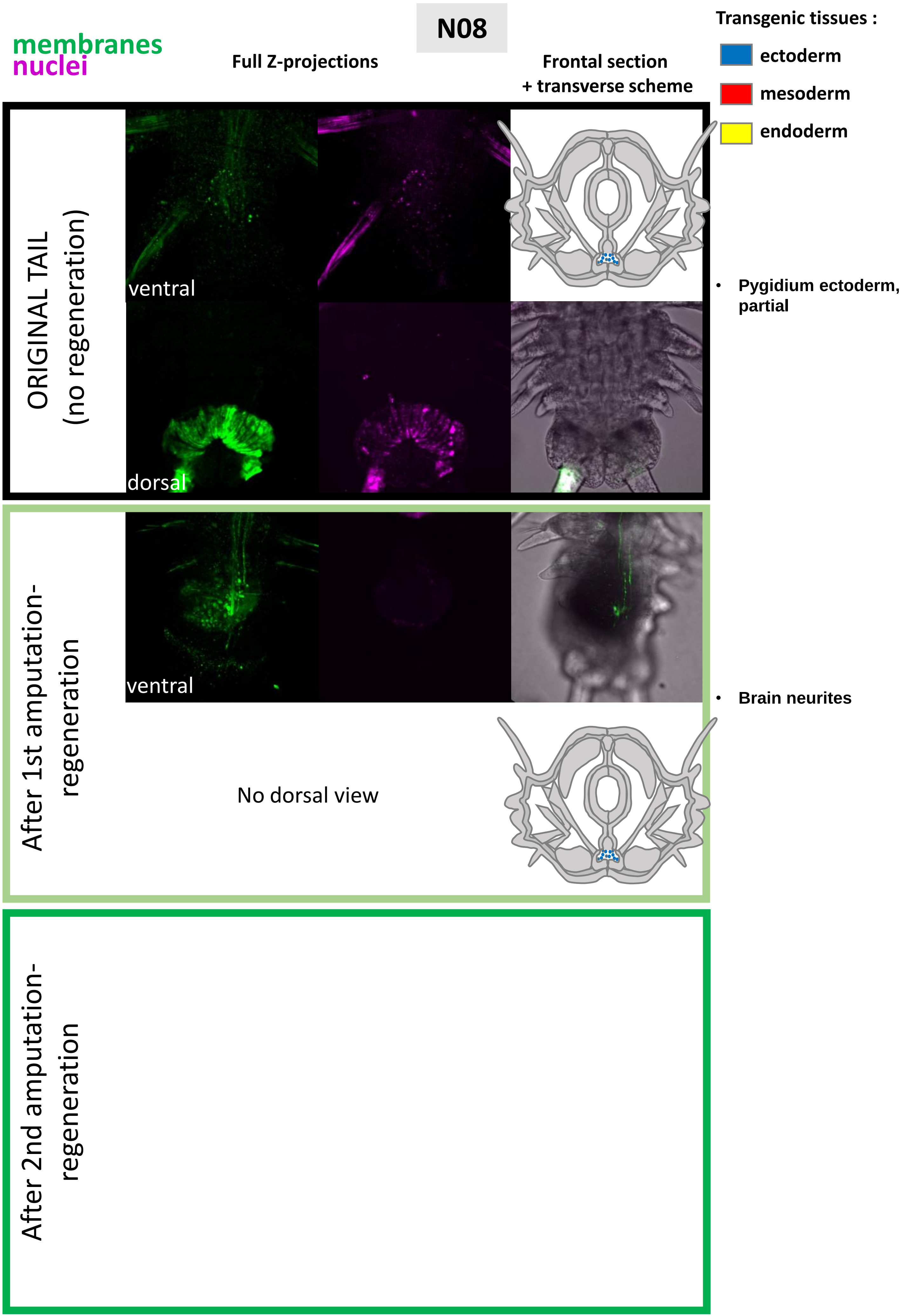
N08
membranes
nuclei
Full Z-projections
Frontal section
+ transverse scheme
Transgenic tissues :
ectoderm
mesoderm
endoderm
ORIGINAL TAIL
(no regeneration)
ventral
dorsal
• Pygidium ectoderm, partial
After 1st amputation-
regeneration
ventral
No dorsal view
• Brain neurites
After 2nd amputation-
regeneration

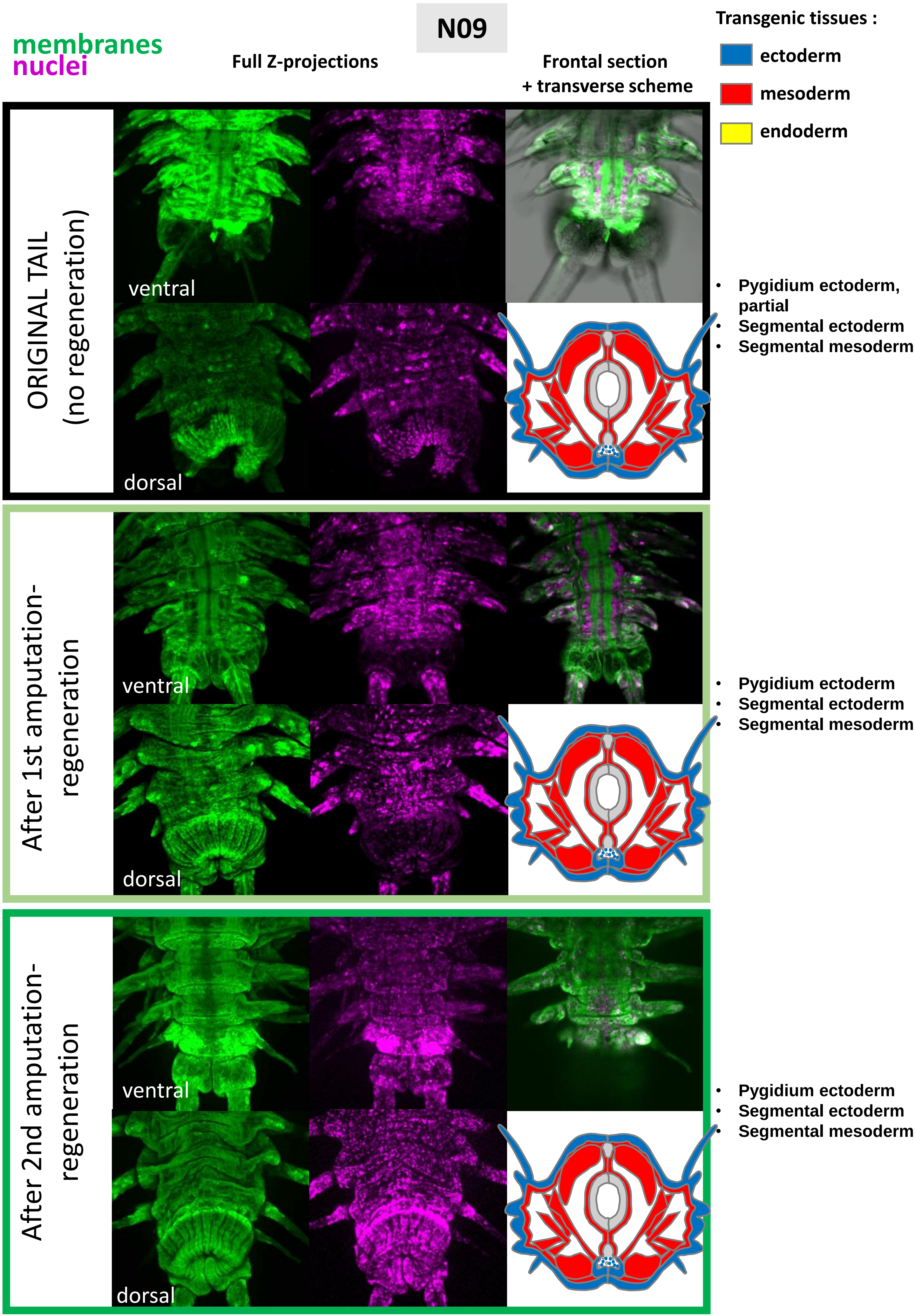

N09
membranes
nuclei
Full Z-projections
Frontal section
+ transverse scheme
Transgenic tissues :
ectoderm
mesoderm
endoderm
ORIGINAL TAIL
(no regeneration)
ventral
dorsal
Pygidium ectoderm, partial
Segmental ectoderm
Segmental mesoderm
After 1st amputation-regeneration
ventral
dorsal
Pygidium ectoderm
Segmental ectoderm
Segmental mesoderm
After 2nd amputation-regeneration
ventral
dorsal
Pygidium ectoderm
Segmental ectoderm
Segmental mesoderm

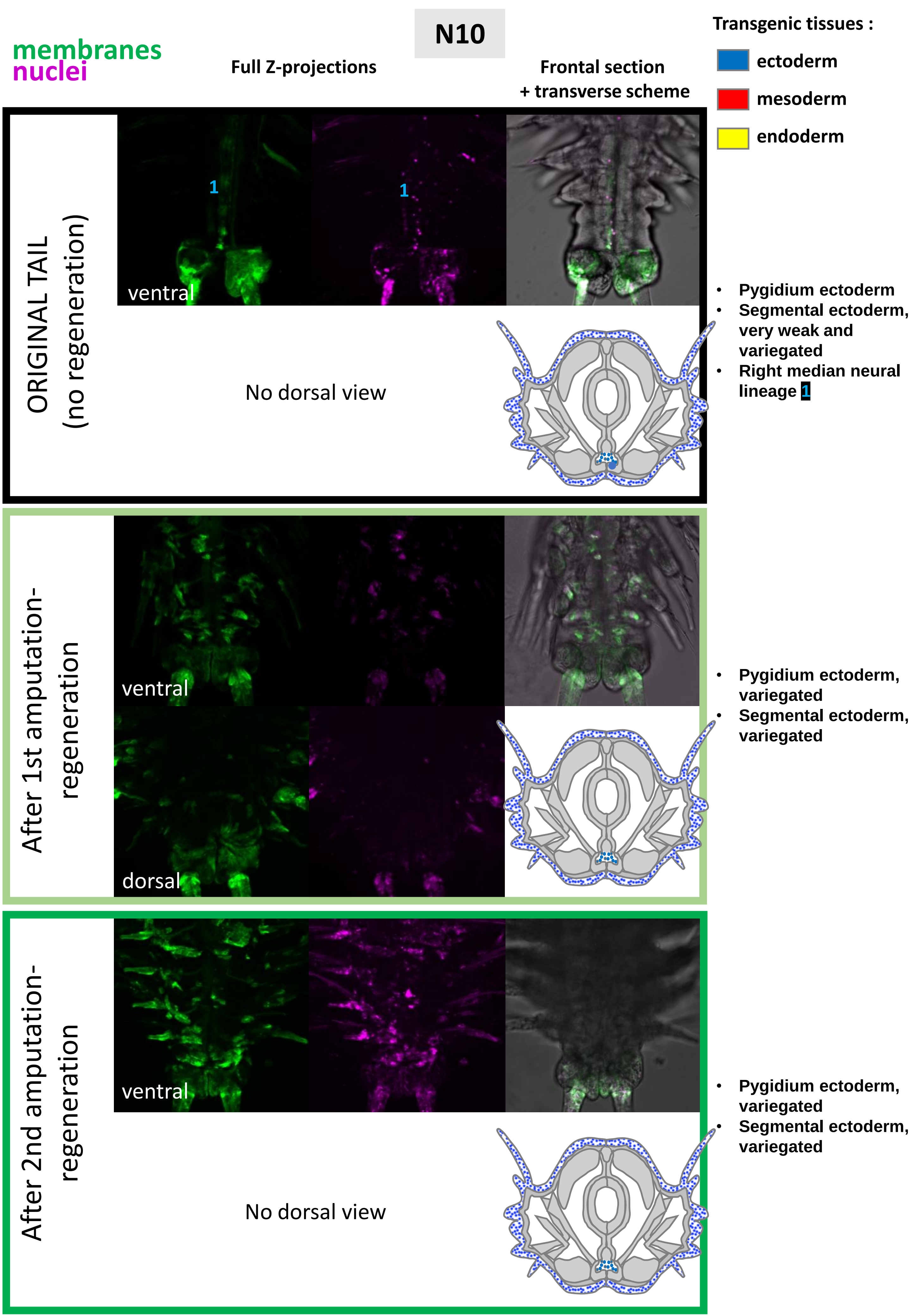
N10
membranes
nuclei
Full Z-projections
Frontal section
+ transverse scheme
Transgenic tissues :
ectoderm
mesoderm
endoderm
ORIGINAL TAIL
(no regeneration)
1
1
ventral
No dorsal view
• Pygidium ectoderm
• Segmental ectoderm, very weak and variegated
• Right median neural lineage 1
After 1st amputation-regeneration
ventral
dorsal
• Pygidium ectoderm, variegated
• Segmental ectoderm, variegated
After 2nd amputation-regeneration
ventral
No dorsal view
• Pygidium ectoderm, variegated
• Segmental ectoderm, variegated

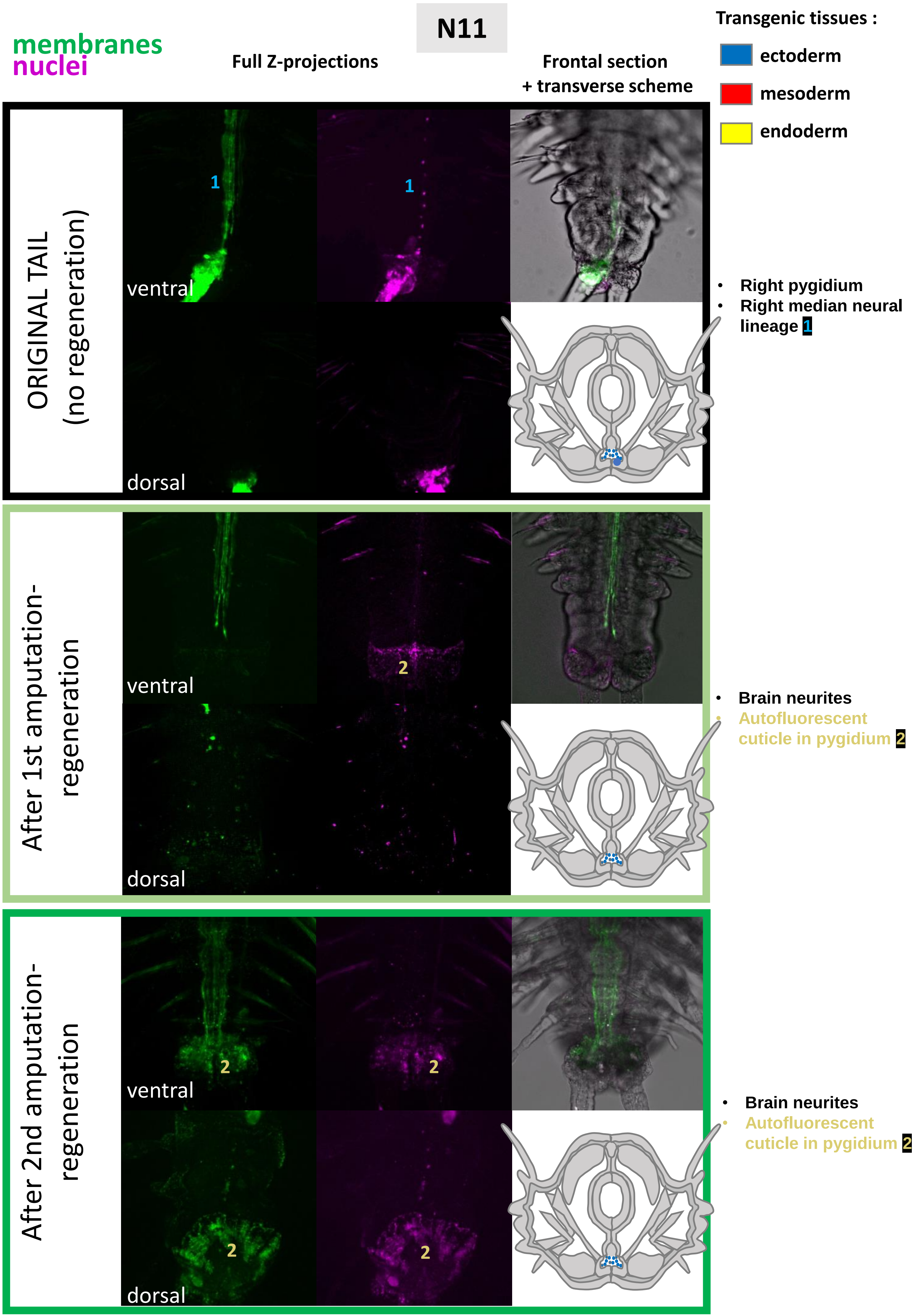
N11
membranes
nuclei
Full Z-projections
Frontal section
+ transverse scheme
Transgenic tissues :
ectoderm
mesoderm
endoderm
ORIGINAL TAIL
(no regeneration)
1
1
ventral
dorsal
Right pygidium
Right median neural lineage 1
After 1st amputation-regeneration
2
ventral
dorsal
Brain neurites
Autofluorescent cuticle in pygidium 2
After 2nd amputation-regeneration
2
2
ventral
2
2
dorsal
Brain neurites
Autofluorescent cuticle in pygidium 2

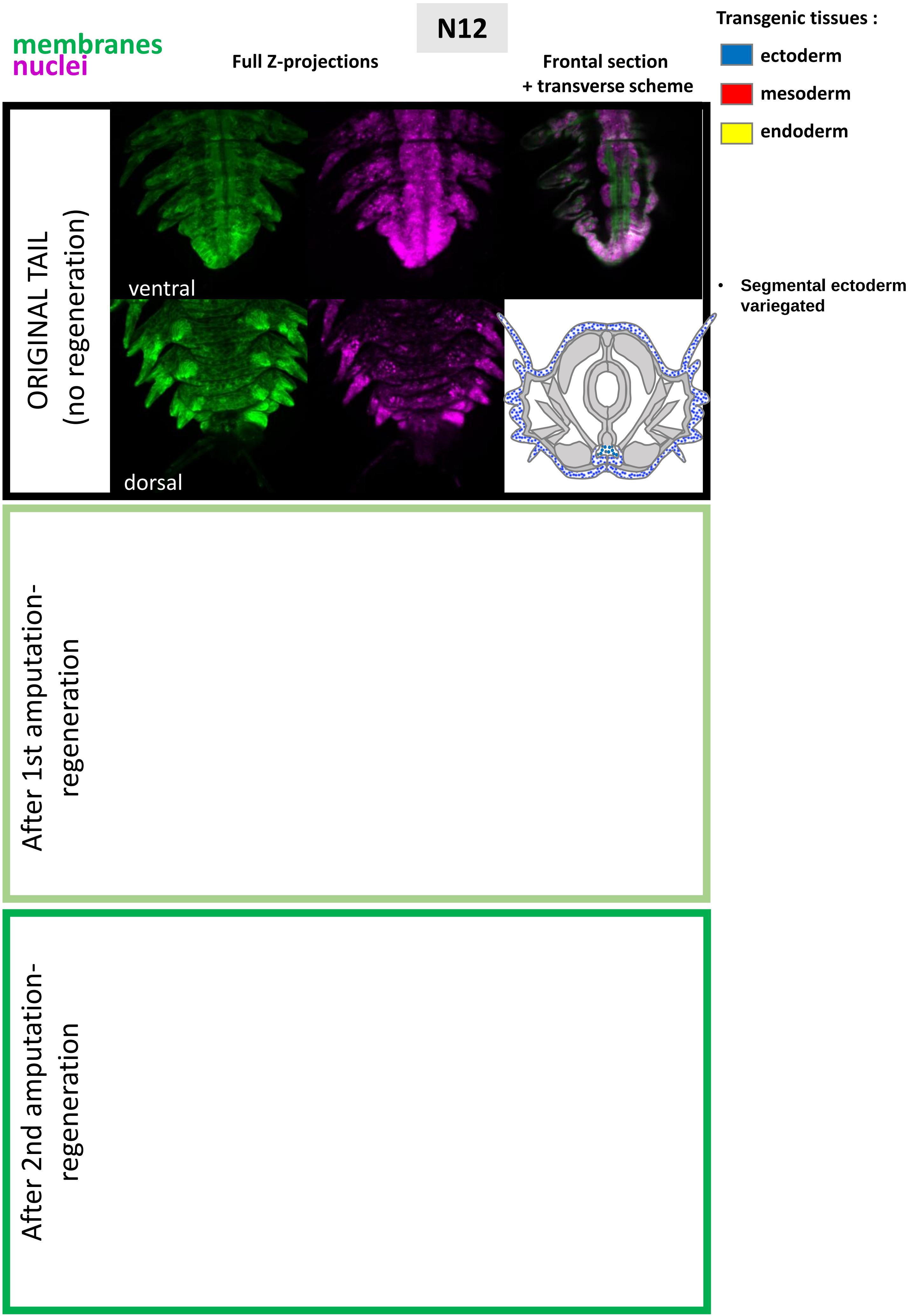

N12
membranes
nuclei
Full Z-projections
Frontal section
+ transverse scheme
Transgenic tissues :
ectoderm
mesoderm
endoderm
ORIGINAL TAIL
(no regeneration)
ventral
dorsal
Segmental ectoderm variegated
After 1st amputation-
regeneration
After 2nd amputation-
regeneration

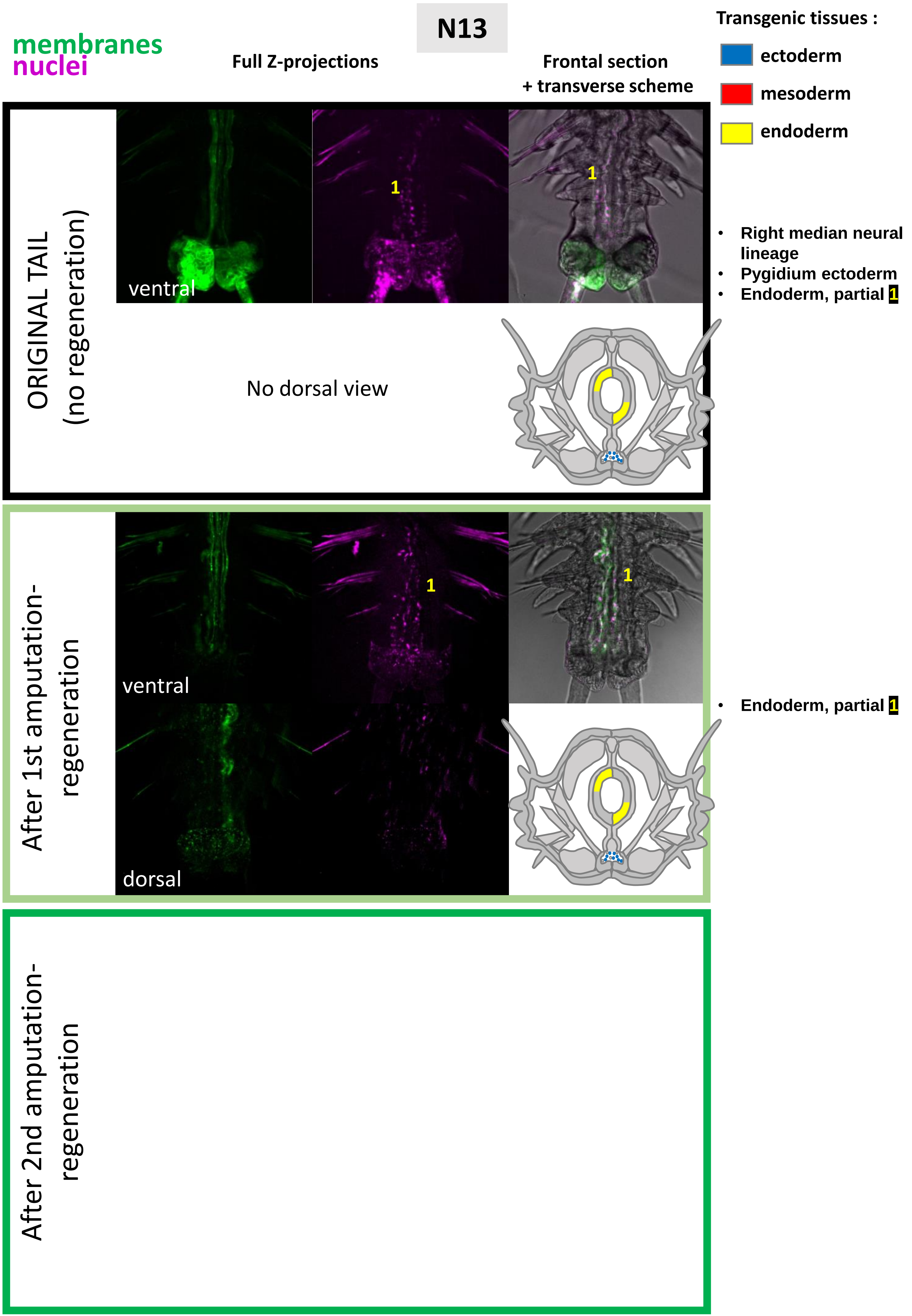
N13
membranes
nuclei
Full Z-projections
Frontal section
+ transverse scheme
Transgenic tissues :
ectoderm
mesoderm
endoderm
ORIGINAL TAIL
(no regeneration)
ventral
1
1
No dorsal view
Right median neural lineage
Pygidium ectoderm
Endoderm, partial 1
After 1st amputation-regeneration
ventral
dorsal
1
1
Endoderm, partial 1
After 2nd amputation-regeneration

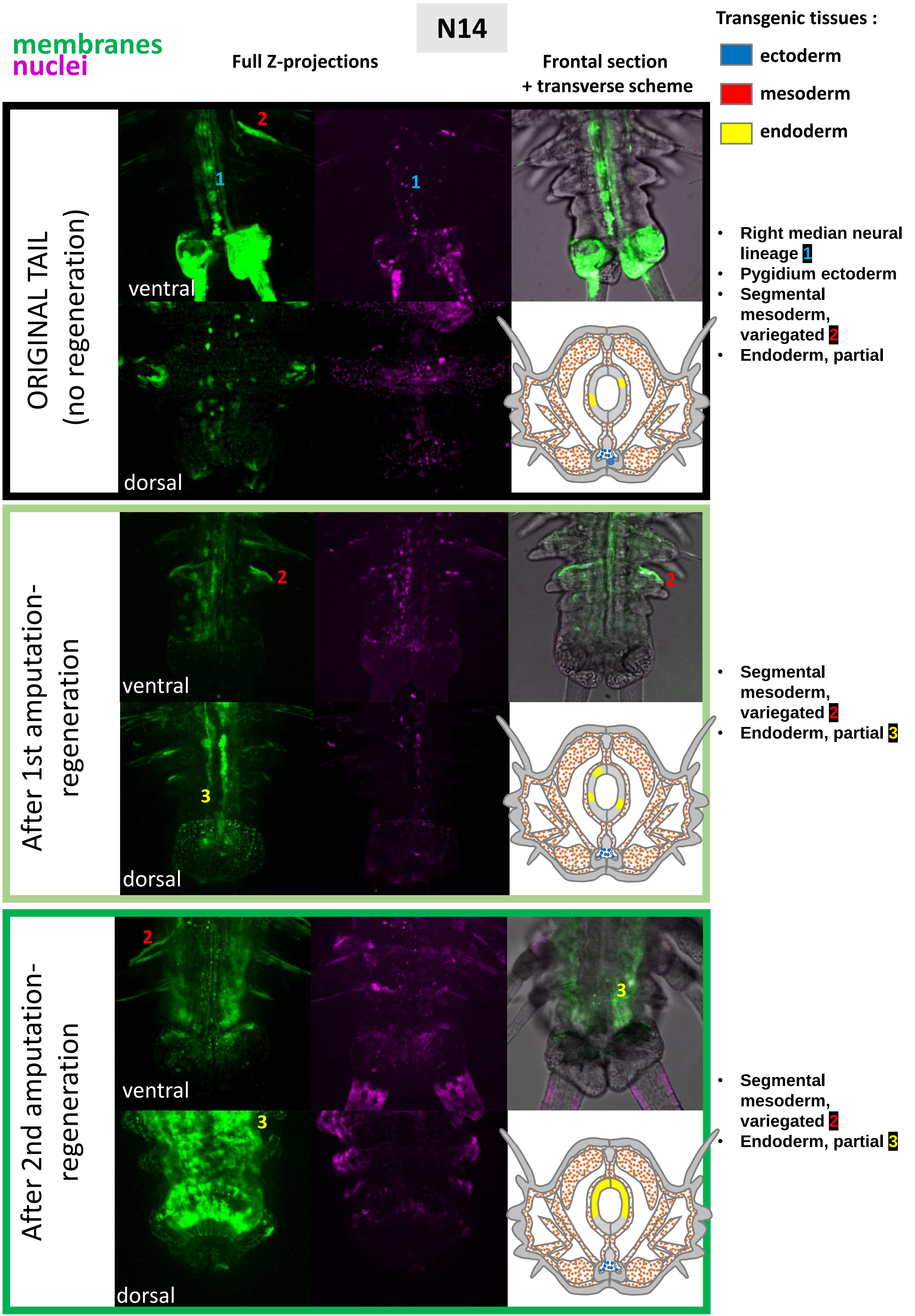
N14
membranes
nuclei
Full Z-projections
Frontal section
+ transverse scheme
Transgenic tissues :
ectoderm
mesoderm
endoderm
ORIGINAL TAIL
(no regeneration)
ventral
dorsal
1
2
Right median neural lineage 1
Pygidium ectoderm
Segmental mesoderm, variegated 2
Endoderm, partial
After 1st amputation-regeneration
ventral
dorsal
2
3
Segmental mesoderm, variegated 2
Endoderm, partial 3
After 2nd amputation-regeneration
ventral
dorsal
2
3
Segmental mesoderm, variegated 2
Endoderm, partial 3

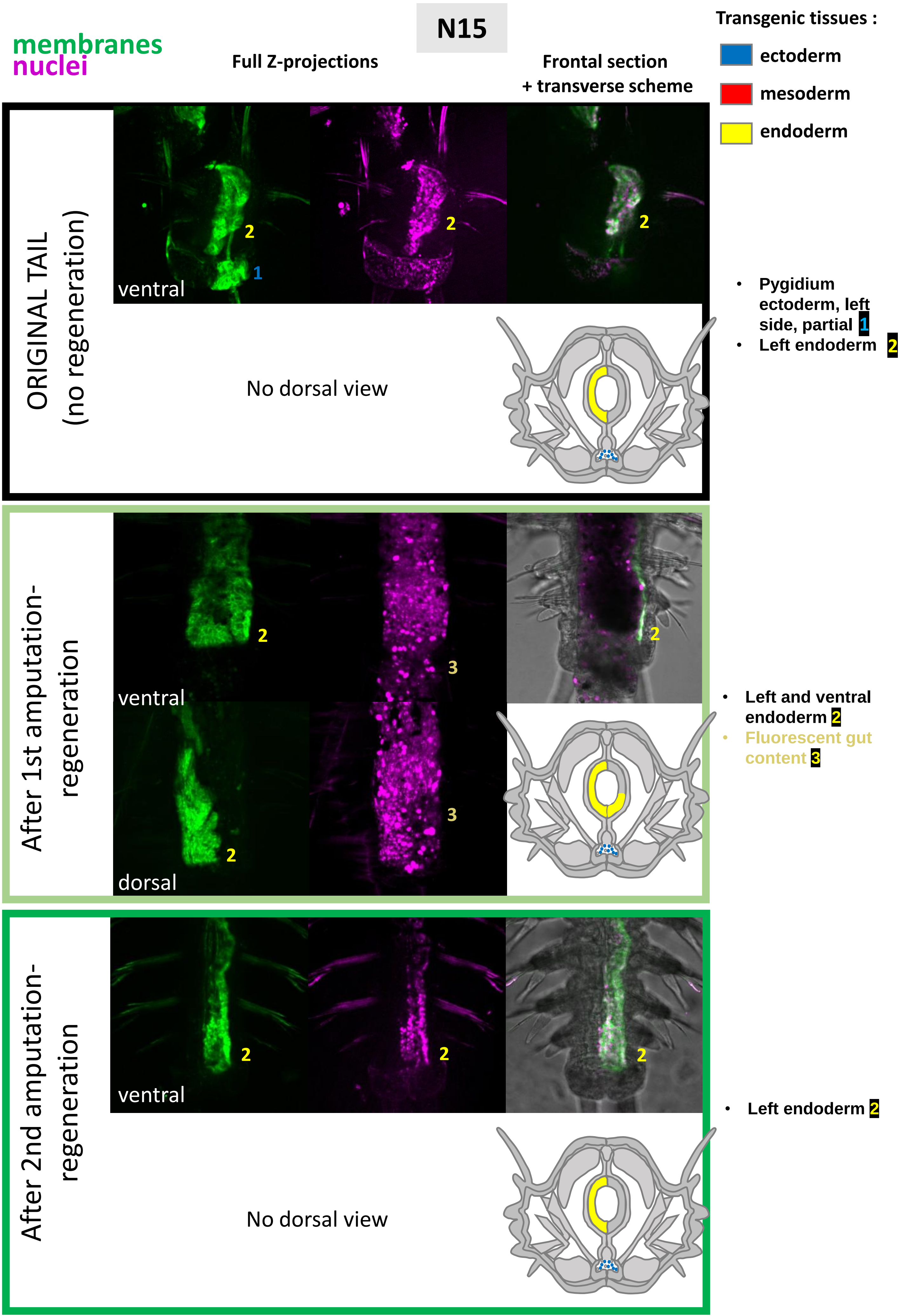
N15
membranes
nuclei
Full Z-projections
Frontal section
+ transverse scheme
Transgenic tissues :
ectoderm
mesoderm
endoderm
ORIGINAL TAIL
(no regeneration)
ventral
No dorsal view
Pygidium ectoderm, left side, partial 1
Left endoderm 2
After 1st amputation-regeneration
ventral
dorsal
Left and ventral endoderm 2
Fluorescent gut content 3
After 2nd amputation-regeneration
ventral
No dorsal view
Left endoderm 2

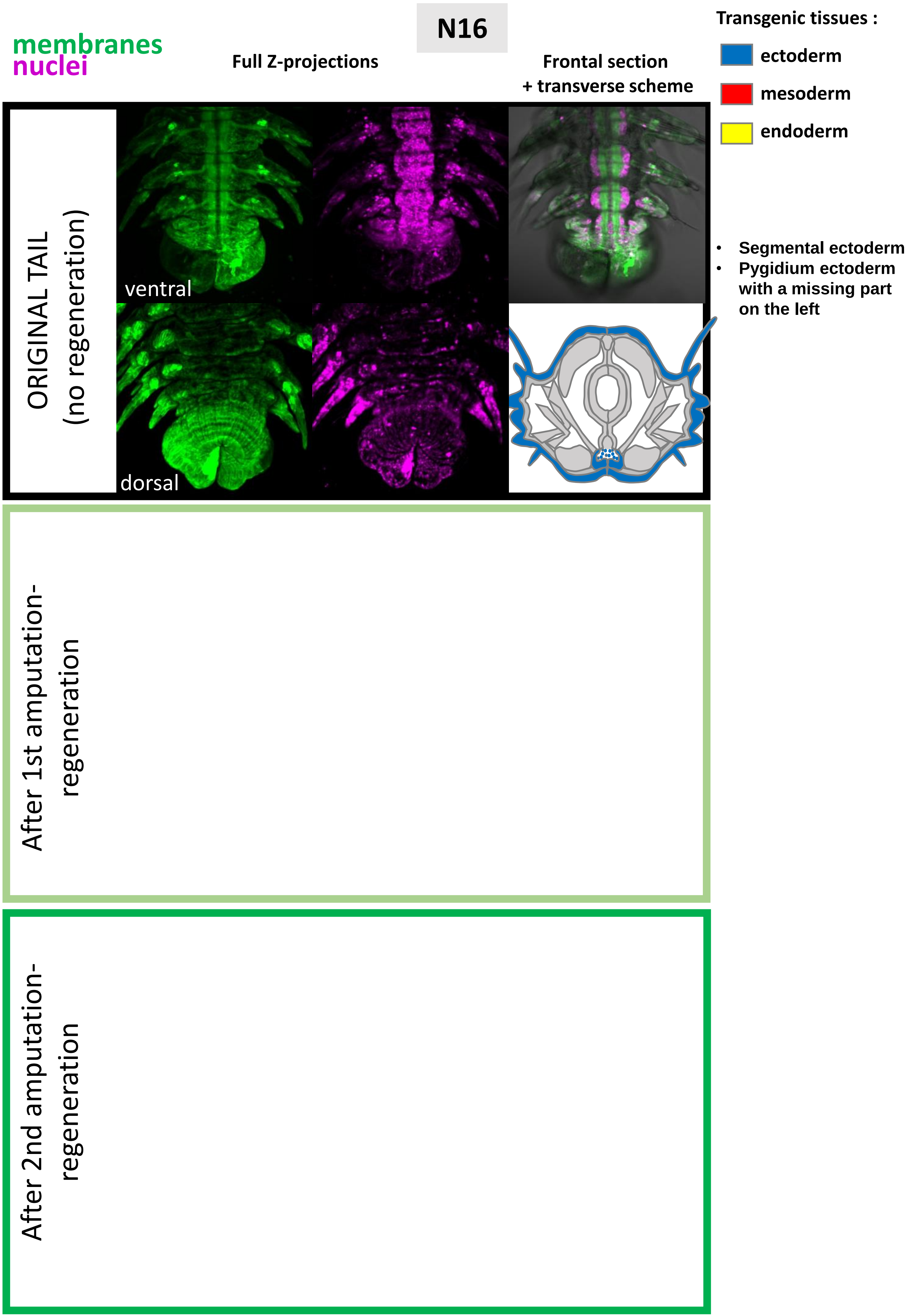

N16
membranes
nuclei
Full Z-projections
Frontal section
+ transverse scheme
Transgenic tissues :
ectoderm
mesoderm
endoderm
ORIGINAL TAIL
(no regeneration)
ventral
dorsal
Segmental ectoderm
Pygidium ectoderm with a missing part on the left
After 1st amputation-regeneration
After 2nd amputation-regeneration

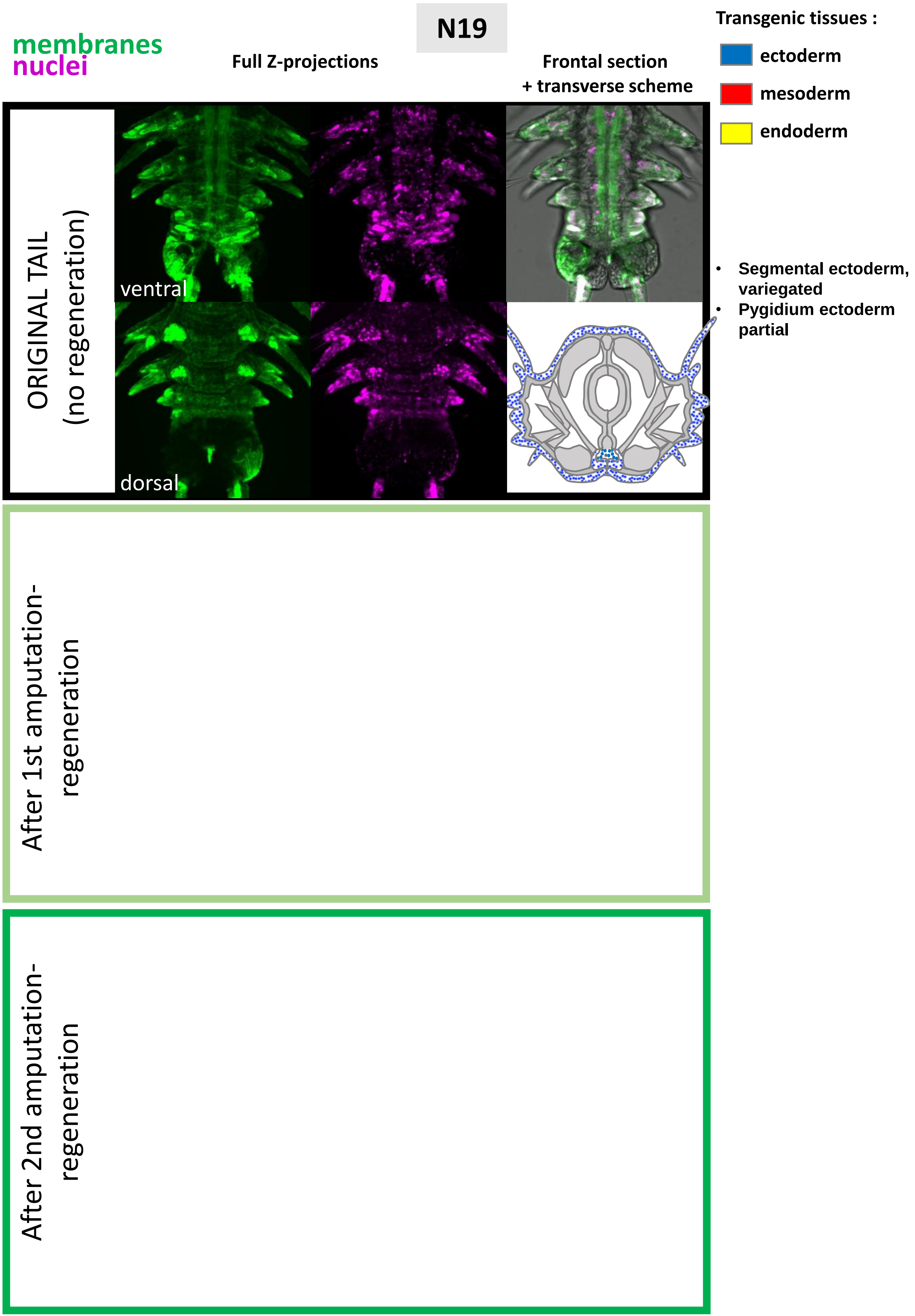

- Segmental ectoderm, variegated
- Pygidium ectoderm partial

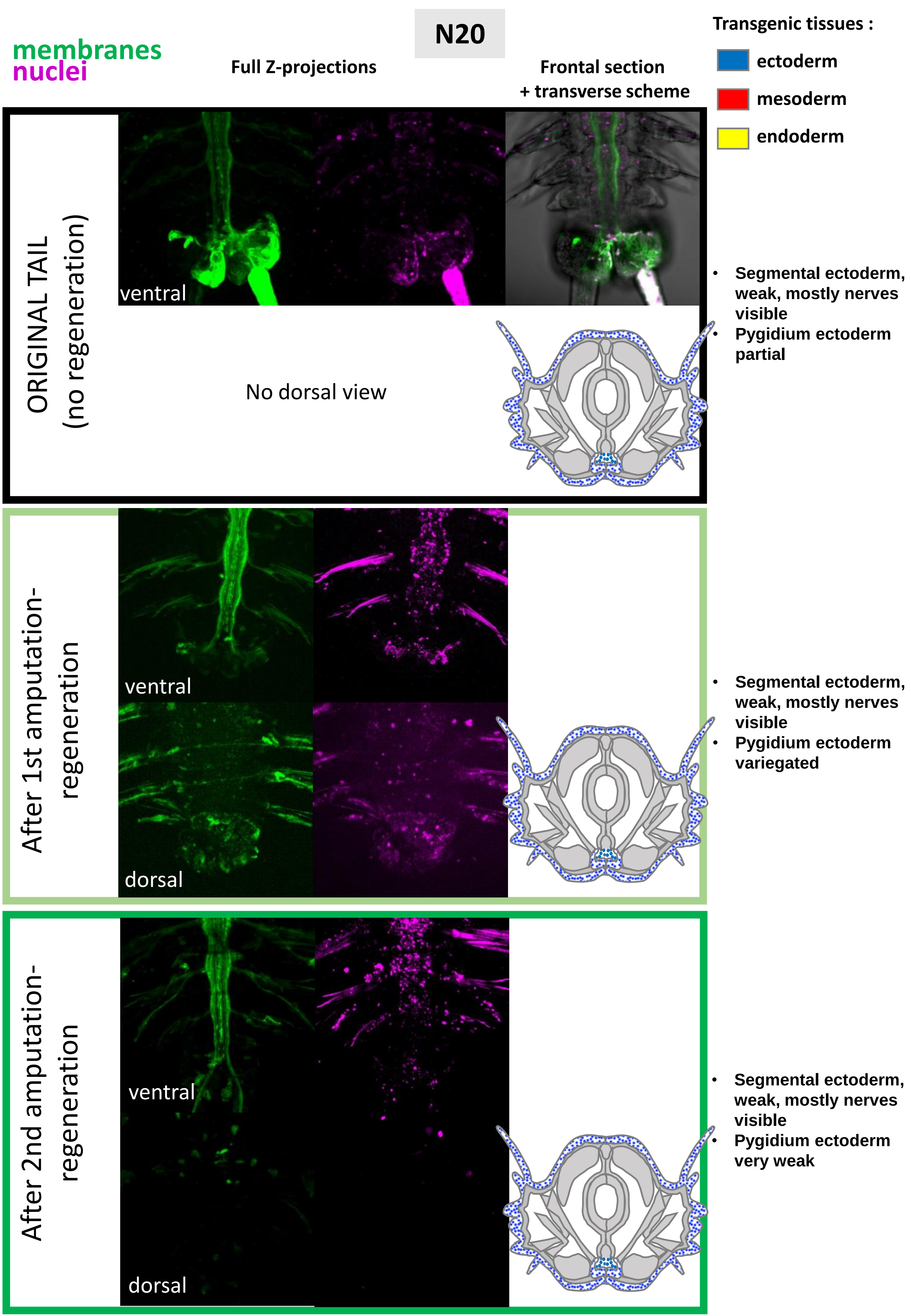
N20
membranes
nuclei
Full Z-projections
Frontal section
+ transverse scheme
Transgenic tissues :
ectoderm
mesoderm
endoderm
ORIGINAL TAIL
(no regeneration)
ventral
No dorsal view
Segmental ectoderm, weak, mostly nerves visible
Pygidium ectoderm partial
After 1st amputation-regeneration
ventral
dorsal
Segmental ectoderm, weak, mostly nerves visible
Pygidium ectoderm variegated
After 2nd amputation-regeneration
ventral
dorsal
Segmental ectoderm, weak, mostly nerves visible
Pygidium ectoderm very weak

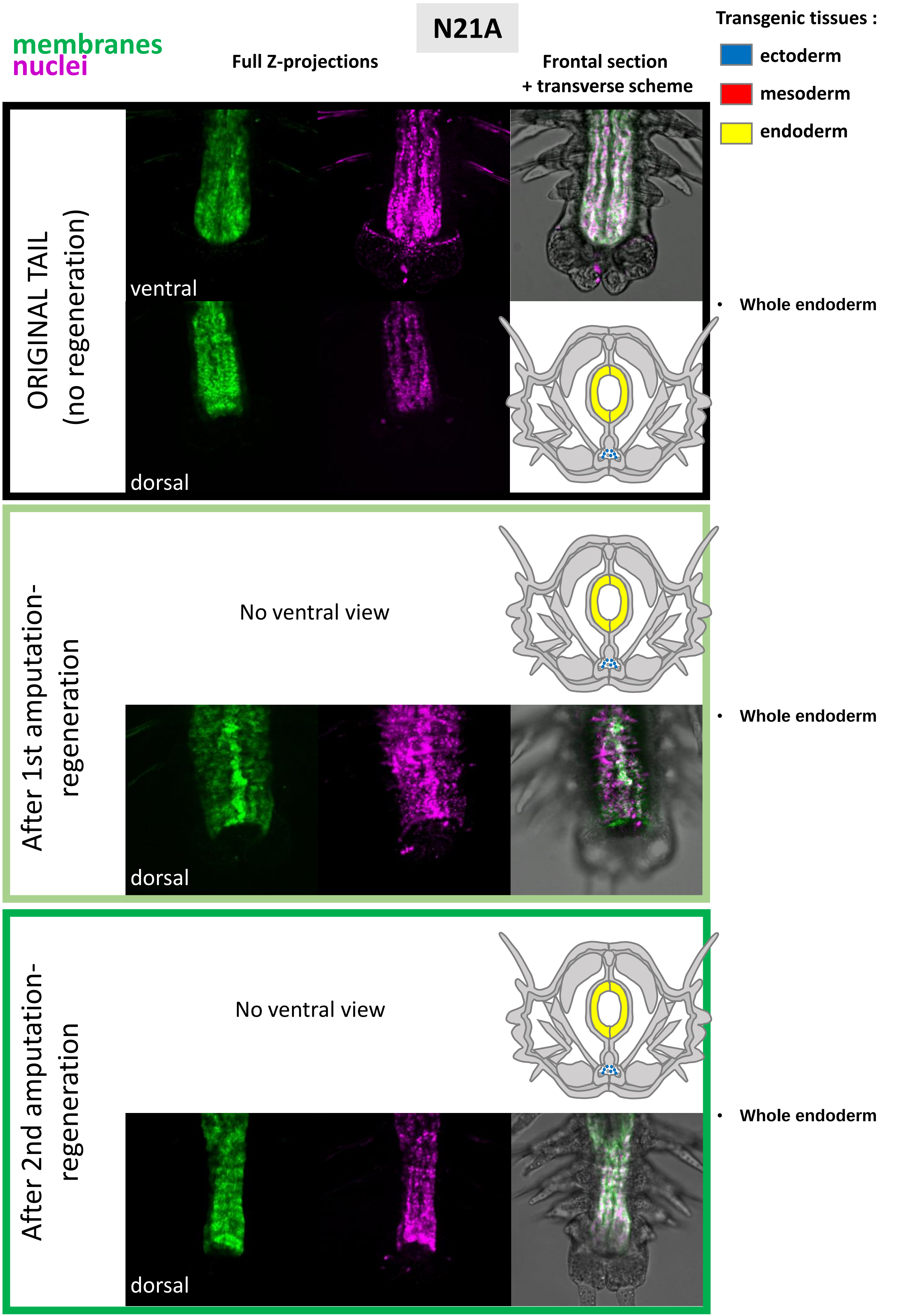

N21A
membranes
nuclei
Full Z-projections
Frontal section
+ transverse scheme
Transgenic tissues :
ectoderm
mesoderm
endoderm
ORIGINAL TAIL
(no regeneration)
ventral
dorsal
Whole endoderm
After 1st amputation-
regeneration
No ventral view
dorsal
Whole endoderm
After 2nd amputation-
regeneration
No ventral view
dorsal
Whole endoderm

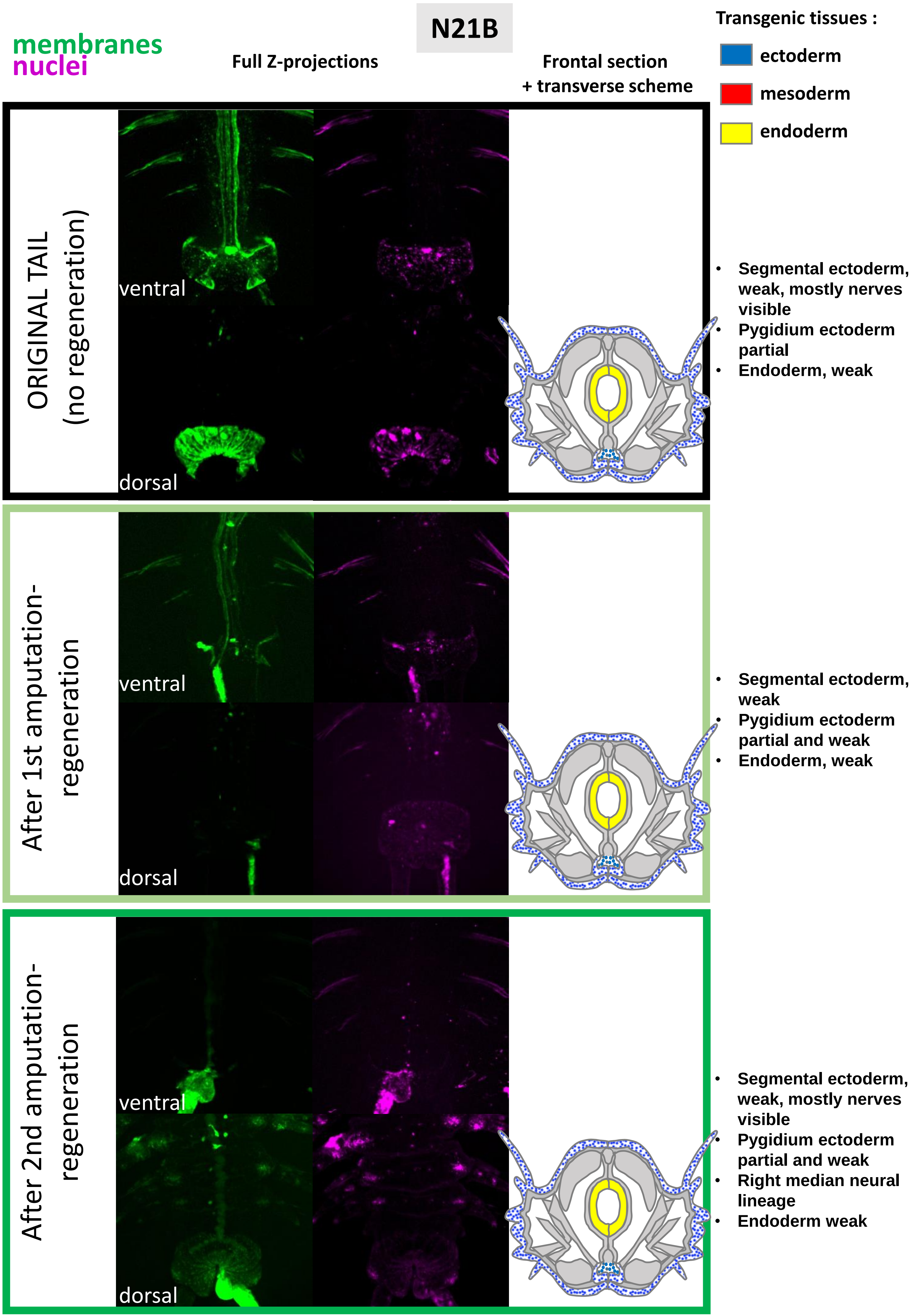
N21B
membranes
nuclei
Full Z-projections
Frontal section
+ transverse scheme
Transgenic tissues :
ectoderm
mesoderm
endoderm
ORIGINAL TAIL
(no regeneration)
ventral
dorsal
Segmental ectoderm, weak, mostly nerves visible
Pygidium ectoderm partial
Endoderm, weak
After 1st amputation-regeneration
ventral
dorsal
Segmental ectoderm, weak
Pygidium ectoderm partial and weak
Endoderm, weak
After 2nd amputation-regeneration
ventral
dorsal
Segmental ectoderm, weak, mostly nerves visible
Pygidium ectoderm partial and weak
Right median neural lineage
Endoderm weak

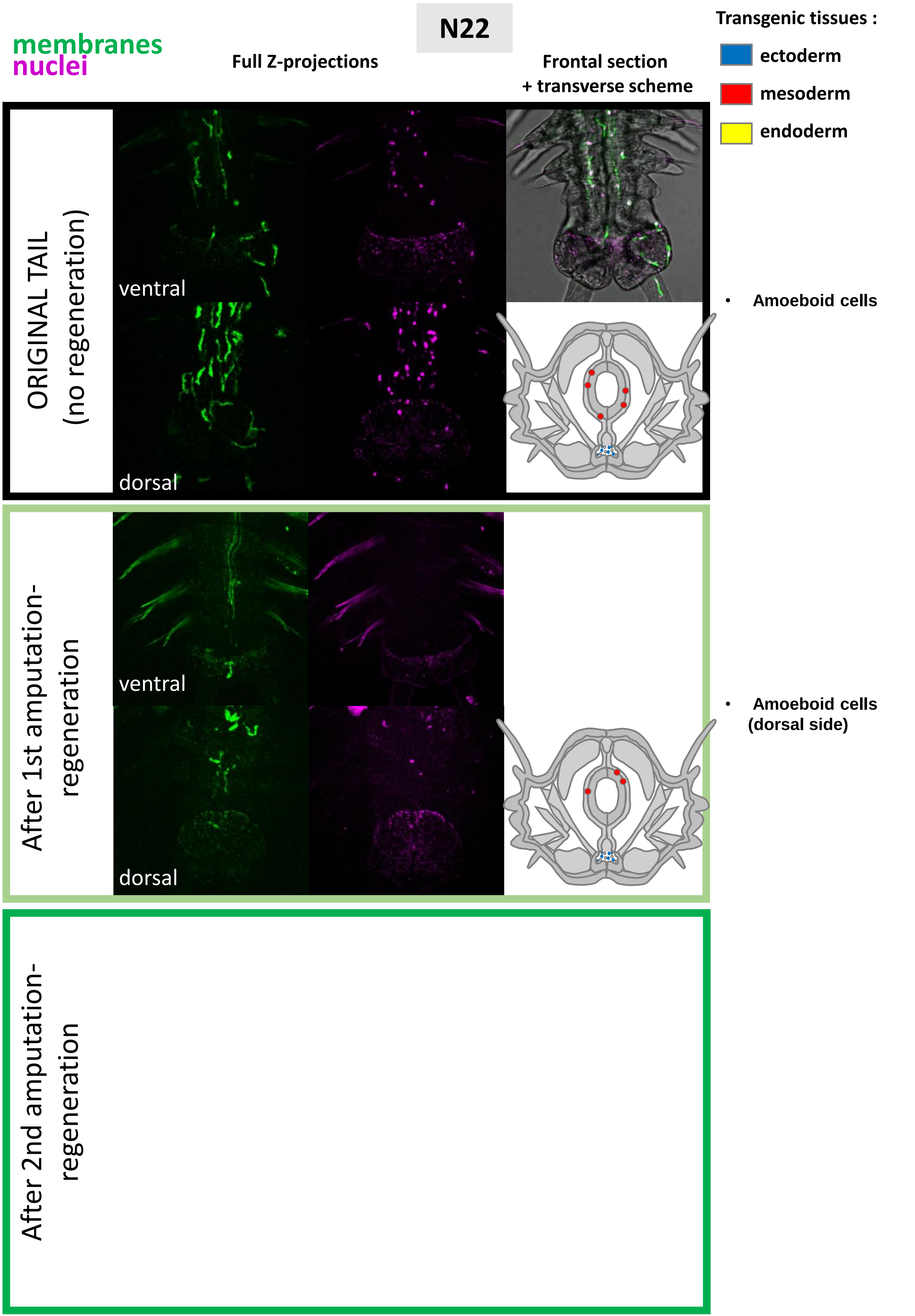
N22
membranes
nuclei
Full Z-projections
Frontal section
+ transverse scheme
Transgenic tissues :
ectoderm
mesoderm
endoderm
ORIGINAL TAIL
(no regeneration)
ventral
dorsal
• Amoeboid cells
After 1st amputation-
regeneration
ventral
dorsal
• Amoeboid cells
(dorsal side)
After 2nd amputation-
regeneration

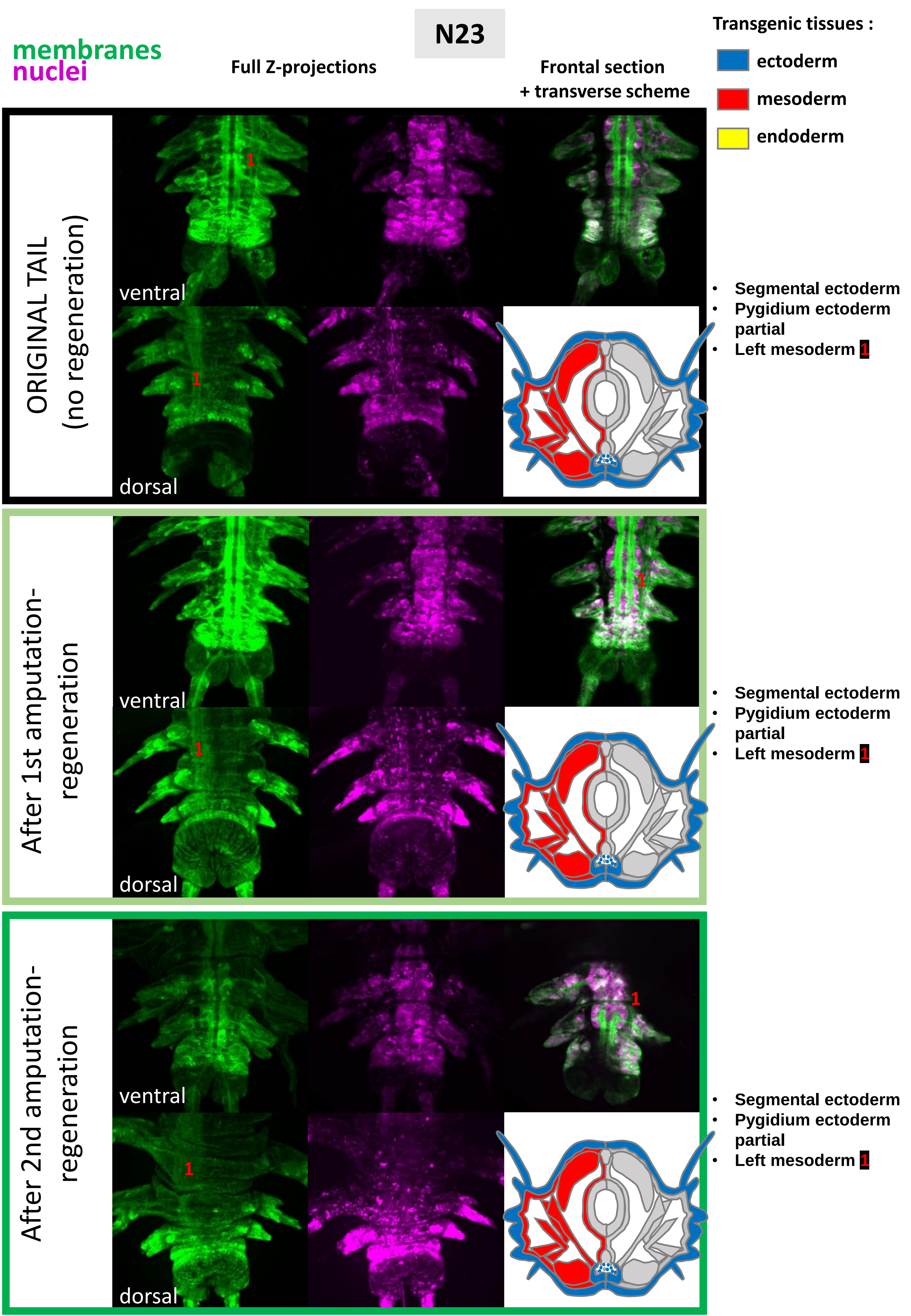
N23
membranes
nuclei
Full Z-projections
Frontal section
+ transverse scheme
Transgenic tissues :
ectoderm
mesoderm
endoderm
ORIGINAL TAIL
(no regeneration)
ventral
dorsal
1
• Segmental ectoderm
• Pygidium ectoderm partial
• Left mesoderm 1
After 1st amputation-
regeneration
ventral
dorsal
1
• Segmental ectoderm
• Pygidium ectoderm partial
• Left mesoderm 1
After 2nd amputation-
regeneration
ventral
dorsal
1
• Segmental ectoderm
• Pygidium ectoderm partial
• Left mesoderm 1

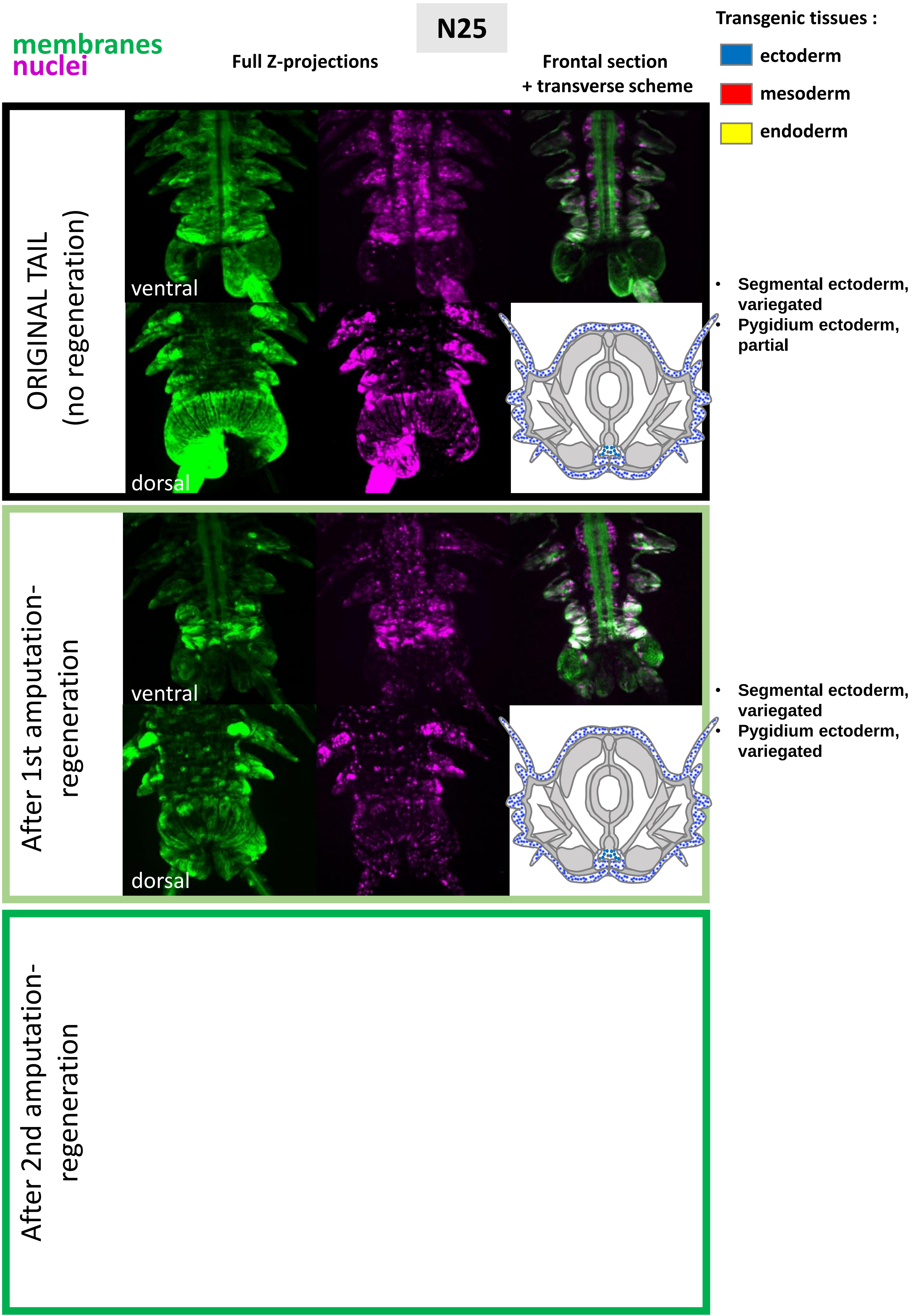
N25
membranes
nuclei
Full Z-projections
Frontal section
+ transverse scheme
Transgenic tissues :
ectoderm
mesoderm
endoderm
ORIGINAL TAIL
(no regeneration)
ventral
dorsal
• Segmental ectoderm, variegated
• Pygidium ectoderm, partial
After 1st amputation-regeneration
ventral
dorsal
• Segmental ectoderm, variegated
• Pygidium ectoderm, variegated
After 2nd amputation-regeneration

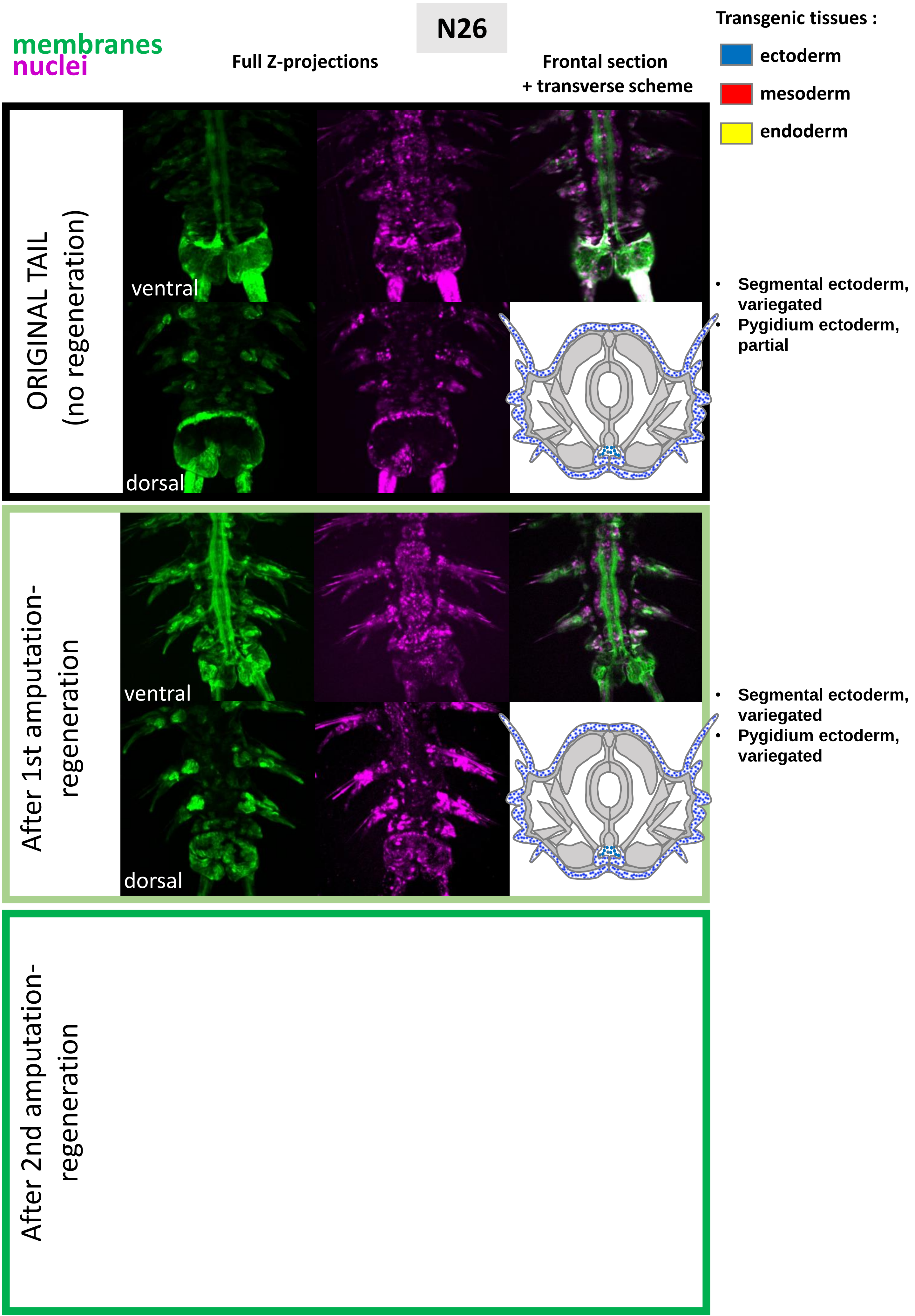

N26
membranes
nuclei
Full Z-projections
Frontal section
+ transverse scheme
Transgenic tissues :
ectoderm
mesoderm
endoderm
ORIGINAL TAIL
(no regeneration)
ventral
dorsal
Segmental ectoderm, variegated
Pygidium ectoderm, partial
After 1st amputation-regeneration
ventral
dorsal
Segmental ectoderm, variegated
Pygidium ectoderm, variegated
After 2nd amputation-regeneration

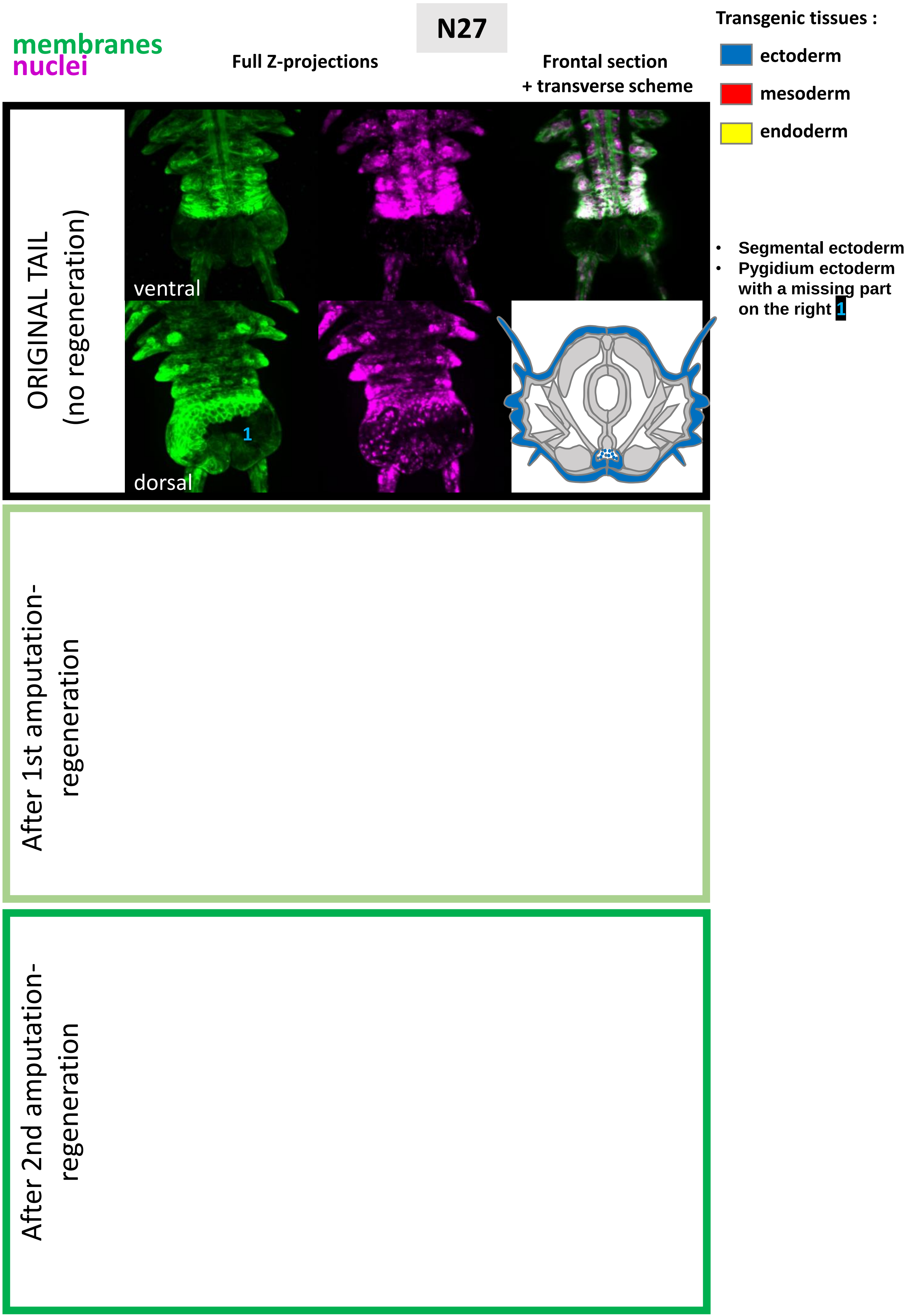
N27
membranes
nuclei
Full Z-projections
Frontal section
+ transverse scheme
Transgenic tissues :
ectoderm
mesoderm
endoderm
ORIGINAL TAIL
(no regeneration)
ventral
dorsal
1
• Segmental ectoderm
• Pygidium ectoderm with a missing part on the right 1
After 1st amputation-regeneration
After 2nd amputation-regeneration

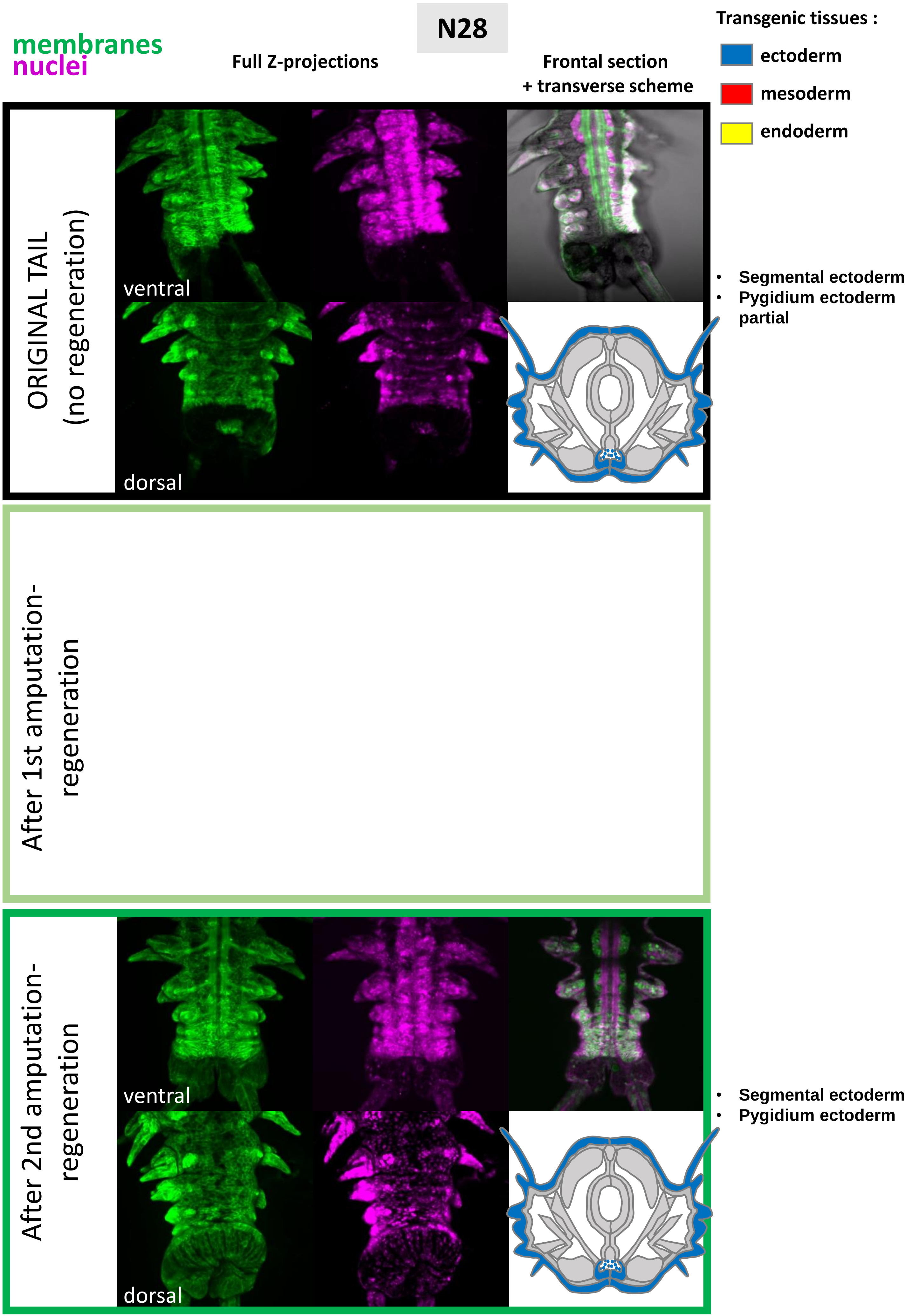
N28
membranes
nuclei
Full Z-projections
Frontal section
+ transverse scheme
Transgenic tissues :
ectoderm
mesoderm
endoderm
ORIGINAL TAIL
(no regeneration)
ventral
dorsal
• Segmental ectoderm
• Pygidium ectoderm partial
After 1st amputation-regeneration
After 2nd amputation-regeneration
ventral
dorsal
• Segmental ectoderm
• Pygidium ectoderm

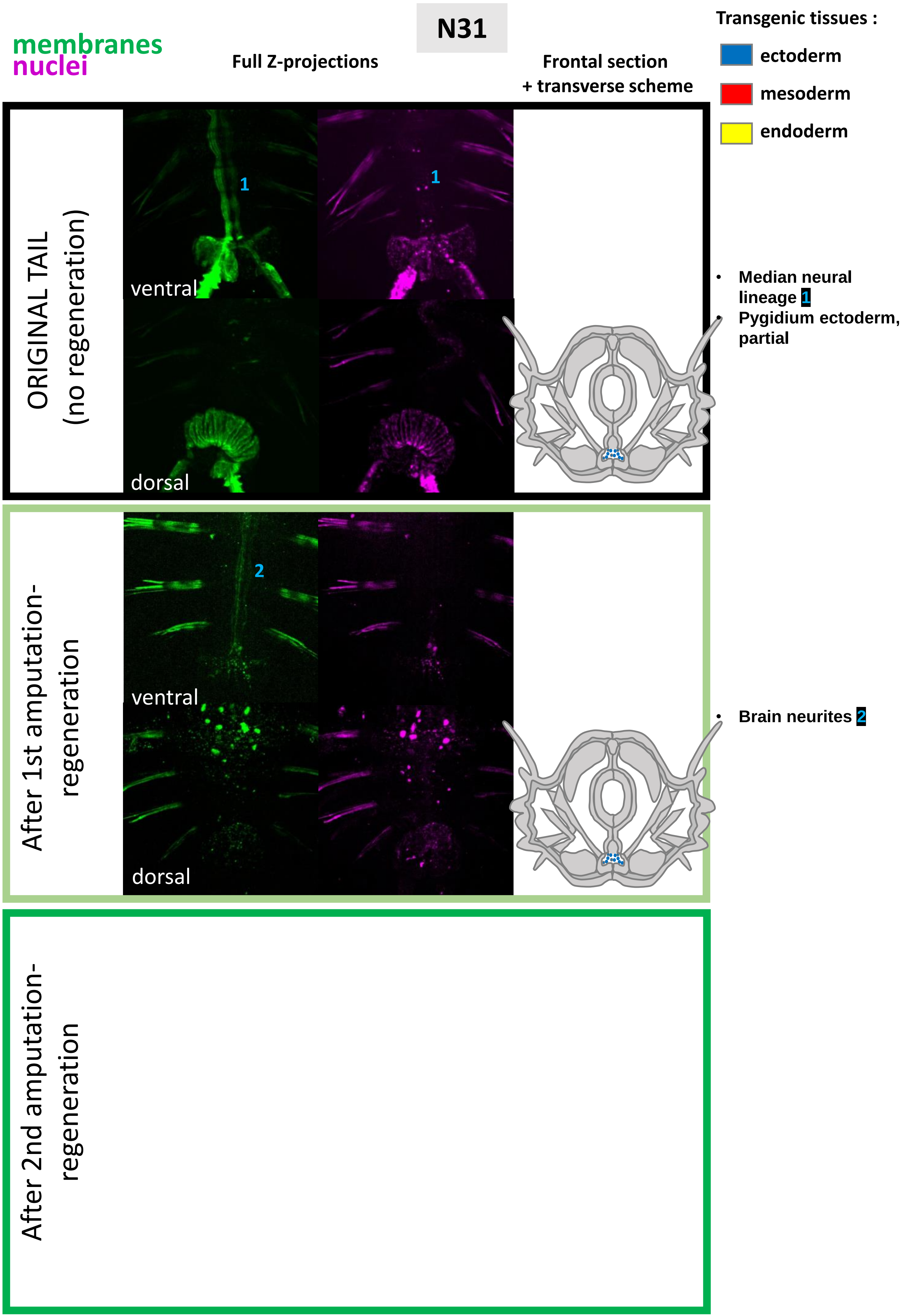
N31
membranes
nuclei
Full Z-projections
Frontal section
+ transverse scheme
Transgenic tissues :
ectoderm
mesoderm
endoderm
ORIGINAL TAIL
(no regeneration)
ventral
dorsal
1
1
Median neural lineage 1
Pygidium ectoderm, partial
After 1st amputation-regeneration
2
ventral
dorsal
Brain neurites 2
After 2nd amputation-regeneration

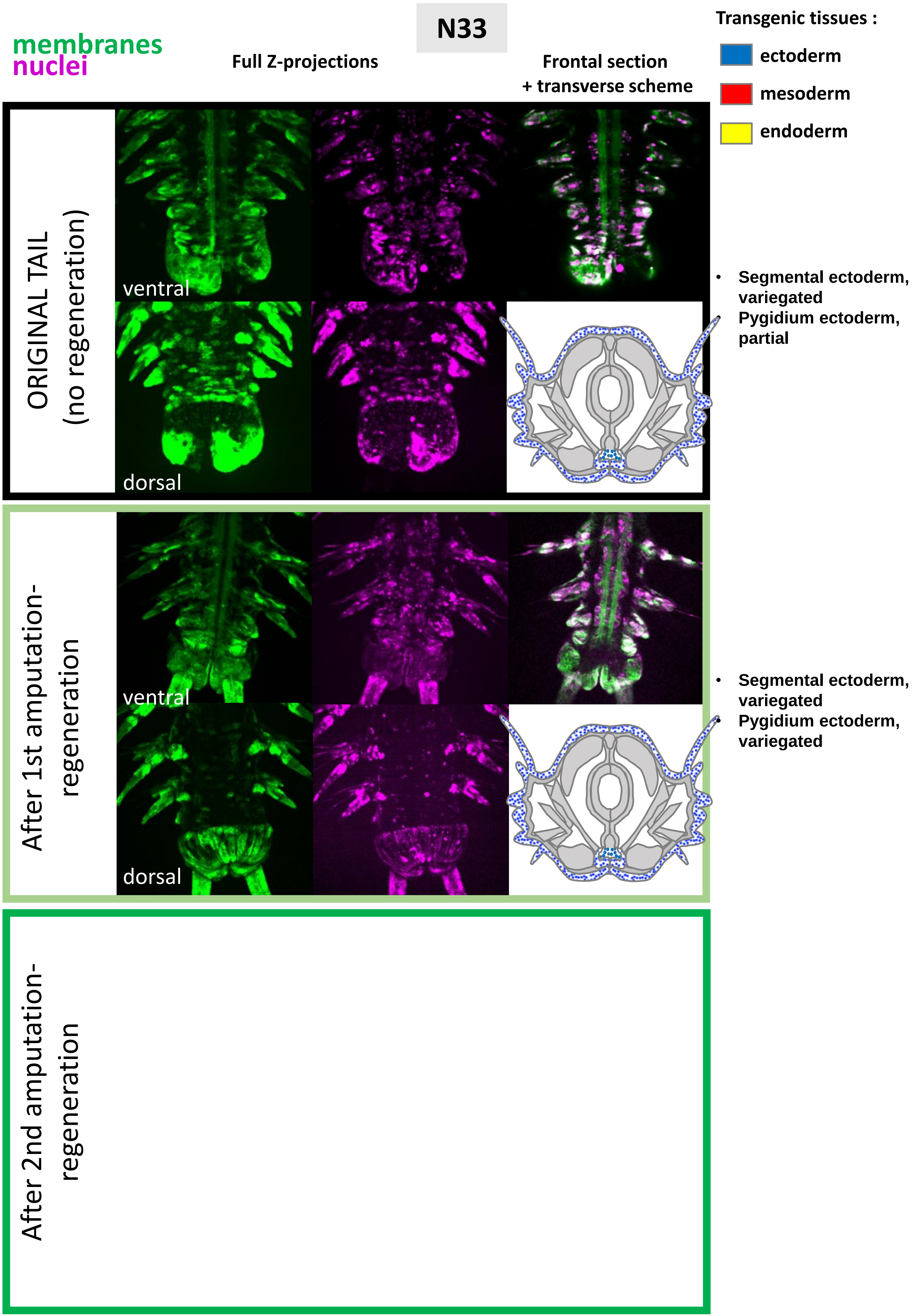
N33
membranes
nuclei
Full Z-projections
Frontal section
+ transverse scheme
Transgenic tissues :
ectoderm
mesoderm
endoderm
ORIGINAL TAIL
(no regeneration)
ventral
dorsal
• Segmental ectoderm, variegated
• Pygidium ectoderm, partial
After 1st amputation-regeneration
ventral
dorsal
• Segmental ectoderm, variegated
• Pygidium ectoderm, variegated
After 2nd amputation-regeneration

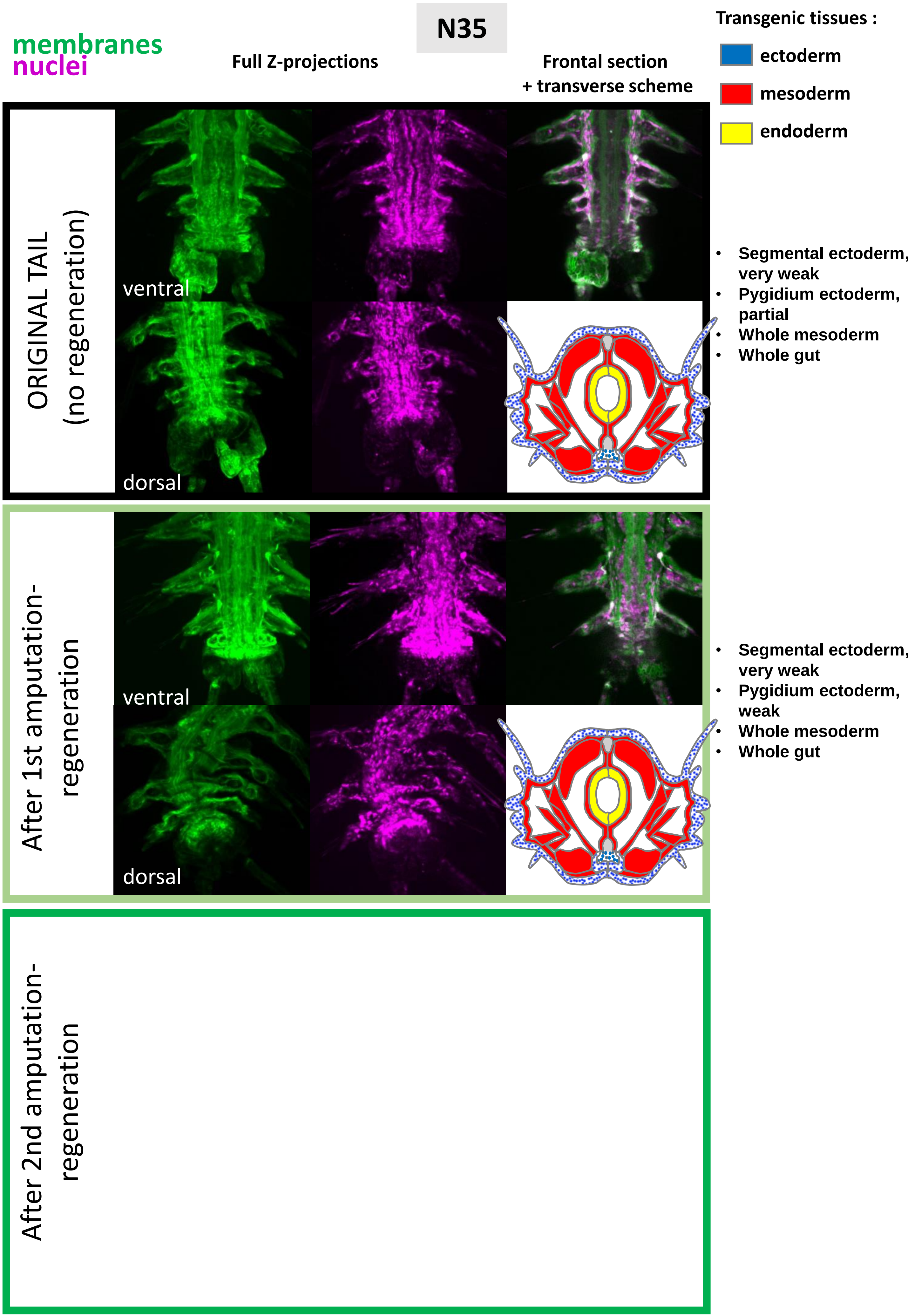

N35
membranes
nuclei
Full Z-projections
Frontal section
+ transverse scheme
Transgenic tissues :
ectoderm
mesoderm
endoderm
ORIGINAL TAIL
(no regeneration)
ventral
dorsal
• Segmental ectoderm, very weak
• Pygidium ectoderm, partial
• Whole mesoderm
• Whole gut
After 1st amputation-regeneration
ventral
dorsal
• Segmental ectoderm, very weak
• Pygidium ectoderm, weak
• Whole mesoderm
• Whole gut
After 2nd amputation-regeneration

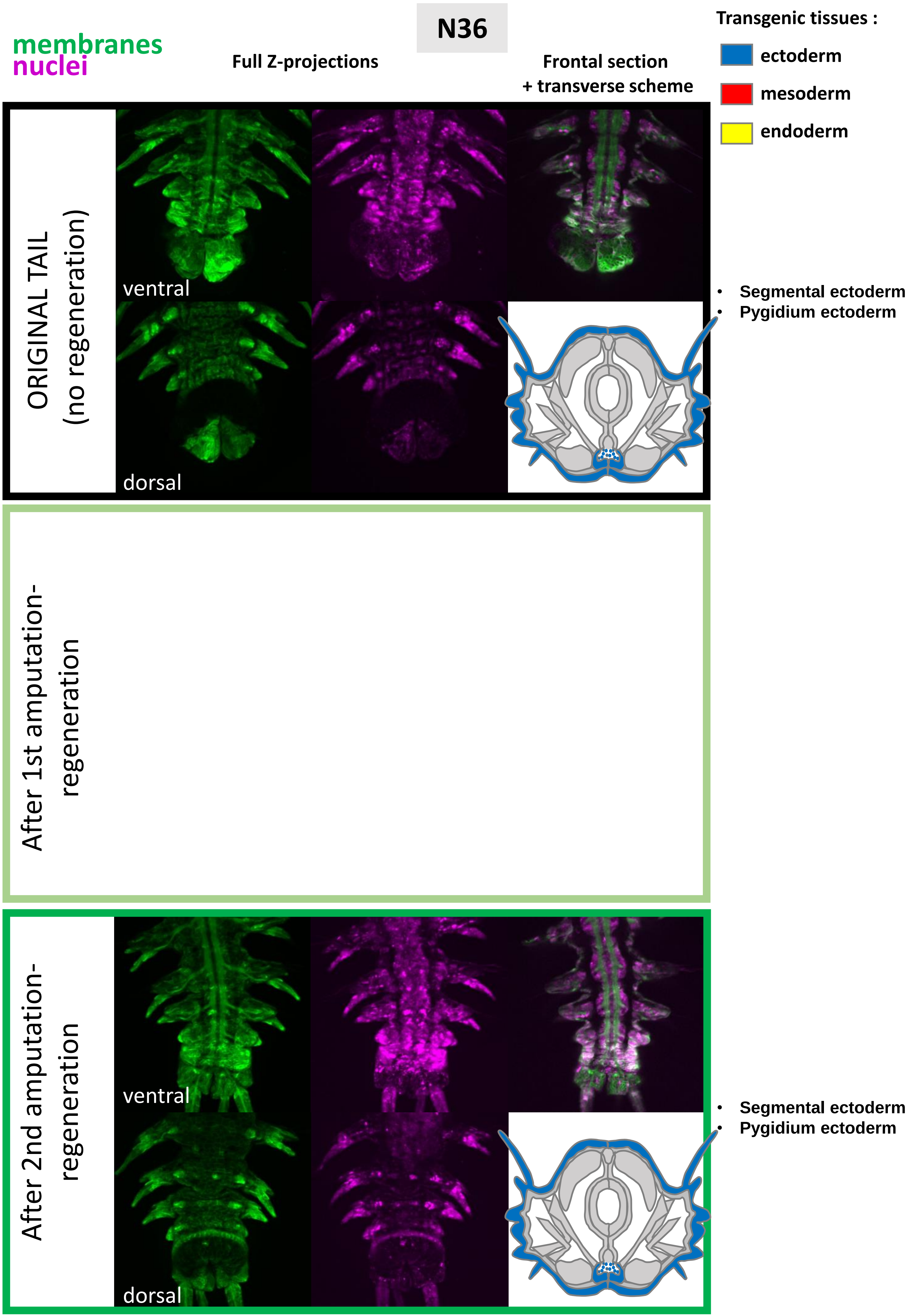
membranes
nuclei
N36
Full Z-projections
Frontal section
+ transverse scheme
Transgenic tissues :
ectoderm
mesoderm
endoderm
ORIGINAL TAIL
(no regeneration)
ventral
dorsal
Segmental ectoderm
Pygidium ectoderm
After 1st amputation-
regeneration
After 2nd amputation-
regeneration
ventral
dorsal
Segmental ectoderm
Pygidium ectoderm

membranes
nuclei

Full Z-projections

N38

Frontal section
+ transverse scheme

Transgenic tissues :
- ectoderm
- mesoderm
- endoderm

ORIGINAL TAIL
(no regeneration)

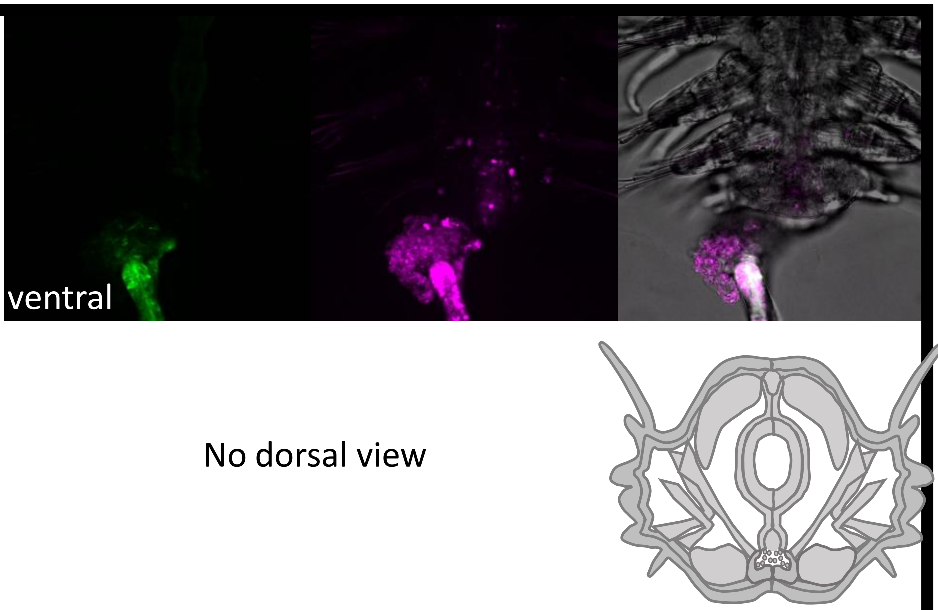

No dorsal view

- **Pygidium ectoderm, partial**

After 1st amputation-
regeneration

After 2nd amputation-
regeneration

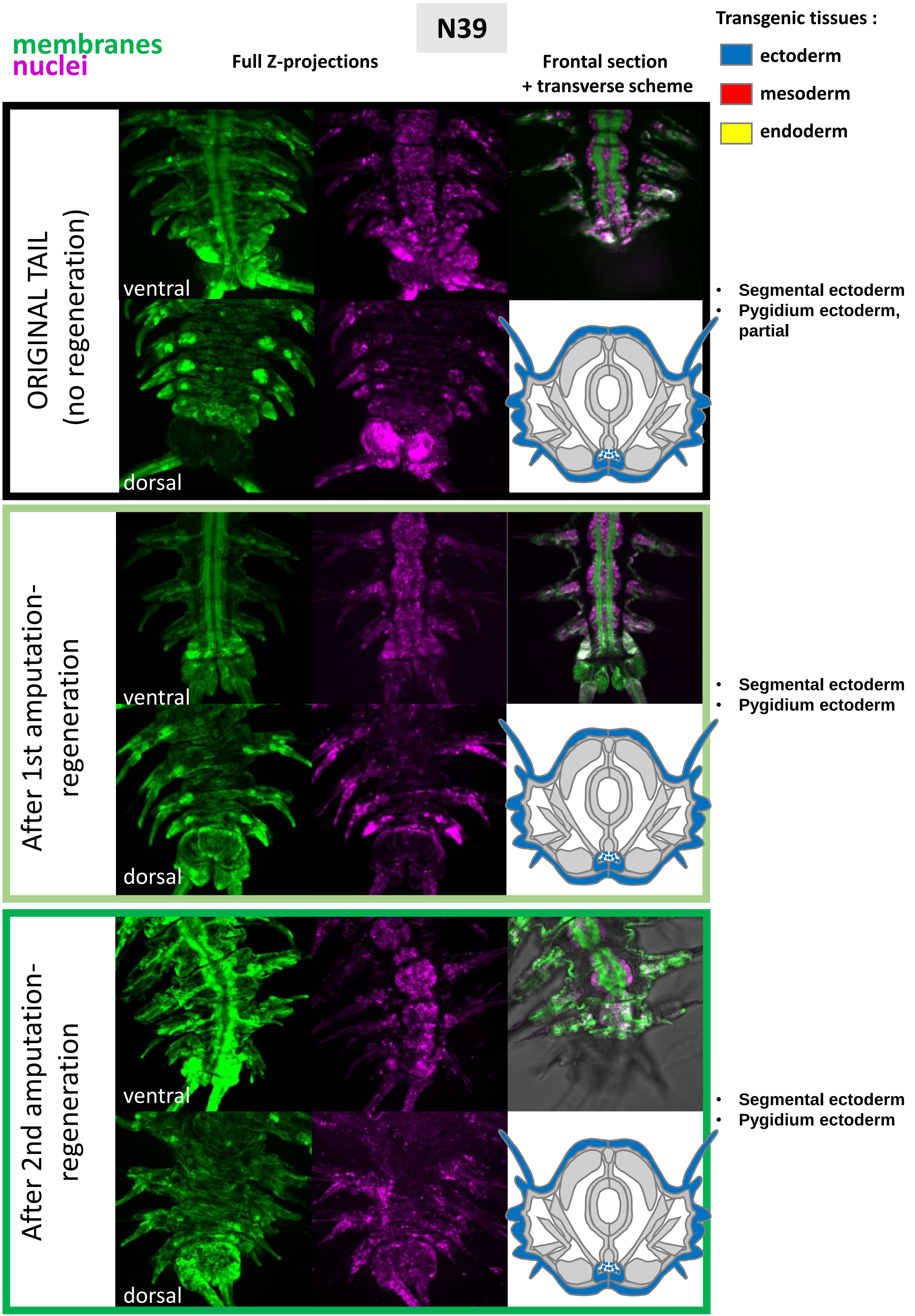
N39
membranes
nuclei
Full Z-projections
Frontal section
+ transverse scheme
Transgenic tissues :
ectoderm
mesoderm
endoderm
ORIGINAL TAIL
(no regeneration)
ventral
dorsal
• Segmental ectoderm
• Pygidium ectoderm, partial
After 1st amputation-regeneration
ventral
dorsal
• Segmental ectoderm
• Pygidium ectoderm
After 2nd amputation-regeneration
ventral
dorsal
• Segmental ectoderm
• Pygidium ectoderm

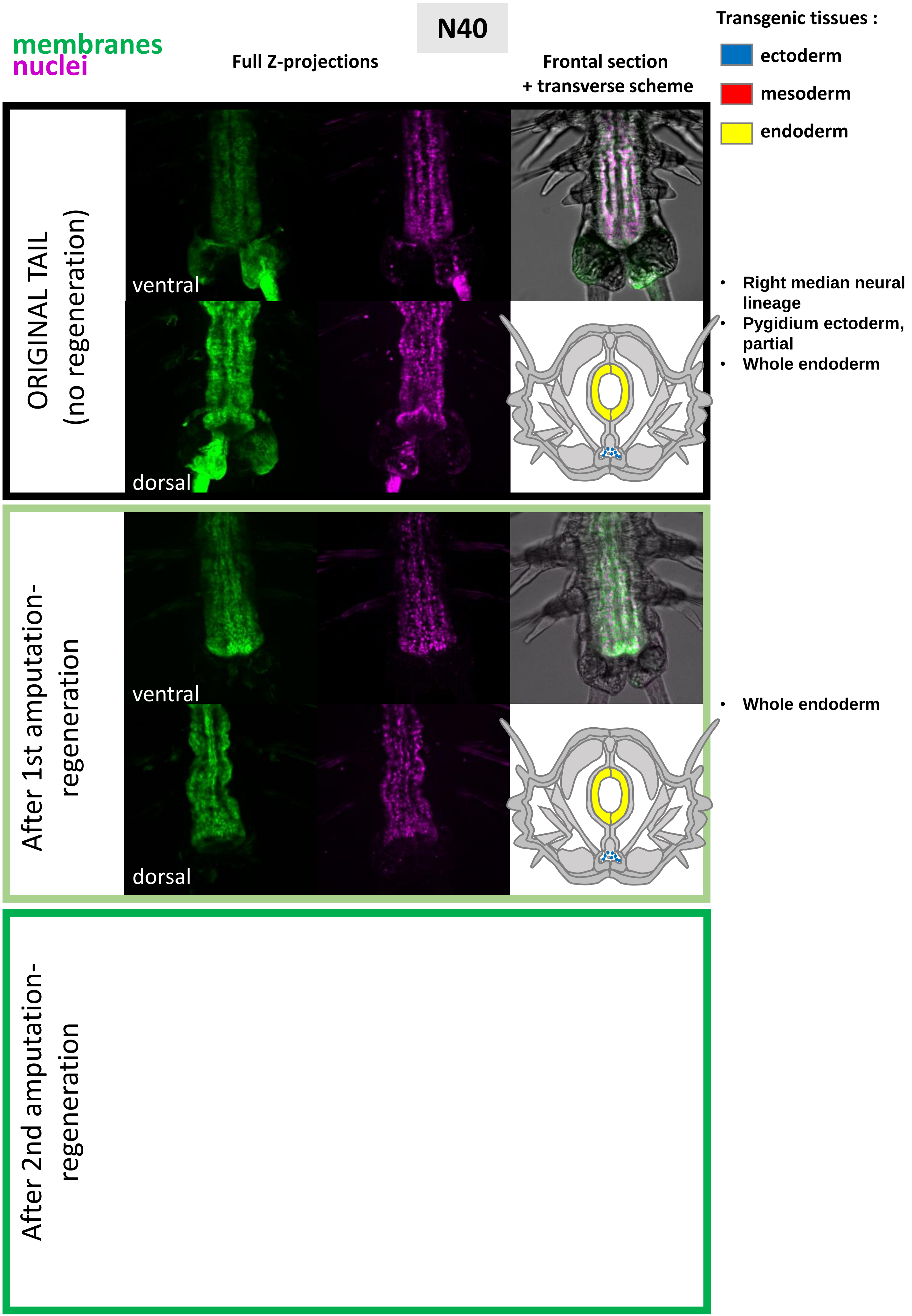

N40
membranes
nuclei
Full Z-projections
Frontal section
+ transverse scheme
Transgenic tissues :
ectoderm
mesoderm
endoderm
ORIGINAL TAIL
(no regeneration)
ventral
dorsal
• Right median neural lineage
• Pygidium ectoderm, partial
• Whole endoderm
After 1st amputation-regeneration
ventral
dorsal
• Whole endoderm
After 2nd amputation-regeneration

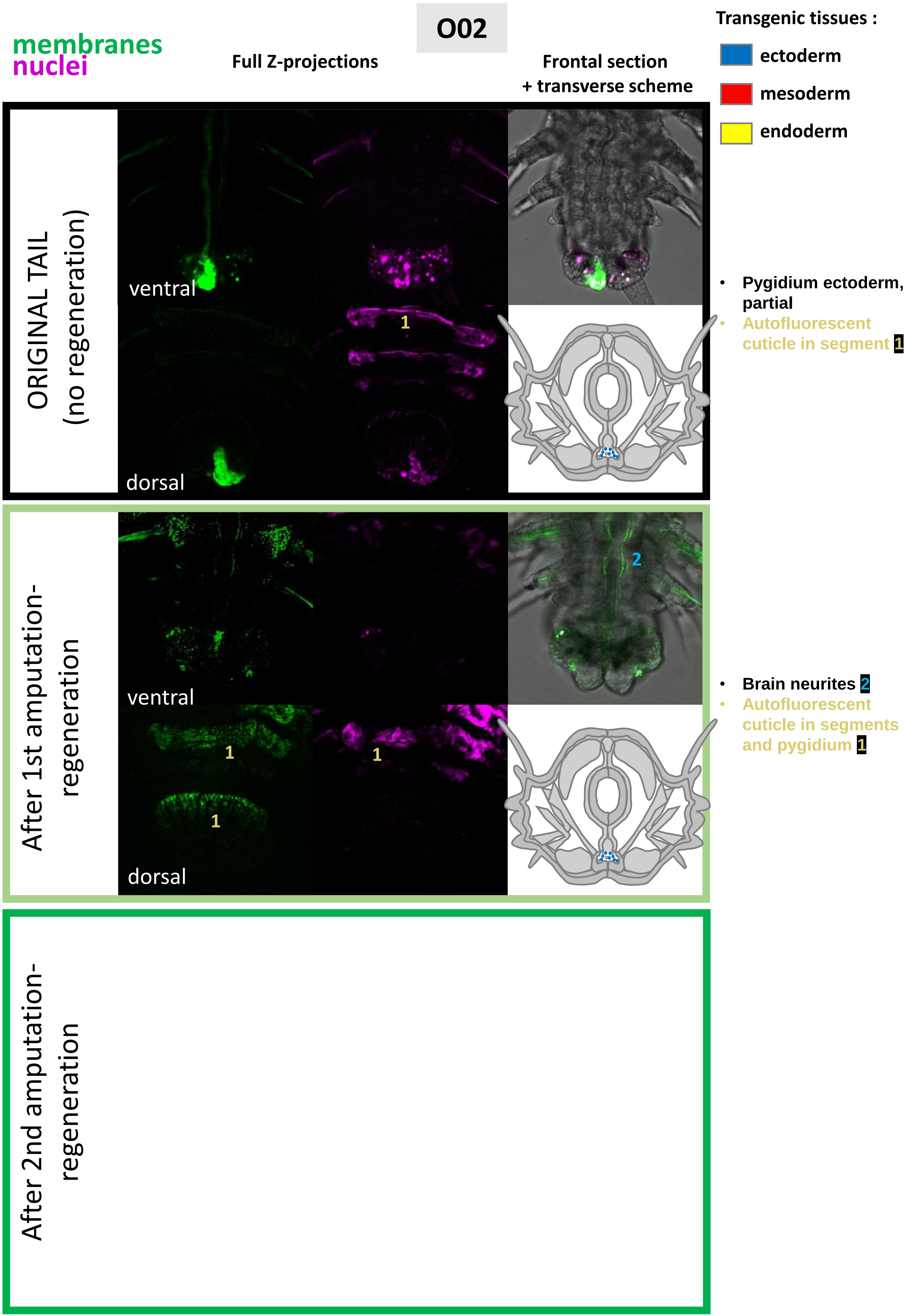

O02
membranes
nuclei
Full Z-projections
Frontal section
+ transverse scheme
Transgenic tissues :
ectoderm
mesoderm
endoderm
ORIGINAL TAIL
(no regeneration)
ventral
dorsal
1
• Pygidium ectoderm, partial
• Autofluorescent cuticle in segment 1
After 1st amputation-regeneration
2
1
• Brain neurites 2
• Autofluorescent cuticle in segments and pygidium 1
After 2nd amputation-regeneration

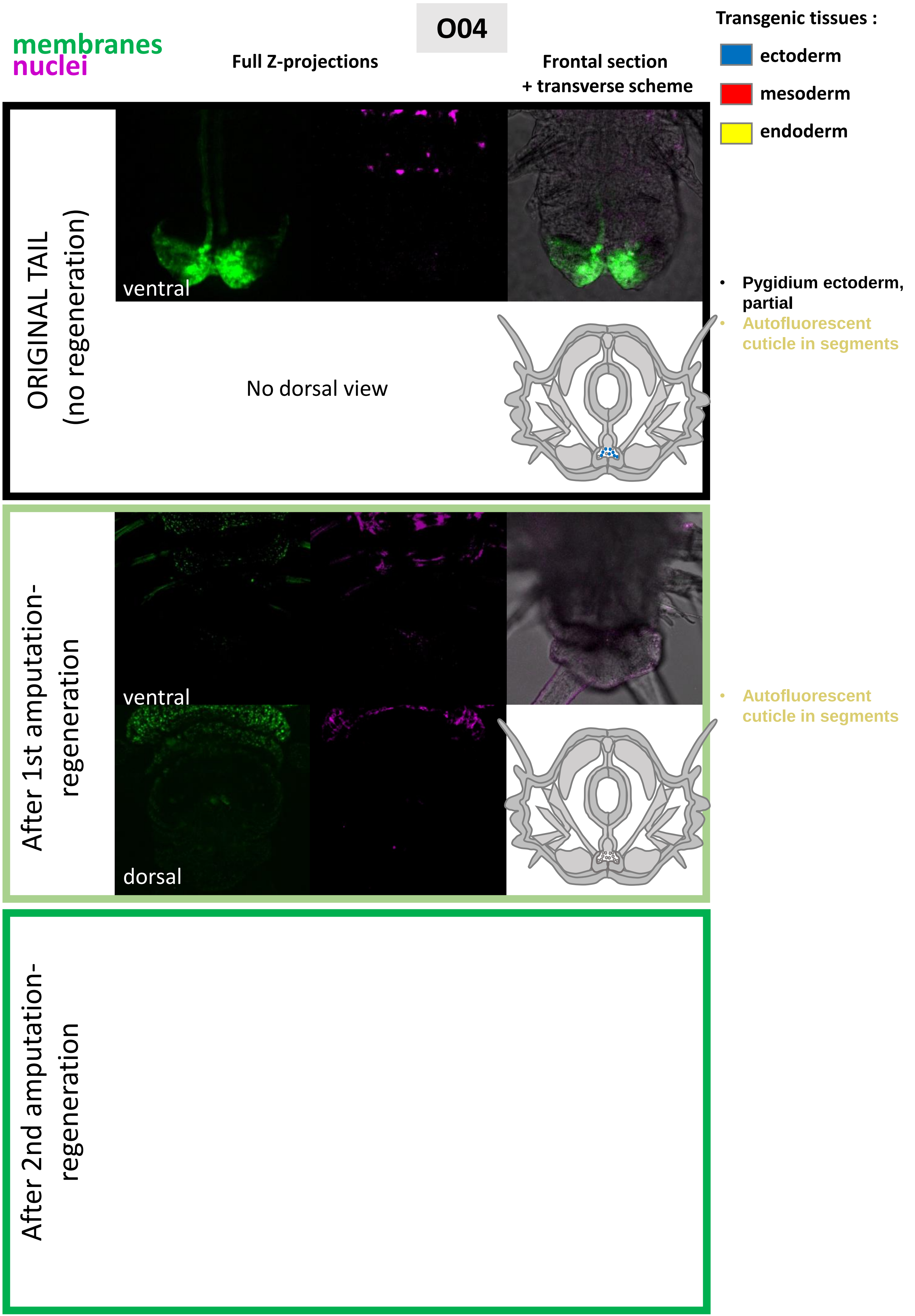
O04
membranes
nuclei
Full Z-projections
Frontal section
+ transverse scheme
Transgenic tissues :
ectoderm
mesoderm
endoderm
ORIGINAL TAIL
(no regeneration)
ventral
No dorsal view
• Pygidium ectoderm, partial
• Autofluorescent cuticle in segments
After 1st amputation-regeneration
ventral
dorsal
• Autofluorescent cuticle in segments
After 2nd amputation-regeneration

membranes
nuclei

Full Z-projections

O06

Frontal section
+ transverse scheme

Transgenic tissues :

- ectoderm
- mesoderm
- endoderm

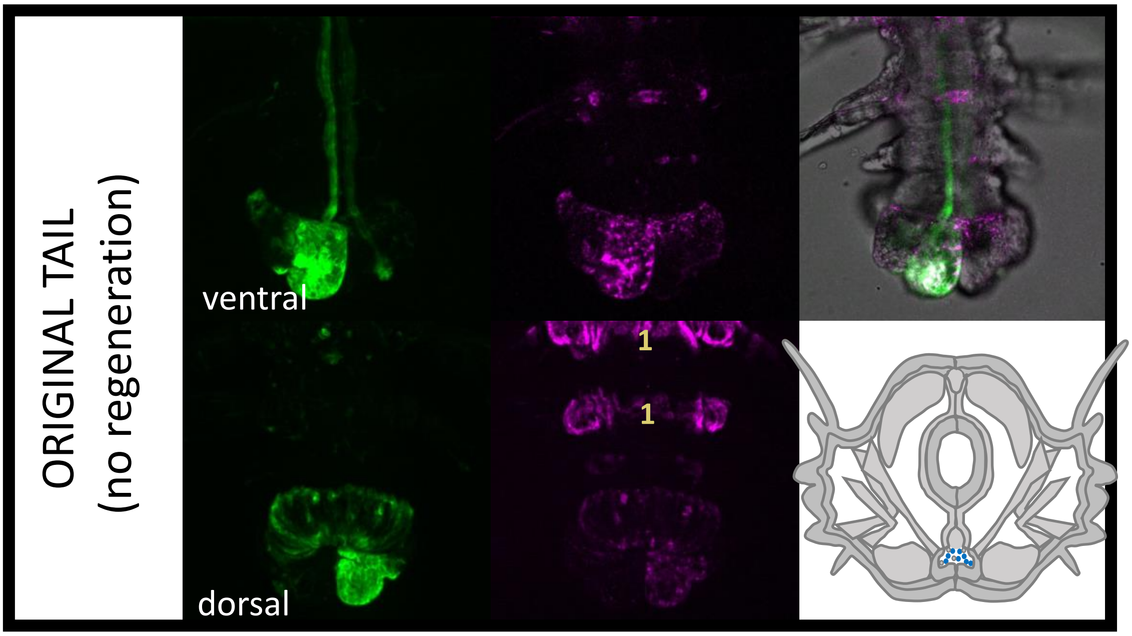

- **Pygidium ectoderm, partial**
- Autofluorescent cuticle in segments 1

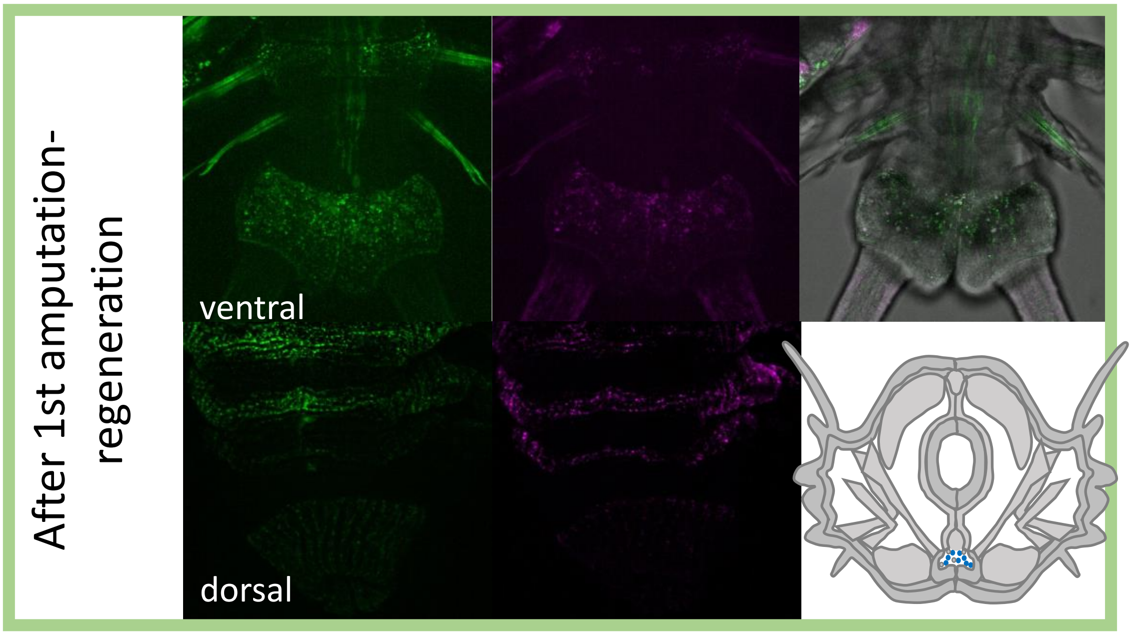

- Autofluorescent cuticle in segments and pygidium

After 2nd amputation-
regeneration

O07

membranes
nuclei

Full Z-projections

Frontal section
+ transverse scheme

Transgenic tissues :

- ectoderm
- mesoderm
- endoderm

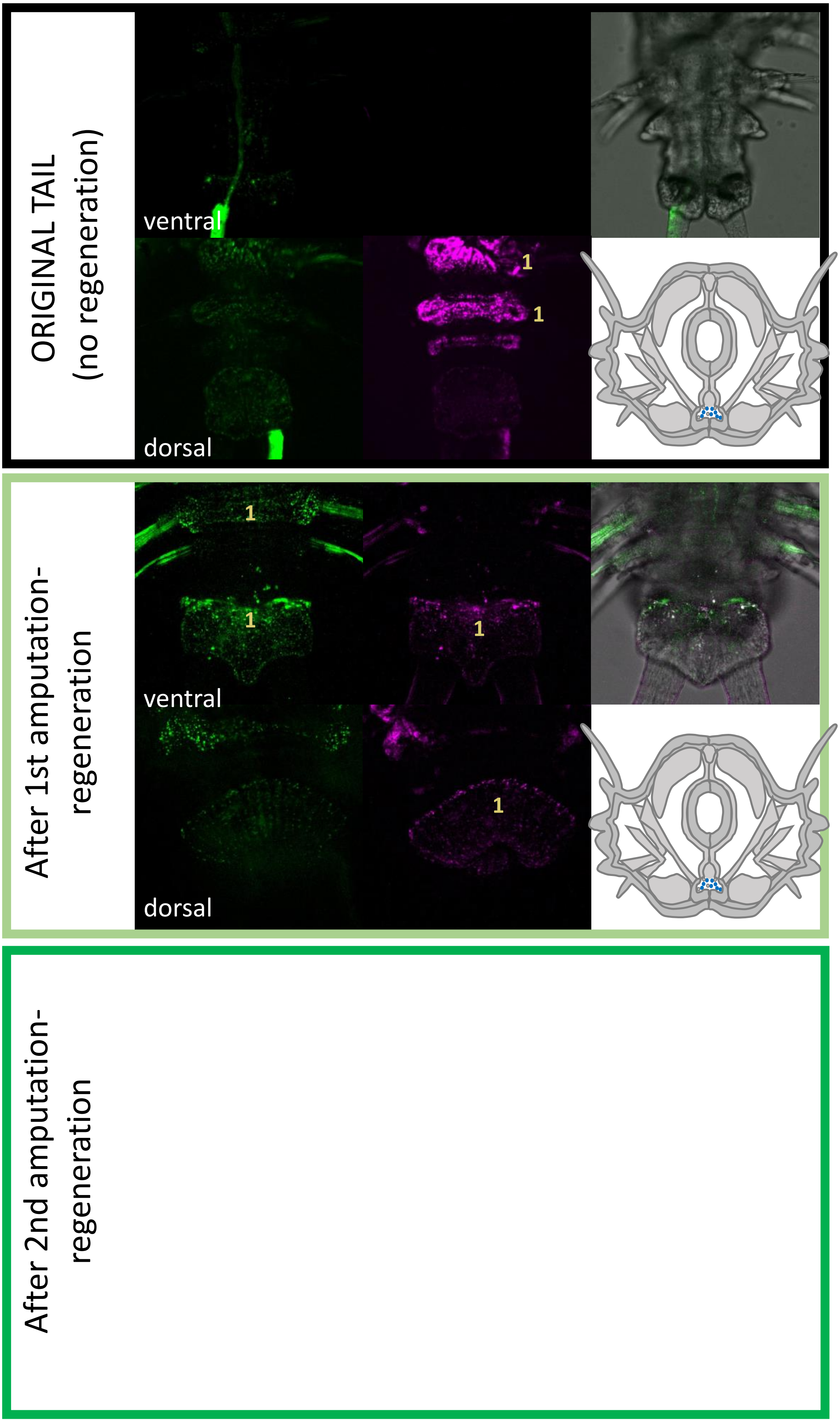

- **Pygidium ectoderm, partial, right cirrus**
- **Autofluorescent cuticle in segments** 1

- **Autofluorescent cuticle in segments and pygidium** 1

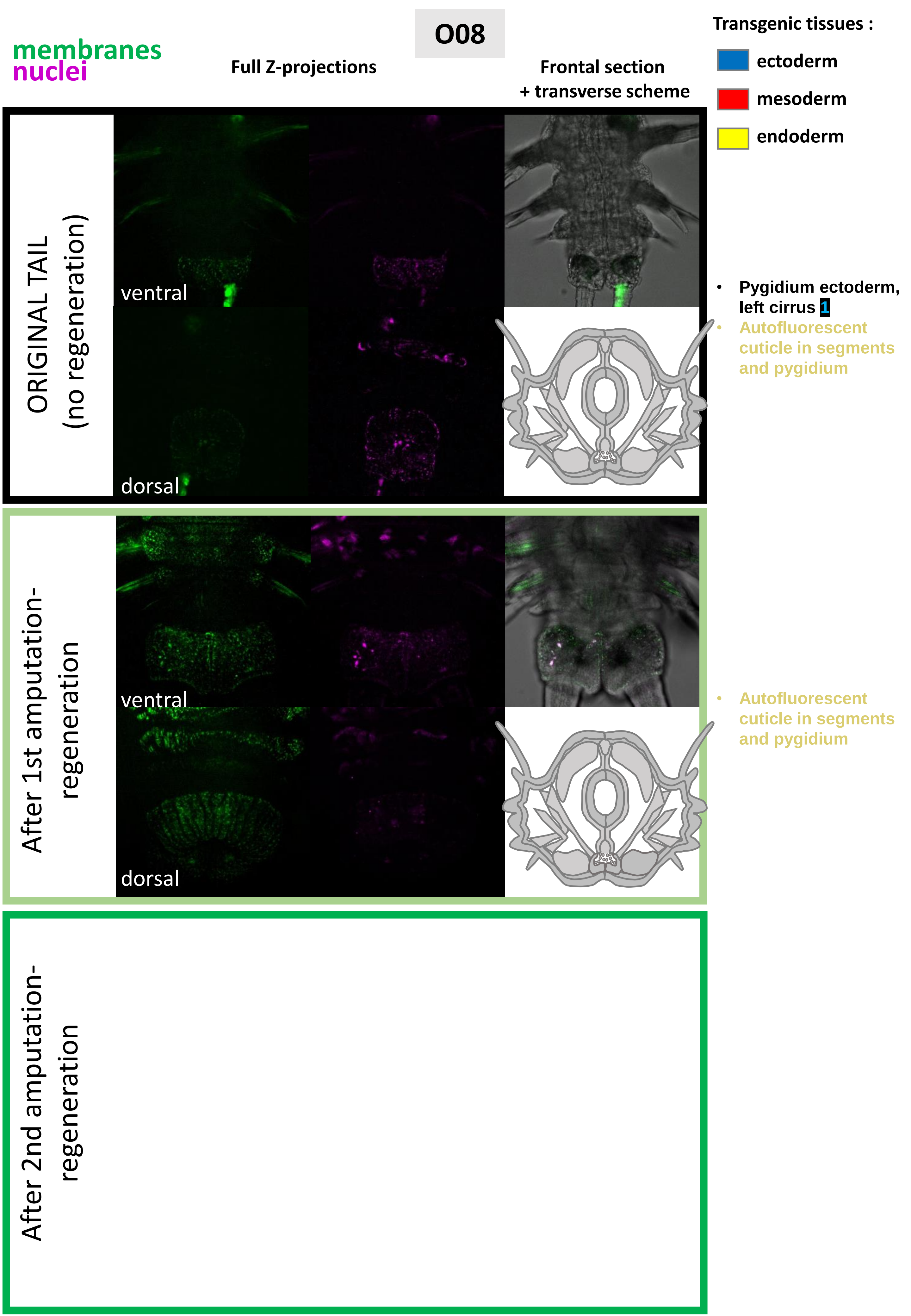
O08
membranes
nuclei
Full Z-projections
Frontal section
+ transverse scheme
Transgenic tissues :
ectoderm
mesoderm
endoderm
ORIGINAL TAIL
(no regeneration)
ventral
dorsal
• Pygidium ectoderm, left cirrus 1
• Autofluorescent cuticle in segments and pygidium
After 1st amputation-regeneration
ventral
dorsal
• Autofluorescent cuticle in segments and pygidium
After 2nd amputation-regeneration

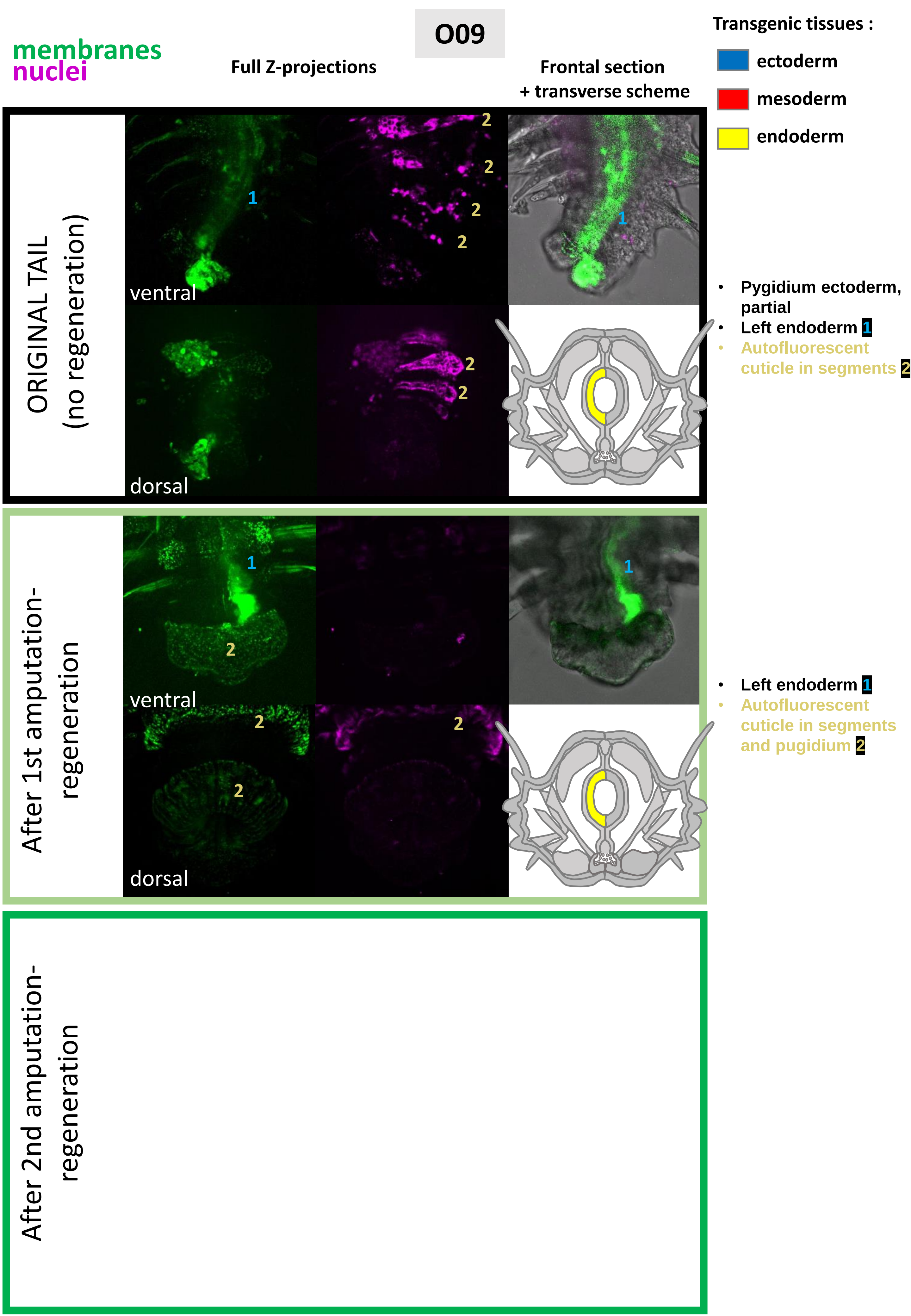
O09
membranes
nuclei
Full Z-projections
Frontal section
+ transverse scheme
Transgenic tissues :
ectoderm
mesoderm
endoderm
ORIGINAL TAIL
(no regeneration)
ventral
dorsal
1
2
• Pygidium ectoderm, partial
• Left endoderm 1
• Autofluorescent cuticle in segments 2
After 1st amputation-regeneration
• Left endoderm 1
• Autofluorescent cuticle in segments and pugidium 2
After 2nd amputation-regeneration

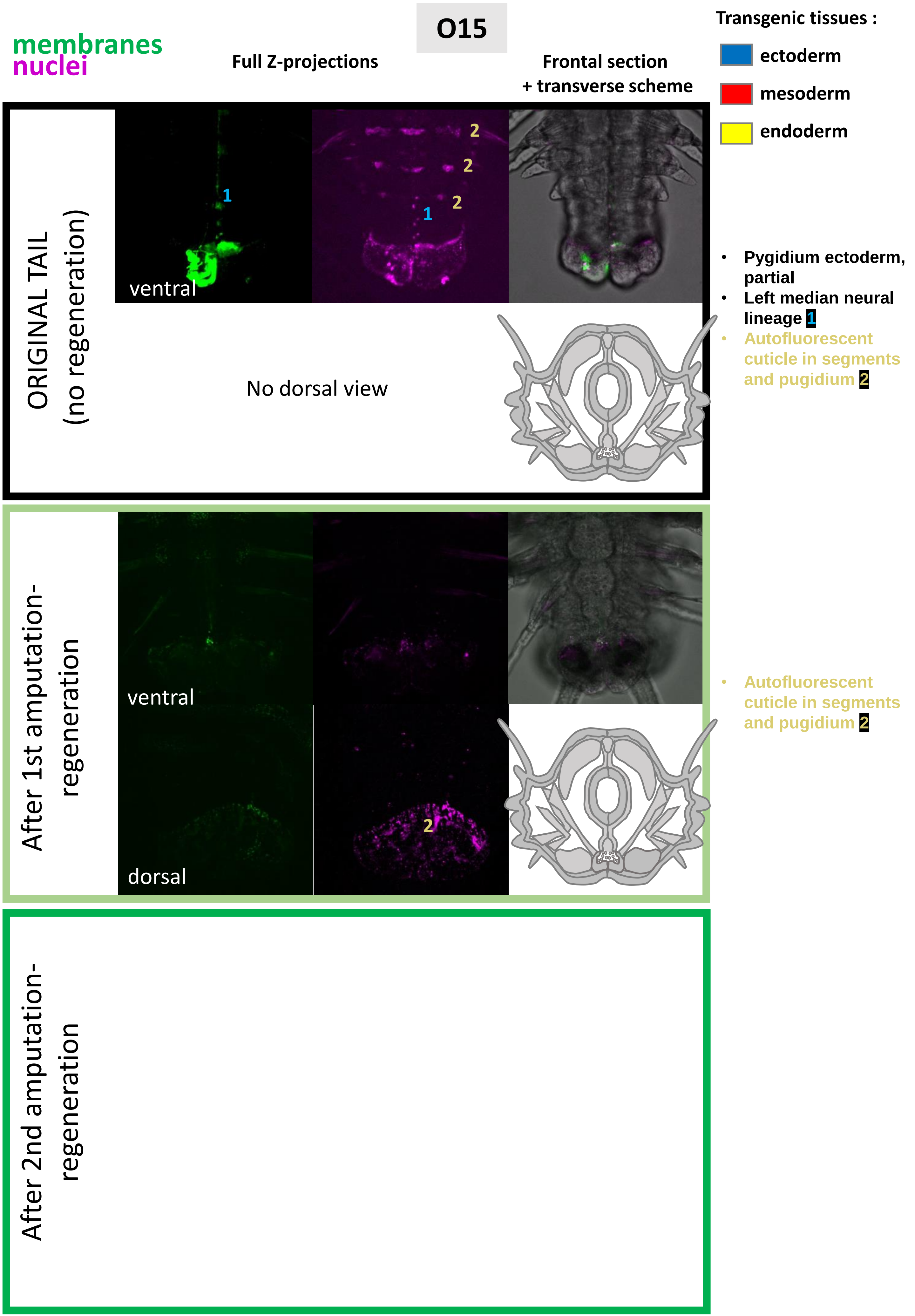
O15
membranes
nuclei
Full Z-projections
Frontal section
+ transverse scheme
Transgenic tissues :
ectoderm
mesoderm
endoderm
ORIGINAL TAIL
(no regeneration)
ventral
1
2
No dorsal view
• Pygidium ectoderm, partial
• Left median neural lineage 1
• Autofluorescent cuticle in segments and pugidium 2
After 1st amputation-regeneration
ventral
dorsal
• Autofluorescent cuticle in segments and pugidium 2
After 2nd amputation-regeneration

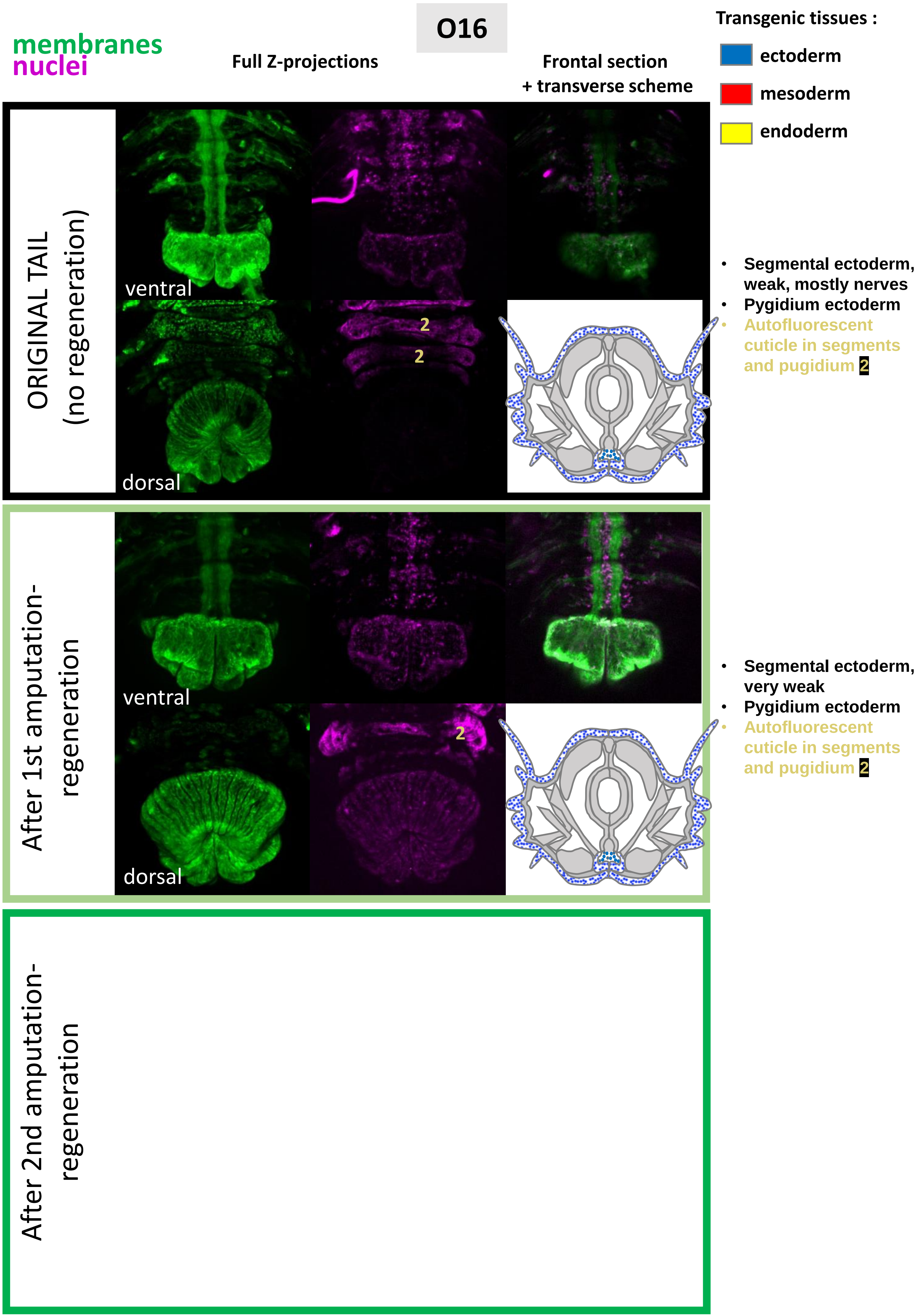
O16
membranes
nuclei
Full Z-projections
Frontal section
+ transverse scheme
Transgenic tissues :
ectoderm
mesoderm
endoderm
ORIGINAL TAIL
(no regeneration)
ventral
dorsal
2
2
Segmental ectoderm, weak, mostly nerves
Pygidium ectoderm
Autofluorescent cuticle in segments and pugidium 2
After 1st amputation-regeneration
ventral
dorsal
2
Segmental ectoderm, very weak
Pygidium ectoderm
Autofluorescent cuticle in segments and pugidium 2
After 2nd amputation-regeneration

Supplementary data file 7, transgenic clones, part C

Legend: data absent | ubiquitous expression | stochastic expression | partially transgenic

animal ID	segmental ectoderm	median neural	pygidial ectoderm	segmental mesoderm	pygidial mesoderm	endoderm	amoeboid cells
M01 primary	data absent	data absent	data absent	data absent	data absent	data absent	data absent
M01 1st reg.	ubiquitous expression		ubiquitous expression			ubiquitous expression	
M01 2nd reg.	ubiquitous expression		ubiquitous expression			ubiquitous expression	
M03 primary	data absent	data absent	data absent	data absent	data absent	data absent	data absent
M03 1st reg.				ubiquitous expression	ubiquitous expression		
M03 2nd reg.				ubiquitous expression	ubiquitous expression		
M05 primary	stochastic expression		partially transgenic			partially transgenic	
M05 1st reg.	stochastic expression		stochastic expression			partially transgenic	
M05 2nd reg.	stochastic expression		stochastic expression			partially transgenic	
M07 primary	stochastic expression						
M07 1st reg.	stochastic expression		ubiquitous expression				
M07 2nd reg.	stochastic expression		ubiquitous expression				
M08 primary			partially transgenic				
M08 1st reg.							
M08 2nd reg.	data absent	data absent	data absent	data absent	data absent	data absent	data absent
M09 primary		partially transgenic	partially transgenic				ubiquitous expression
M09 1st reg.							
M09 2nd reg.	data absent	data absent	data absent	data absent	data absent	data absent	data absent
M10 primary		partially transgenic	partially transgenic				
M10 1st reg.							
M10 2nd reg.	data absent	data absent	data absent	data absent	data absent	data absent	data absent
M11 primary	stochastic expression		partially transgenic			partially transgenic	
M11 1st reg.	stochastic expression		stochastic expression			partially transgenic	
M11 2nd reg.	stochastic expression		stochastic expression			partially transgenic	
M15 primary			partially transgenic			partially transgenic	
M15 1st reg.						partially transgenic	
M15 2nd reg.	data absent	data absent	data absent	data absent	data absent	data absent	data absent
M18 primary	stochastic expression		stochastic expression				
M18 1st reg.	data absent	data absent	data absent	data absent	data absent	data absent	data absent
M18 2nd reg.	data absent	data absent	data absent	data absent	data absent	data absent	data absent
M19 primary	stochastic expression		partially transgenic				
M19 1st reg.	data absent	data absent	data absent	data absent	data absent	data absent	data absent
M19 2nd reg.	stochastic expression		stochastic expression				
M20 primary	stochastic expression	ubiquitous expression	ubiquitous expression				
M20 1st reg.	data absent	data absent	data absent	data absent	data absent	data absent	data absent
M20 2nd reg.	data absent	data absent	data absent	data absent	data absent	data absent	data absent
M21 primary	stochastic expression		partially transgenic			stochastic expression	
M21 1st reg.	stochastic expression		stochastic expression			stochastic expression	
M21 2nd reg.	stochastic expression		stochastic expression			stochastic expression	
M22 primary	partially transgenic		partially transgenic				
M22 1st reg.	partially transgenic		partially transgenic				
M22 2nd reg.	data absent	data absent	data absent	data absent	data absent	data absent	data absent

animal ID	segmental ectoderm	median neural	pygidial ectoderm	segmental mesoderm	pygidial mesoderm	endoderm	amoeboid cells
M23 primary							
M23 1st reg.							
M23 2nd reg.							
M24 primary							
M24 1st reg.							
M24 2nd reg.							
M25 primary							
M25 1st reg.							
M25 2nd reg.							
M26 primary							
M26 1st reg.							
M26 2nd reg.							
M27 primary							
M27 1st reg.							
M27 2nd reg.							
M28 primary							
M28 1st reg.							
M28 2nd reg.							
M29 primary							
M29 1st reg.							
M29 2nd reg.							
M30 primary							
M30 1st reg.							
M30 2nd reg.							
N01 primary							
N01 1st reg.							
N01 2nd reg.							
N02 primary							
N02 1st reg.							
N02 2nd reg.							
N03 primary							
N03 1st reg.							
N03 2nd reg.							
N04 primary							
N04 1st reg.							
N04 2nd reg.							
N05 primary							
N05 1st reg.							
N05 2nd reg.							
N06 primary							
N06 1st reg.							
N06 2nd reg.							
N07 primary							
N07 1st reg.							
N07 2nd reg.							

animal ID	segmental ectoderm	median neural	pygidial ectoderm	segmental mesoderm	pygidial mesoderm	endoderm	amoeboid cells
N08 primary							
N08 1st reg.							
N08 2nd reg.							
N09 primary							
N09 1st reg.							
N09 2nd reg.							
N10 primary							
N10 1st reg.							
N10 2nd reg.							
N11 primary							
N11 1st reg.							
N11 2nd reg.							
N12 primary							
N12 1st reg.							
N12 2nd reg.							
N13 primary							
N13 1st reg.							
N13 2nd reg.							
N14 primary							
N14 1st reg.							
N14 2nd reg.							
N15 primary							
N15 1st reg.							
N15 2nd reg.							
N16 primary							
N16 1st reg.							
N16 2nd reg.							
N19 primary							
N19 1st reg.							
N19 2nd reg.							
N20 primary							
N20 1st reg.							
N20 2nd reg.							
N21A primary							
N21A 1st reg.							
N21A 2nd reg.							
N21B primary							
N21B 1st reg.							
N21B 2nd reg.							
N22 primary							
N22 1st reg.							
N22 2nd reg.							
N23 primary							
N23 1st reg.							
N23 2nd reg.							

animal ID	segmental ectoderm	median neural	pygidial ectoderm	segmental mesoderm	pygidial mesoderm	endoderm	amoeboid cells
N25 primary							
N25 1st reg.							
N25 2nd reg.							
N26 primary							
N26 1st reg.							
N26 2nd reg.							
N27 primary							
N27 1st reg.							
N27 2nd reg.							
N28 primary							
N28 1st reg.							
N28 2nd reg.							
N31 primary							
N31 1st reg.							
N31 2nd reg.							
N33 primary							
N33 1st reg.							
N33 2nd reg.							
N35 primary							
N35 1st reg.							
N35 2nd reg.							
N36 primary							
N36 1st reg.							
N36 2nd reg.							
N38 primary							
N38 1st reg.							
N38 2nd reg.							
N39 primary							
N39 1st reg.							
N39 2nd reg.							
N40 primary							
N40 1st reg.							
N40 2nd reg.							
O02 primary							
O02 1st reg.							
O02 2nd reg.							
O04 primary							
O04 1st reg.							
O04 2nd reg.							
O06 primary							
O06 1st reg.							
O06 2nd reg.							
O07 primary							
O07 1st reg.							
O07 2nd reg.							

animal ID	segmental ectoderm	median neural	pygidial ectoderm	segmental mesoderm	pygidial mesoderm	endoderm	amoeboid cells
O08 primary							
O08 1st reg.							
O08 2nd reg.							
O09 primary							
O09 1st reg.							
O09 2nd reg.							
O15 primary							
O15 1st reg.							
O15 2nd reg.							
O16 primary							
O16 1st reg.							
O16 2nd reg.							

Missing data are mostly due to adults maturing prematurely or dying accidentally

Frequency of patterns (= primary clones)

	segmental ectoderm	median neural	pygidial ectoderm	segmental mesoderm	pygidial mesoderm	endoderm	amoeboid cells
ubiquitous expression	7	4	6	3		4	
stochastic expression	21		1			2	4
partially transgenic	1	9	46	3	1	10	
Total	29	13	53	6	1	16	4

Total positive	Total survivors
62	87

Transmission of transgenic expression through regeneration

		segmental ectoderm before regeneration	segmental ectoderm after regeneration
segmental ectoderm	transgenic	39	39
	non transgenic	38	38
		pygidial ectoderm before regeneration	pygidial ectoderm after regeneration
segmental ectoderm	transgenic	39	39
	non transgenic	38	38
		before regeneration	after regeneration
segmental mesoderm	transgenic	11	11
	non transgenic	66	66
		before regeneration	after regeneration
endoderm	transgenic	23	22
	non transgenic	54	54

Total experiments
77
