# Supplementary material for: Molecular profiles, sources and lineage restrictions of stem cells in an annelid regeneration model
Source: Nat Commun. 2024 Nov 18;15:9882. doi: 10.1038/s41467-024-54041-3 (PMC11574210; doi:10.1038/s41467-024-54041-3)
Supplement: Supplementary file 11 — Reporting Summary [file 41467_2024_54041_MOESM11_ESM.pdf]

nature portfolio

Corresponding author(s): Guillaume Balavoine and Florian Raible

Last updated by author(s): 22/07/2024

Reporting Summary

Nature Portfolio wishes to improve the reproducibility of the work that we publish. This form provides structure for consistency and transparency in reporting. For further information on Nature Portfolio policies, see our Editorial Policies and the Editorial Policy Checklist.

Statistics

For all statistical analyses, confirm that the following items are present in the figure legend, table legend, main text, or Methods section.

n/a	Confirmed	
☐	☒	The exact sample size (*n*) for each experimental group/condition, given as a discrete number and unit of measurement
☐	☒	A statement on whether measurements were taken from distinct samples or whether the same sample was measured repeatedly
☐	☒	The statistical test(s) used AND whether they are one- or two-sided<br>*Only common tests should be described solely by name; describe more complex techniques in the Methods section.*
☒	☐	A description of all covariates tested
☐	☒	A description of any assumptions or corrections, such as tests of normality and adjustment for multiple comparisons
☒	☐	A full description of the statistical parameters including central tendency (e.g. means) or other basic estimates (e.g. regression coefficient) AND variation (e.g. standard deviation) or associated estimates of uncertainty (e.g. confidence intervals)
☐	☒	For null hypothesis testing, the test statistic (e.g. *F*, *t*, *r*) with confidence intervals, effect sizes, degrees of freedom and *P* value noted<br>*Give P values as exact values whenever suitable.*
☒	☐	For Bayesian analysis, information on the choice of priors and Markov chain Monte Carlo settings
☒	☐	For hierarchical and complex designs, identification of the appropriate level for tests and full reporting of outcomes
☐	☒	Estimates of effect sizes (e.g. Cohen's *d*, Pearson's *r*), indicating how they were calculated

Our web collection on statistics for biologists contains articles on many of the points above.

Software and code

Policy information about availability of computer code

Data collection	ZEN 3.3
Data analysis	cutadapt (v. 1.12), fastQC (v. 0.11.9), multiQC (v. 1.14), STAR aligner (v. 2.7.10b), featurecounts (v. 2.0.1), DESeq2 (v. 1.36.0), mfuzz (v. 2.56.0), ggplot2 (v. 3.4.2), cellranger (v. 7.0.1), Seurat (v. 4.3.0), BLASTX, BLASTP, IQ-Tree (v. 1.6.12), CLC Main Workbench Software package (v. 23.0.2), iTOL Suite, SCpubr (v. 1.1.2), HCR3.0 Probe Maker (v. 2021_0.3.2), Fiji, GraphPad Prism (v 10.2.2)

For manuscripts utilizing custom algorithms or software that are central to the research but not yet described in published literature, software must be made available to editors and reviewers. We strongly encourage code deposition in a community repository (e.g. GitHub). See the Nature Portfolio guidelines for submitting code & software for further information.

Data

Policy information about availability of data

All manuscripts must include a data availability statement. This statement should provide the following information, where applicable:

- Accession codes, unique identifiers, or web links for publicly available datasets
- A description of any restrictions on data availability
- For clinical datasets or third party data, please ensure that the statement adheres to our policy

All data will be made available and non-restricted before publication. The research does not contain clinical dataset or third party data.

Research involving human participants, their data, or biological material

Policy information about studies with human participants or human data. See also policy information about sex, gender (identity/presentation), and sexual orientation and race, ethnicity and racism.

Reporting on sex and gender	NA
Reporting on race, ethnicity, or other socially relevant groupings	NA
Population characteristics	NA
Recruitment	NA
Ethics oversight	NA

Note that full information on the approval of the study protocol must also be provided in the manuscript.

Field-specific reporting

Please select the one below that is the best fit for your research. If you are not sure, read the appropriate sections before making your selection.

☒ Life sciences ☐ Behavioural & social sciences ☐ Ecological, evolutionary & environmental sciences

For a reference copy of the document with all sections, see nature.com/documents/nr-reporting-summary-flat.pdf

Life sciences study design

All studies must disclose on these points even when the disclosure is negative.

Sample size	At the time of the experiments, the variance was unknown. For TOR inhibition experiments (Fig. 5), we therefore selected a sample size (6 invertebrate polychaete worms) that is feasible with the experimental resources, and that is common in the research field. Likewise, for assessing nuclear and nucleolar sizes (Fig. 2f,g), three independent individuals were analysed.
Data exclusions	No sample was excluded from the data
Replication	All attempts of replication were successful.
Randomization	Polychaete animals (Platynereis dumerilii) of the PIN strain were randomly selected from sibling- or closely related batches of similar age and developmental stage.
Blinding	The quantifications and phenotype comparisons were performed upon blinding.

Reporting for specific materials, systems and methods

We require information from authors about some types of materials, experimental systems and methods used in many studies. Here, indicate whether each material, system or method listed is relevant to your study. If you are not sure if a list item applies to your research, read the appropriate section before selecting a response.

Materials & experimental systems

n/a	Involved in the study	
☒	☐	Antibodies
☒	☐	Eukaryotic cell lines
☒	☐	Palaeontology and archaeology
☐	☒	Animals and other organisms
☒	☐	Clinical data
☒	☐	Dual use research of concern
☒	☐	Plants

Methods

n/a	Involved in the study	
☒	☐	ChIP-seq
☒	☐	Flow cytometry
☒	☐	MRI-based neuroimaging

Animals and other research organisms

Policy information about studies involving animals; ARRIVE guidelines recommended for reporting animal research, and Sex and Gender in Research

Laboratory animals	Platynereis dumerilii worms, inbred wild-type strain from the Vienna collection (PIN)
Wild animals	NA
Reporting on sex	NA
Field-collected samples	NA
Ethics oversight	In compliance with the relevant Directive 2010/63/EU on the protection of animals used for scientific purposes, the "3R" principles were obeyed. No specific ethical approval was required because all research is done on invertebrate polychaete worms.

Note that full information on the approval of the study protocol must also be provided in the manuscript.
